# Characterizing Human Habits in the Lab

**DOI:** 10.1525/collabra.92949

**Published:** 2024-02-28

**Authors:** Stephan Nebe, André Kretzschmar, Maike C. Brandt, Philippe N. Tobler

**Affiliations:** 1 Zurich Center for Neuroeconomics, Department of Economics, University of Zurich, Switzerland; 2 Individual Differences and Assessment, Department of Psychology, University of Zurich, Switzerland

**Keywords:** habit, value-based decision making, goal-directed control, computational modeling, training

## Abstract

Habits pose a fundamental puzzle for those aiming to understand human behavior. They pervade our everyday lives and dominate some forms of psychopathology but are extremely hard to elicit in the lab. In this Registered Report, we developed novel experimental paradigms grounded in computational models, which suggest that habit strength should be proportional to the frequency of behavior and, in contrast to previous research, independent of value. Specifically, we manipulated how often participants performed responses in two tasks varying action repetition without, or separately from, variations in value. Moreover, we asked how this frequency-based habitization related to value-based operationalizations of habit and self-reported propensities for habitual behavior in real life. We find that choice frequency during training increases habit strength at test and that this form of habit shows little relation to value-based operationalizations of habit. Our findings empirically ground a novel perspective on the constituents of habits and suggest that habits may arise in the absence of external reinforcement. We further find no evidence for an overlap between different experimental approaches to measuring habits and no associations with self-reported real-life habits. Thus, our findings call for a rigorous reassessment of our understanding and measurement of human habitual behavior in the lab.

“So far as we are thus mere bundles of habit, we are stereotyped creatures, imitators and copiers of our past selves” (James, 1899, p. 66).

We often think of our behavior as goal-directed and purposeful. Yet, research suggests that a large part of our everyday behavior is habitual rather than goal-directed (Verplanken & Orbell, 2003). Habits are slowly and incrementally learned stimulus-response associations. They are inflexible and insensitive to sudden changes in the environment like the devaluation of reinforcers or the degradation of response-outcome contingencies (Dickinson & Pérez, 2018; Seger & Spiering, 2011; Verplanken, 2018). Of course, most habits can eventually be modified after changing the contingencies between actions and their outcomes or the values of these outcomes, but these adaptations again need time to evolve (Carden & Wood, 2018; De Houwer, 2019; Gardner, 2015; Kruglanski & Szumowska, 2020). Capitalizing on the slow adaptation of learned habits and their automatic triggering by contextual factors, previous research has associated habitual control with a wide range of health-relevant processes and behaviors that are repeated frequently, such as dietary decisions (Carels et al., 2014; Gardner et al., 2014; Lally et al., 2008), physical exercise (Fleig et al., 2013; Kaushal & Rhodes, 2015; Verplanken & Melkevik, 2008), and social network website use (Lindström et al., 2021; Turel & Bechara, 2016). Moreover, mounting evidence suggests that pathologically habitual decision making characterizes substance use and obsessive-compulsive disorders (Gillan et al., 2016; Lüscher et al., 2020; Voon et al., 2014), although there is also conflicting evidence regarding this relationship (Gillan, 2021; Nebe et al., 2018; Sebold et al., 2017). However, habits are not maladaptive as such. They free cognitive resources for other tasks because stimuli trigger behavior in a computationally simple and effortless manner. Moreover, habitization can also be used to gradually shape advantageous human behavior over time, for example brushing teeth after a meal, recycling waste, or quitting smoking (Baldwin et al., 2006; Gardner et al., 2019; Marteau et al., 2012; Rothman et al., 2009). Thus, it is crucial to know how habits develop and to obtain a handle on how to promote the evolution of beneficial habits and change detrimental ones.

Unfortunately, it has proven surprisingly difficult to study habits in the lab, at least with humans (de Wit et al., 2018). Given their definition as stimulus-response associations, habits are blind to changes in the value of the outcomes they produce. Therefore, classical examinations of habitual control used experimental paradigms that extensively trained an instrumental response in stable conditions. Such (over)training leads to a shift from goal-directed (flexible but cognitively demanding) to habitual control and reduced sensitivity to reductions in the outcome’s value or the contingency between action and outcome (Adams & Dickinson, 1981; Dickinson, 1985). Over the last decades, animal research used satiation on specific food rewards, paired the rewards with illness-inducing injections after learning, or provided additional outcomes without requiring an action and showed a reduced capacity of these outcome devaluation or contingency degradation tests to change habitual responding (Adams & Dickinson, 1981; Dickinson & de Wit, 2003; but see Garr et al., 2020, 2021; Thrailkill et al., 2018 for recent conflicting findings). In contrast, only one study was able to show habitization of behavior via overtraining in a human laboratory paradigm (Tricomi et al., 2009). However, this finding could not be replicated in two studies with larger sample sizes, questioning the validity of the original findings and arguing that the classic paradigms with human participants show at best a moderate role of habits in behavioral control (de Wit et al., 2018; but see Pool et al., 2021). In fact, when these classic paradigms and their variants are used to investigate habits in humans they arguably tap primarily into goal-directed control as the test procedures are too salient and the training is usually not extensive enough to form habits (de Wit et al., 2018; Watson & de Wit, 2018). These issues raise the question whether we can induce habits in the lab at all.

In recent years, some lines of research have increasingly focused on an alternative approach to studying habits in humans that circumvents the difficulties of inducing habits experimentally and instead relies on computational modelling of choices in sequential Markov decision tasks (Daw et al., 2011; Decker et al., 2016; Gläscher et al., 2010; Kool et al., 2016). In this approach, habitual and goal-directed behavioral control are operationalized as model-free and model-based reinforcement learning, respectively (Daw et al., 2005). In essence, model-free reinforcement learning works by summing up previously received rewards and increasing the probability of selecting actions that have led to (more) reward, with the aim of maximizing rewards in the long run (Dolan & Dayan, 2013; Sutton & Barto, 1998). Model-free learning can capture behavior change well. For example, giving a dog a treat every time it obeys an order increases the probability that it will follow the order in the future, even when the treats will be withheld at some point. However, as can be seen from this example, model-free reinforcement learning is entirely driven by the incentive value of outcomes associated with behavior (Kruglanski & Szumowska, 2020). Thus, although this approach aims to operationalize habitual behavior, model-free learning concerns reinforcement and therefore fails to capture the outcome-independent nature of habits (Miller et al., 2019). By extension, although model-free reinforcement learning might constitute one candidate process to develop habits, habitual behavior has little in common with model-free reinforcement learning once habits are established.

It is worth noting that the sequential Markov decision tasks used to assess model-based and model-free learning feature a permanently changing task environment (because reinforcement probabilities follow random walks over the course of the task), which is thought to counter the development of habits (e.g., Gardner, 2015; Ouellette & Wood, 1998; Verplanken & Wood, 2006). Indeed, previous studies investigating the overlap of model-free reinforcement learning and insensitivity to reinforcer devaluation after training found only statistically non-significant small to very small associations, which refutes the notion that they both operationalize habitual behavior (Friedel et al., 2014; Gillan et al., 2015; Sjoerds et al., 2016). Yet, model-free reinforcement learning is still portrayed as a valid approximation of habitual behavior (e.g., Drummond & Niv, 2020; Patzelt et al., 2018; Wyckmans et al., 2019) calling for an adequately powered empirical test of the validity of this task for measuring habits.

Using computational simulations, Miller and colleagues (2019) recently proposed that a frequency-based habitization process could reproduce hallmark findings of choice and perseveration in the experimental literature on habits (Guthrie, 1959; see also Schwöbel et al., 2021, for a Bayesian implementation of repetition-based habit learning). This view converges with evidence for frequency-related processes playing a role in various psychological processes. Examples include the Hebb effect in working memory (Oberauer et al., 2015), choice history bias in perceptual decision making (Abrahamyan et al., 2016; Braun et al., 2018; Urai et al., 2019), the mere-(repeated-)exposure effect (Zajonc, 2001), and choice-induced preference changes in value-based decision making (Brehm, 1956; Izuma & Murayama, 2013; Sharot et al., 2009). Some studies on choice-induced preference change (Izuma et al., 2015; Izuma & Murayama, 2013; Sharot et al., 2010) suggest that selecting a stimulus may change its value even if decision makers did not perceive the outcome of their choice. This raises the question whether the experience of an outcome following an action is indeed necessary to induce habits or whether habits can arise simply from frequently performing an action. More generally, independent lines of research substantiate the hypothesis that there is a basic learning process based on the frequency of behavior, which can mechanistically change psychological variables (e.g., memory representations, subjective values) and that such a frequency-based process might be independent of reinforcement. By consequence it is conceivable that processes induced by frequently repeated choice contribute to habitual behavior.

Mounting evidence (Hardwick et al., 2019; Luque et al., 2020) suggests that with fast response times (up to about 600 ms) habitual response tendencies cannot be overruled by goal-directed processes and by extension that time pressure is important to unmask habits. However, it remains an open empirical question whether a frequency-based learning process changes an underlying psychological variable driving behavior (such as the subjective value of the stimuli which trigger responses) and whether the process is completely independent of reinforcement. Here, we aim to investigate frequency-based habitization both with explicit reinforcement of behavior and without because it is unclear whether external reinforcement is necessary for habits to form. Furthermore, we examine the validity of various experimental approaches to habits using six different behavioral paradigms – two newly developed and piloted paradigms and four previously used tasks including traditionally used outcome devaluation (Luque et al., 2020; Tricomi et al., 2009) and contingency degradation tasks (Vaghi et al., 2019) as well as a modified version of the sequential Markov decision task (Kool et al., 2016). Our study addresses the issue that most previous research neglected to train participants extensively enough to induce habits, as habits are supposed to grow stronger with repetition. Specifically, we extend training to four or five days for all tasks except the sequential Markov decision task, which has been shown to lead to stronger model-based choice tendencies with extended training (Economides et al., 2015) and one of the outcome devaluation paradigms (Tricomi et al., 2009), in which there seems to be no increase of habitual behavior with extended training (Pool et al., 2021). Our two tasks apply design features thought to favor the development of habitual behavior, that is, stability of the task environment, responding under time pressure, and extended training over several days (de Wit et al., 2018; Luque et al., 2020; Watson & de Wit, 2018; Wood & Neal, 2009). Finally, to externally validate the previously established tasks we collect reports of habitual behavior in real-life. This aspect of our study advances the field independently of the question whether frequency-based habit formation exists. Specifically, our study strives to answer the following Research Questions (RQ).

*RQ1: Does a frequency-based habitization process exist?* As outlined, the mere frequency of behavior might be the driving force of habitization, leading to a gradual change of subjective value proportional to choice frequency. However, current experimental paradigms do not allow separating the influence of behavioral frequency from subjective value. In these tasks, choice frequency typically correlates with outcome value, impeding differentiation of their respective effects. Thus, we designed a reinforcement learning paradigm that manipulates the value of options and their choice frequency during training independently (Reward Pairs task). This feature of the task allows us to disentangle the influence of value-based and frequency-based processes during learning and decision making in the reinforced training sessions and a subsequent non-reinforced test phase.

Hypothesis 1: Higher frequency of choosing stimuli during the training of the Reward Pairs task is associated with more strongly habitual behavior during the test phase (Table 1 for the operational specifications of all hypotheses).

*RQ2: Is external reinforcement of behavior necessary for frequency-based habitization or can the process arise also in a context without external reinforcement?* According to the notion that the frequency-based habitization process is not dependent on reinforcement, we should be able to elicit habits in an experimental task without any external reinforcement of behavior. This would constitute a proof of concept that choice frequency alone can drive habitization of behavior. Conversely, if frequency-based habits develop only in externally reinforced contexts, their underlying function might be to enhance reinforcement value learning instead of being a process completely divorced from reinforcement. To address this issue, we designed a novel paradigm that manipulates instructed choice frequency but is completely free from external reinforcement (Unrewarded Habit task).

Hypothesis 2: Higher frequency of choosing stimuli during the training of the Unrewarded Habit task is associated with more strongly habitual behavior during the test phase.

*RQ3: Is there a universal (i.e*., *paradigm independent) habitization process?* As pointed out previously (Friedel et al., 2014), different paradigms to study habits are considered equivalent, but there is almost no empirical evidence for this assumption. If anything, the few studies that have addressed the topic suggested that findings are rather dependent on the operationalizations used (Friedel et al., 2014; Gillan et al., 2015; Sjoerds et al., 2016), which makes it impossible to generalize to other paradigms or to draw generally valid conclusions regarding the habitization process (construct validity; Cronbach & Meehl, 1955). Therefore, we investigate the association of frequency-based habitization as measured in our two tasks with previously used paradigms of habitual behavior, that is, devaluation insensitivity in two outcome devaluation tasks (Luque et al., 2020; Tricomi et al., 2009) and insensitivity to changes in the action-outcome contingency in a contingency degradation task (Vaghi et al., 2019). We also asses model-free reinforcement learning in a sequential Markov decision task (2-Step; Kool et al., 2016). Investigating the relations between six different paradigms allows us to draw conclusions about the generality of the habitization processes (i.e., the extent to which the paradigms measure a similar form of habit formation). As outlined above, insensitivity to outcome devaluation and model-free reinforcement learning in sequential decision tasks might rely too heavily on goal-directed control and representation of reinforcement values, respectively, to be a pure measure of habitual behavior (de Wit et al., 2018; Miller et al., 2019; Watson & de Wit, 2018). It is yet unclear whether contingency degradation tasks have the same drawback. One may argue that delivering unexpected outcomes independent from actions to degrade the contingency between action and outcome might be a similarly salient manipulation as the devaluation of outcomes in other experimental paradigms. We test the assumption that the different operationalizations of habits in the different paradigms capture the same habitization process, which is what the current literature implies by using all experimental tasks interchangeably to examine habit formation. Additionally, we test the discriminant validity of the habitization process by exploring its association with working memory capacity. Working memory is an executive function, which may play a fundamental role for employing goal-directed behavior. Because habitual behavioral control has been opposed to goal-directed behavior (Dolan & Dayan, 2013) and has low cognitive demand, we expect a relatively weak association between working memory capacity and habitual behavior.

Using the modified version of the Markov decision task (Kool et al., 2016) instead of the original 2-Step task for this Research Question also enables us to conceptually replicate previous studies of the overlap between devaluation insensitivity and model-free reinforcement learning (Friedel et al., 2014; Gillan et al., 2015; Sjoerds et al., 2016) and extend it to a wider range of experimental operationalizations.

Hypothesis 3.1: The measures of habitual behavior in all six habit paradigms (i.e., Reward Pairs, Unrewarded Habit, outcome devaluation (Tricomi and Luque), contingency degradation, and 2-Step task) are positively associated with each other (i.e., convergent validity) and, thus, the corresponding data fits a one-dimensional measurement model.

Hypothesis 3.2: The measures of habitual behavior in the habit paradigms are more strongly correlated with each other than with working memory (i.e., discriminant validity) and, thus, the data fits a two-dimensional measurement model significantly better than a one-dimensional measurement model.

*RQ4: How does the habitization process measured with the experimental paradigms relate to real-life habitual behavior?* The concordance between experimentally induced and real-life habits is often overlooked but essential to provide practical meaning to laboratory findings (criterion validity; Cronbach & Meehl, 1955). To the best of our knowledge, there are only two studies that have empirically investigated the relation between habits measured in experimental paradigms in the laboratory and those from everyday life. One study showed an association between action slips after outcome devaluation and decreased attention during (but no association with mistakes made, time or effort spent in) real-life habitual behavior (Linnebank et al., 2018). Another study recently reported a negative association between sensitivity to contingency degradation and self-reported automaticity (Ersche et al., 2021). To add to this, we test the associations of all six habit paradigms with self-reported real-life habitual behavior as measured with validated self-report questionnaires of real-life habits. For this purpose, we use the Self-Report Habit Index (SRHI; Verplanken & Orbell, 2003), the Creature of Habit Scale (COHS; Ersche et al., 2017), and the Habitual Tendencies Questionnaire (HTQ; Ramakrishnan et al., 2021). Responses to the SRHI revealed relations with actual observations of real-life habits in previous studies (Gardner et al., 2012; Lally et al., 2010). Thus, we investigate to what extent a universal habitization process is related to real-life habitual behavior. In addition, we explore and compare the criterion validity of each employed experimental paradigm. Our analyses enable us to quantify the degree of criterion validity of each paradigm. Comparing them to each other allows us to make an empirically grounded recommendation for future studies aiming to use the most appropriate experimental operationalization of habits. In case of conflicting results regarding the associations of experimental measures with the three questionnaires, the association with the SRHI are regarded most valid, as this questionnaire has the most extensive empirical evidence for its validity of the three.

Hypothesis 4: The habitization process measured in the experimental paradigms is positively associated with self-reported real-life habitual behavior.

Research Questions 3 and 4 focus on the generalizability and construct validity of different habit paradigms, that is, whether the habitization process is independent of specific paradigms used in laboratory research as well as the significance of this process for everyday behavior. Hence, these findings provide a complete picture of the validity of our approaches to measure human habits in the lab. However, the use of several newly developed as well as previously used paradigms provides the possibility to tackle another exploratory Research Question, which is concerned with the most appropriate operationalization of habits in laboratory research.

*RQ5: Is there a difference in validity with regard to various behavioral measures of habitual behavior?* From a clinical perspective, overt choices constitute the most relevant measure of task performance. We also analyze response times (RTs), stimulus ratings, and computational model parameters as potential expressions of habits. We expect the habitization process to be reflected to some extent in all of these behavioral measures. Recent research (Luque et al., 2020) suggested RTs to be a more sensitive measure of habits than overt choices in an outcome devaluation paradigm. Our final Research Question thus intersects all previous hypotheses insofar as we examine possible differences in the results for each Research Question depending on different behavioral measures of habitization (i.e., choices, RTs, ratings, and parameters of computational models). Therefore, all the analyses of the previously mentioned hypotheses are performed separately for each applicable behavioral measure (see Table S2 for an overview of available behavioral measures per paradigm). For example, as all four behavioral measures are available for the Reward Pairs task, the analysis of Hypothesis 1 is done four times, each time with a different behavioral measure. While we consider positive evidence for one of the hypotheses nested in RQ1 and RQ2 sufficient to support it, we examine systematic patterns in the findings in RQ5. As previous findings do not provide a consistent picture regarding the validity of the behavioral measures, we have no explicit expectations, but consider this Research Question to be purely exploratory. The results are used to provide best practice recommendations for the operationalization of habits in the laboratory for future research.

## Methods

### Ethics information and participant compensation

Participants were informed about the study procedure, their right to withdraw their consent at any time, data security and the use of their anonymized data (including public data sharing upon study completion). They then gave written informed consent. The study was approved by the local ethics committee (Human Subjects Committee of the Faculty of Economics, Business Administration, and Information Technology; OEC IRB # 2020-072).

The participants received CHF 20 (at the time of testing, CHF 1 ~ US$ 1.10 ~ € 1) per hour of testing for each session. In addition, they received money from their performance in the monetarily reinforced tasks (Reward Pairs task: CHF 0.50-4.50 each day plus CHF 1-9 for the test phase on the last day; Contingency Degradation task: CHF 0-6.84 each day; Outcome Devaluation task_Luque_: CHF 0.15-5.00 each day; 2-step task: CHF 10-13). This amounted to a pay-out of CHF 194-272 with an expected average payment of CHF 235 according to pilot data. Participants who withdrew from the study were compensated with CHF 25 for each day they attended fully and CHF 10 for every hour started on days they did not complete.

### Design

The study included one week of data collection. It started with an invitation email with a sign-up link, followed by participants receiving a study description and giving informed consent. They then filled in a sociodemographic questionnaire, the German versions of the Creature of Habit Questionnaire (COHS; Ersche et al., 2017), Self-Report Habit Index (SRHI; Verplanken & Orbell, 2003), Habitual Tendencies Questionnaire (HTQ; Ramakrishnan et al., 2021), Edinburgh Handedness Inventory (EHI; Oldfield, 1971), and Social Desirability Scale (SDS-17; Stöber, 1999, 2001). In addition, they completed a version of a sequential Markov decision paradigm assessing model-free and model-based behavior (2-Step; Kool et al., 2016). Within one week before the training of the habit tasks began, participants had to fill in these questionnaires and perform the 2-Step task online to reduce the amount of time spent in the lab. The COHS, SRHI, and HTQ were used as real-life habit measures to validate the experimental measures of habitual behavior. The handedness score of the EHI was used in analyses of RTs to control for generally faster responses with the preferred hand. The SDS-17 was used to control for tendencies to respond in a socially desirable manner in the test phase of the Unrewarded Habit task if necessary. The sociodemographic information was only used to characterize our sample.

As the study procedure was quite extensive, we applied a planned-missing design (Graham et al., 2006; Little et al., 2014; Rhemtulla & Hancock, 2016). In these designs, the complete sample is randomly split into several subgroups. Each subgroup performs one set of experimental tasks and questionnaires, which is common to all participants, and leaves out one task. For each participant, the left-out task is random. Hence, the missing data is missing completely at random (MCAR) and can be imputed for analyses across tasks on the basis of the data acquired from each participant (see Analysis plan). In our case, we randomly assigned participants to one of two subgroups. Each subgroup performed all questionnaires and tasks except for one (either the outcome devaluation task_Luque_ (Luque et al., 2020) or the contingency degradation (Vaghi et al., 2019) task). Therefore, the required assessment time for each participant was reduced, decreasing their individual workload.

The first on-site assessment started with three working memory tasks (Lewandowsky et al., 2010), which took approximately 25 min to perform, followed by the first outcome devaluation task (45 min; Pool et al., 2021; Tricomi et al., 2009). Next, all participants performed the Unrewarded Habit task (21 min) and Reward Pairs task (18 min) in random order. These two tasks were repeated daily until and including the fifth day (see Table S1 for an overview of the study procedure). In addition, each participant performed one of the two remaining tasks, that is, either the contingency degradation task (35min; Vaghi et al., 2019) or the second outcome devaluation paradigm (35min; Luque et al., 2017, 2020). Training of the contingency degradation or the second outcome devaluation task started on the second day of on-site assessment and proceeded daily until and including the fifth day. A short break of 1-3 min was included between the tasks. On the fifth day, the training phases of the tasks were followed or replaced by the respective test phases. The fifth day closed with a debriefing questionnaire asking for choice strategies during the test phases of the habit paradigms and paying out the participants. The first session lasted around 110 min, the second to fourth session around 65 min, the fifth session around 85 min, and the online assessment prior to the first assessment around 90 min.

The tasks were trained over several days (Table S1) with order randomized across participants but constant within participants to control for effects of motivation changes within a training session. All tasks and questionnaires were applied in German. For the following description of the materials, the wording was translated into English.

### Tasks

#### Selection of Stimuli for Reward Pairs and Unrewarded Habit Task

Change in subjective value ratings was an outcome variable of interest for the Reward Pairs and the Unrewarded Habits tasks. We used pre-training ratings also to select stimuli for these tasks. Specifically, participants rated a set of 15 geometric shapes (Reward Pairs task) and 15 abstract figures (Unrewarded Habit task) twice before the first training session. They were asked “How do you like the symbol on this box?” and used a slider to respond on a continuous visual analogue scale ranging from 0 to 100 with the verbal anchor points of “not at all” (0), “neutral” (50), and “extremely well” (100). The eight (Reward Pairs task) or four (Unrewarded Habit task) individually most neutrally rated stimuli (averaged over the two ratings per stimulus) were used for the experiment. Using an individual set of stimuli for the tasks served two purposes. First, it controlled for pre-experiment preferences between the given stimuli. Second, it limited stimulus-specific effects (Clark, 1973; Judd et al., 2012). We controlled for remaining stimulus-specific variance (Baayen et al., 2008) by using a random-effects model (see Analysis plan).

#### Reward Pairs Task

**Figure 1:**
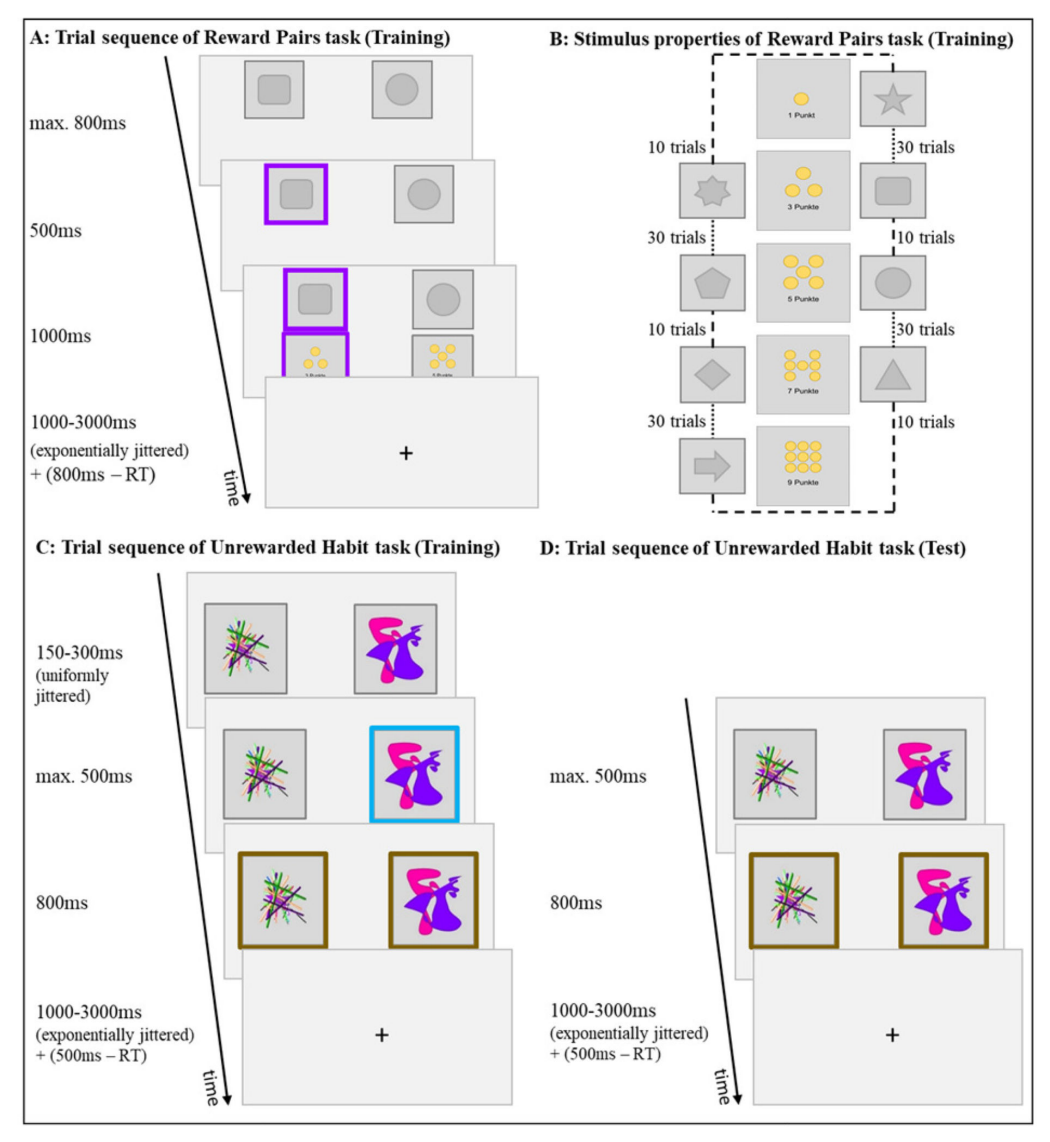
Reward Pairs [A, B] and Unrewarded Habit [C, D] tasks. **A** Trial sequence of the training phase of the Reward Pairs task. Each trial started with the presentation of two stimuli. The selected stimulus was indicated by a purple frame. Then both stimuli and their respective rewards were shown. A fixation cross was presented in the center of the screen during the exponentially jittered inter-trial-interval, which also included the remainder of the response time window. The trial sequence and timing of the test phase (not shown) was similar but did not display the rewards. **B** Example stimulus-to-reward assignment of the Reward Pairs task, showing eight stimuli (geometric shapes), five reward levels (1, 3, 5, 7, and 9 points; yellow points), and the respective number of training trials per training session. For example, the pentagon and circle were both worth five points, while the triangle was worth seven points. The circle was presented with the square in ten trials and with the triangle in 30 trials during training. Thus, stimuli of the same reward level (e.g., 5 points) were chosen with different frequencies (e.g., 30 times for the pentagon and ten times for the circle) for reward maximizing decision makers. **C** Trial sequence of the training phase of the Unrewarded Habit task. Each trial of the training phase started with the presentation of two stimuli. A blue frame appeared around one of the stimuli instructing participants to select this stimulus. A brown frame indicated both the chosen and unchosen stimulus. During the exponentially jittered inter-trial-interval, which also included the remainder of the response time window, a fixation cross was presented in the center of the screen. **D** Test phase of the Unrewarded Habit task. Trials followed a similar sequence as the training trials but lacked the blue frame to instruct participants which stimulus to choose. Thus, when the two stimuli appeared on the screen, participants selected one of them freely. RT – response time.

The first task was an instrumental learning paradigm, in which we manipulated the reward value of stimuli and their choice frequency during training independently from each other. Each trial showed one stimulus on the left side of the screen and one on the right side (Figure 1A). Crucially, the task comprised pairs of stimuli, not presented together during training, where both stimuli had the same objective value but one of them had been presented often with a less valuable stimulus and therefore chosen more often during training. Conversely, the other stimulus of the pair had been presented primarily with a more valuable stimulus and therefore chosen less often during training. When the two same-reward stimuli were presented together during test, we examined whether the difference in choice frequency during training had an effect on choice during test. Participants had a response window of 800 ms to choose one of them by pressing either “X” or “M” on the keyboard. A purple frame indicated the chosen stimulus for 500 ms, before both stimuli with their respective outcomes appeared for 1000 ms. A purple frame indicated the chosen stimulus and the reward associated with it. The subsequent inter-trial interval (ITI; 1000-3000 ms) was exponentially jittered and the remainder of the response window was added to it to hold the length of each trial constant. Training took place on each of five consecutive days with 160 trials per session.

There were eight stimuli in the Reward Pairs task. Each stimulus deterministically yielded a reward of 1, 3, 5, 7, or 9 points (Figure 1B). Participants learned the contingencies between stimuli and rewards through trial-and-error. To facilitate learning and reduce uncertainty about the rewards associated with rarely chosen stimuli, participants received feedback about the outcomes of both the chosen and unchosen stimulus after each decision in the training phase (but not in the test phase, see below). Delivering rewards deterministically instead of stochastically should increase the transparency and stability of the task environment and thereby facilitate the development of habits.

For three stimulus pairs, the stimuli yielded the same amount of reward (3, 5, or 7 points), and these same-reward pairs never appeared together during training. For each same-reward pair, reward maximizing decision makers should choose one stimulus more often than the other because during training, one stimulus was frequently paired with a stimulus providing two points less or a stimulus providing two points more. Specifically, each stimulus was shown 40 times in one training session – either 10 times with a less valuable stimulus and 30 times with a more valuable stimulus or vice versa (see Table S4 for a list of all stimulus pairings during one training session). In consequence, stimuli within pairs were chosen either more or less frequently as long as participants adhered to the instruction to maximize points earned. For example, within say the 5 points pair, one stimulus was paired 30 times with a stimulus worth 3 points and 10 times with a stimulus worth 7 points and the other way around for the other stimulus (Figure 1B). Hence, for the three intermediate reward levels (i.e., 3, 5, and 7 points), there was one stimulus chosen frequently during training and the other chosen rarely, which disentangles stimulus value and choice frequency. Stimulus-to-position assignments were matched for left and right positions across stimuli, such that the number of choices at each position was similar. In total, participants could earn between 640 and 960 points in one training session, and they were instructed that one trial was randomly selected each day and the reward associated with their choice added to the final pay-out with 1 point being worth CHF 0.5.

The trial sequence of the test phase immediately after the fifth training session was similar to the training phase: each trial started with the presentation of two stimuli, giving participants 800 ms to make a selection via button press. Left and right presentation was balanced for each stimulus within participants. The chosen stimulus was framed for 500 ms. The reward was not shown to prevent further learning. Each stimulus was presented with each other stimulus four times. Together with an additional eight trials for each of the three same-reward pairs, the test phase comprised 136 trials. The remainder of the response window was again added to the ITI to keep the duration of each trial constant.

Note that the test phase presented the stimuli of the same-reward pairs together for the first time since participants associated them with reward. Both stimuli of the same-reward pairs should have identical cached values after the prolonged training and based on value participants should be indifferent between them. However, if habitual behavior can develop from repeating one action more often, then stimuli chosen more frequently during training should be preferred during test. Between 248 and 424 points could be earned in the test phase. Participants were instructed to keep on maximizing the points earned and that two trials of the test phase would be chosen randomly at the end and paid out, with 1 point worth CHF 0.5.

After the first rating, participants completed a free-choice phase without outcome presentation for both tasks. Participants were instructed to choose the stimuli they liked better, and that their choices in this phase had no consequences but served to familiarize them with the test situation and to experience the short decision time windows that would be used in the real experiments. With this procedure, we could test whether value and frequency differences experienced in training affected choices. Choices in this phase revealed pre-experiment preferences between the stimuli, which can be existent even if the stimuli have been rated similarly before (Chen & Risen, 2010). Note that we used the most neutrally rated stimuli per participant, but these did not have to be exactly neutral. Participants were not excluded from the study for not rating enough stimuli in the neutral middle region of the rating scale, but the analyses of choice data were corrected for pre-experiment stimulus preferences including both the rating and the choice in the free-choice phase. A second rating of all 30 (i.e., used and unused) stimuli (also twice per stimulus) after the test phase on the last day allowed us to test whether choice frequency during training changed the subjective value of stimuli. Furthermore, participants rated 10 additional abstract stimuli at the beginning and end of the study, which were not used in any task for any participant. We used these to estimate how reliable stimulus ratings were in general.

After the test phase and the second stimulus rating, participants completed debriefing questions. These questions concerned the reward value of each stimulus and how confident participants were about their answers. Most participants of the pilot study revealed perfect knowledge about the reward value for each stimulus. This finding is a prerequisite to interpret differences in choice frequency of stimuli within the same reward level as resulting from different previous choice proportions during training rather than from larger uncertainty about the value of less frequently chosen stimuli. Therefore, we excluded participants from our analyses with less than 50% accuracy in the debriefing question about stimulus-reward associations.

The outcome measures of interest were the observed choice frequencies and RTs during the test phase and the change of the liking ratings from before the training to after the test phase. A frequency-based habit supposedly acquired during training would decrease RTs and increase preferences for, and ratings of, more frequently chosen stimuli. In addition, computational models of choices during the training and test phases including a choice kernel (see Analysis plan) should be favored over models based solely on the reinforcement value of stimuli or random choices. With this, we deviated from the traditional approach of using outcome devaluation or contingency degradation to directly test the absence of the goal and/or instrumental criterion of goal-directed behavior (Dickinson & Balleine, 1994), as these direct tests might be too salient for many human participants. Instead, we tested the potential effect of choice frequency during training on behavior during test.

To associate behavior in the Reward Pairs task to behavior in other tasks and answer RQs 3 and 4, we computed an individual score of frequency-based habitual choice from the test phase (*Reward Pairs choice score*). This procedure was similar to scores computed from choices in sequential decision and outcome devaluation paradigms (e.g., Linnebank et al., 2018; Luque et al., 2020; Nebe et al., 2018). The score captured the propensity of participants to choose stimuli during test which they chose more (rather than less) frequently during training, for each of the same-reward pairs. *Reward Pairs choice scores* closer to the maximum value of 1 reflected stronger preference for the more frequently chosen stimuli, scores around zero corresponded to no preference, and scores closer to the minimum value of -1 represented a preference for more rarely chosen stimuli.

$$Reward\;Pairs\;choice\;score=\frac{\sum_{pairrew}p(choose|pairrew,frequent)-p(choose|pairrew,rare)}{3}$$

Similar to the choice data, we computed an individual score of frequency-based habit-related RT acceleration (*Reward Pairs RT score*), corresponding to the decrease in median RTs when choosing a previously frequently chosen stimulus relative to a previously less frequently chosen stimulus, for each same-reward pair. *Reward Pairs RT scores* closer to the maximum value of 0.8 represented relative RT acceleration for stimuli that participants chose more frequently in training, scores around zero corresponded to little effect of choice frequency during training, and scores closer to the minimum value of -0.8 represented faster responses for stimuli that participants chose more rarely in training.

$$Reward\;Pairs\;RT\;score=\frac{\sum_{pairrew}Mdn(RT|pairrew,rare)-Mdn(RT|pairrew,frequent)}{3}$$

We computed an individual score of frequency-based rating changes (*Reward Pairs rating score*) corresponding to the average increase in liking ratings for previously more frequently chosen stimuli relative to previously less frequently chosen stimuli. *Reward Pairs rating scores* closer to the maximum value of 100 represented stronger increases in liking ratings for previously more frequently chosen stimuli, scores around zero signified no differential rating changes between more and less frequently chosen stimuli, and scores closer to the minimum value of -100 represented greater increases in ratings for previously more rarely chosen stimuli.

$$Reward\;Pairs\;rating\;score=\frac{\sum_{pairrew}\Delta rating(post-pre|pairrew,frequent)-\Delta rating(post-pre|pairrew,rare)}{3}$$

In addition to these three measures and the learning rate parameter of the choice kernel in the computational model, we calculated the magnitude of the effect of training for the choices and RTs. To do so, we calculated the Reward Pairs choice score and Reward Pairs RT score for the first test phase on Day 1 and subtracted this from the respective scores of the test phase on the last day and divided the result by two. Therefore, there were six scores of participants’ behavior potentially reflecting habitization, which entered the factor analyses and structural equation models of Research Questions 3 and 4.

#### Unrewarded Habit Task

This task studied the effect of differences in behavioral frequency in the absence of external reinforcement during training on unrewarded free choice during test. At the beginning of each training trial of this instructed-choice paradigm (Figure 1C), two stimuli appeared on the screen. After a uniformly jittered interval of 150-300 ms, one of the stimuli received a blue frame indicating that participants should select this stimulus as quickly and accurately as possible by pressing either “F” or “J” on the keyboard. Participants had 500 ms to respond. Time pressure supposedly facilitates habit development (Luque et al., 2020). If they pressed a button before the onset of the blue frame, the phrase “Pushed too early” appeared for 1300 ms, no further response in this trial was recorded, and the trial counted as missing. If participants failed to respond within 500 ms, the words “Too slow” appeared for 800 ms and the trial counted as missing. If participants responded within the given time window, a brown frame appeared around both the chosen and unchosen stimulus for 800 ms. Thus, all stimuli were presented with the same duration and participants received feedback that their response was recorded, but the feedback was the same for either action, limiting outcome-related learning to a minimum. The ITI was exponentially jittered between 1000 and 3000 ms and included also the remainder of the response time window.

There were two stimulus pairs in the Unrewarded Habit task, which differed in the instructed choice proportions. In one pair, one stimulus was indicated to be chosen in 80% of the training trials, the other stimulus in the remaining 20% of training trials. In the other pair, the instructed choice proportion of both stimuli was 50%. Stimuli were shown left and right of the center of the screen. To reduce cognitive demand and maximize the stability of the task environment, the presentation location of each stimulus remained fixed within but varied across participants. Participants completed one training session on each day. Each training session consisted of six blocks of 20 trials for each of the two stimulus pairs, amounting to 600 training trials per pair.

The test phase (Figure 1D) followed immediately after the fifth training session. During the test phase, we first presented ten trials of each of the two trained stimulus pairs in the same presentation locations as during training. Then each of the four stimuli was presented with each other stimulus four times with balanced presentation locations of stimuli (Supplementary Information section 3.2.1 for analysis of the effect of same vs. reversed location of stimuli in test and training). These trials were randomly interspersed with an additional ten trials of each of the trained stimulus pairs where stimuli appeared in switched locations compared to training. Starting with the same stimulus pairs and presentation locations aimed at postponing the cognitive conflict participants might experience when stimuli were paired with other stimuli than during training or when locations were switched, which might in turn lead them to invest cognitive control masking habitual responding. The instructions informed participants to choose one of the two presented stimuli intuitively. Neither should they think much about the choice nor should they decide which button to press before the stimuli appear. They had to decide within a response time window of 500 ms to limit the elaboration of decision strategies and facilitate the expression of potential habitual responses. With no obvious values guiding choice, we could expect random choices or simple choice strategies like alternating response sides irrespective of the presented stimuli. Alternatively, participants could have developed a preference for previously more frequently chosen stimuli over less frequently chosen stimuli, which would reflect the development of a habit induced by choice frequency. Similar to the procedure in the Reward Pairs task, participants performed the same test phase before the very first training session as simple check for pre-existing sensory preferences and to allow investigating training-induced changes in choices.

The test phase was followed by a second stimulus rating. The outcome measures of interest were the observed choice frequencies and RTs during the test phase and the change of liking of the stimuli from the rating before training to the rating after the test phase as a function of the previous choice frequency during training. A frequency-based habit would decrease RTs and increase preferences and liking ratings for more frequently chosen stimuli.

Similar to the Reward Pairs task, we computed an individual score of frequency-based habitual choices during test (*Unrewarded Habit choice score*) capturing the relative preference for the more frequently chosen stimulus during training over the less frequently chosen stimulus. Positive *Unrewarded Habit choice scores* corresponded to a preference for the previously more frequently chosen stimulus, scores around zero reflected no preference, and negative scores represented a preference for the less frequently chosen stimulus.

UnrewardedHabitchoicescrore=p(choose|frequent)−p(choose|rare)

In addition, we computed an individual score of frequency-based habit-related RT acceleration (*Unrewarded Habit RT scores*) corresponding to a decrease in median RTs when choosing the previously more frequently chosen stimulus relative to the previously less frequently chosen stimulus. More positive *Unrewarded Habit RT scores* represented faster responses for the previously more frequently chosen stimulus, scores around zero corresponded to little effect of choice frequency during training, and more negative scores reflected faster responses for the previously more rarely chosen stimulus.

UnrewardedHabitRTscrore=Mdn(RT|rare)−Mdn(RT|frequent)

Finally, we computed an individual score of frequency-based habit-related rating changes (*Unrewarded Habit rating score*) capturing the increase in the liking rating of the previously more frequently chosen stimulus relative to the less frequently chosen stimulus. More positive *Unrewarded Habit rating scores* represented stronger increases in liking ratings for the previously more frequently chosen stimulus, scores around zero corresponded to no differential rating changes between more and less frequently chosen stimuli, and more negative scores reflected stronger increases in ratings for the previously more rarely chosen stimulus.

UnrewardedHabitratingscrore=Δrating(post−pre|frequent)−Δrating(post−pre|rare)

As with the Reward Pairs task, we computed the magnitude of the effect of training for choices and RTs and used them in the analyses of Research Questions 3 and 4.

#### Outcome Devaluation Task_Tricomi_

As a benchmark paradigm of habit induction, the first outcome devaluation task (Tricomi et al., 2009) used actual snack foods as outcomes and a specific satiation procedure for devaluation (for a multi-lab replication study: (Pool et al., 2021); for the MATLAB code: https://perma.cc/E323-BK9F) We closely followed the procedure described by Pool and colleagues (2021). In this paradigm, participants first rated their level of hunger on a 10-point Likert scale from “very full” (1) to “very hungry” (10), then tasted and rated the pleasantness of six different snacks (three salty and three savory) on an 11-point Likert scale from “very unpleasant” (-5) to “very pleasant” (5). The favorite snack in each of the two categories was selected as outcomes in the task. A free-operant training phase followed, consisting of two sessions. This amount of training was equivalent to the moderate training group of Pool and colleagues (2021), who have shown no statistically significant difference in devaluation sensitivity between a participant group with two sessions in one day and a group with twelve training sessions equally distributed over three consecutive days. They concluded that most participants developed habitual responding within two training sessions and found no evidence to support the hypothesis that longer training makes participants more habitual in this task. Thus, we included this task with two training sessions only on the first day of the study.

Each of the two free-operant training sessions consisted of twelve task and eight rest blocks. One of three fractal images and a schematic indicating the response button appeared on the screen for the whole duration of a block (20 or 40 s). Participants could press the response button self-paced. If they pressed the button, they might receive a reward (either the sweet or savory snack, which was presented on the screen for 1000 ms) or nothing, in which case a grey circle was presented for 50 ms on the screen. Reward delivery followed a variable-interval schedule with a mean of 10 s (VI-10 s). The VI-10 s was implemented such that in each second there was a 10% chance that a button press within this second would deliver a reward. Thus, a reward became available on average every ten seconds and delivery was triggered by the first response after it became available. Each of the three fractals was associated with one of the two selected snacks or the rest block, in which participants were instructed not to respond. The order of blocks was pseudo-randomized within each participant so that no block type (sweet snack, savory snack, rest) occurred more than twice in a row.

After the second training session on the first day of assessment, one of the snacks was devalued by satiation (sweet or savory, balanced across participants). To induce satiation, the participants were presented with a bowl of the respective snack, which had been weighed beforehand. Participants could then eat as much as they wanted (and the amount served as a control variable). Next, participants again rated their level of hunger and the pleasantness of the two snacks used in the training sessions.

The devaluation procedure was followed by the test phase, which consisted of six task blocks (three for each of the two snacks) and three rest blocks, presented in pseudo-randomized order. Exactly as in the training sessions, the previously trained fractals and response schematic were presented but no reward was delivered. However, participants were instructed that they were nevertheless accumulating the respective snack outcomes associated with the presented fractal. Each block had a duration of 20 s. Each response was followed by presentation of a grey circle for 50 ms, but without food (i.e., in extinction).

To compute the behavioral measure of interest for this task, we calculated the average number of button presses per second in the second training session (i.e., six blocks per snack, *pre devaluation*) and in the test phase (i.e., three blocks per snack; *post devaluation*). If participants did not adapt their level of responding in the test session compared to the training for the devalued snack outcome, behavior was defined to be habitual for this task. Thus, we calculated a “behavioral adaptation index” (BAI; Pool et al., 2021) by determining the change in button presses per second (bp/s) before vs. after devaluation for the valued snack and subtracting it from the change for the devalued snack:

$$\begin{array}{cc} BAI= & \left[m\left(\frac{bp}{s}|valued,\;post\;deval.\right)-m\left(\frac{bp}{s}|valued,\;pre\;deval.\right)\right]\\ & -\left[m\left(\frac{bp}{s}|devalued,\;post\;deval.\right)-m\left(\frac{bp}{s}|devalued,\;pre\;deval.\right)\right] \end{array}$$

We related this index to behavior in the other tasks (RQ3) and to real-life habitual behavior (RQ4).

#### Outcome Devaluation Task_Luque_

A second previously published outcome devaluation task (Luque et al., 2017, 2020) provided another benchmark measure of habit. This paradigm (available at https://perma.cc/UY4C-ZAJF) operationalized habits as RT switch costs without affecting choice (Luque et al., 2020). Participants learned to associate different visual stimuli and button presses with different outcomes. The task included four types of stimuli in the form of cookies, two responses in the form of selecting one of two aliens, and three outcomes in the form of diamonds. In each trial, one of the cookies appeared and participants gave it to one of the two aliens by button presses in order to receive one of three diamonds, which were worth five, ten, or 100 points, respectively. Aliens differed in the type of diamond they traded for the different cookies. Thus, participants learned which cookie-alien combination (i.e., stimulus-response association) led to the best outcomes. Interleaved consumption trials, in which participants chose between different diamonds, tested whether participants had learned the different outcome values.

Participants performed eight training blocks with 52 trials each (12 trials with each of four cookie stimuli and four consumption trials) each day on four consecutive days starting on the second day of the study (Table S1). In test phases on the first and last training day, specific outcome types were devalued. Specifically, at the beginning of each test block, one type of diamond was announced to yield no points in this block and participants had to react by switching their responses to a diamond that had been worth fewer points during training but was now the only valuable outcome. These response switches supposedly led to a conflict between perseverative habitual and flexible goal-directed behavioral control. Such conflict was not necessarily evident in the choices (even though the animal literature clearly shows that habitization affects choices; Balleine & O’Doherty, 2010) but associated with an increase in RTs in switch trials (Luque et al., 2020). Hence, the RT switch cost was the outcome measure of interest in this task. As an increase in habit strength occurred only under time pressure in previous studies, we used the time-constrained task versions of Experiments 2a and 2b of Luque and colleagues (2020) and expanded the training from three to four days to increase the probability of inducing habits not only in RT but also in choice.

For the analyses regarding RQs 3 and 4, we calculated two scores similar to the original study (Luque et al., 2020). First, the difference of the proportion of responses leading to the devalued outcome in the test phase minus the same responses during the last training block before devaluation separately for the high and low valued outcome.

DVALchoicescore=p(choose|devaluedstimulus,testphase)−p(choose|devaluedstimulus,trainingphase)

This resulted in the *DVAL choice score*, which ranged from -1 to 1, with -1 reflecting perfect goal-directed switching after devaluation, 0 corresponding to habitual perseveration of the now-devalued response, and (the very unlikely score of) 1 indicating that participants did not choose the valued outcome during training but did so after devaluation. We included the choice score to test whether additional training facilitated habitual behavior not only with regard to RT but also choice in this task.

Second, the *DVAL RT switch costs* in test trials were calculated as the median RTs in switch trials after devaluation (separately for previously high and low valued outcome trials) minus the median RTs in the respective trials in the last training block before devaluation. Switch trials referred to the trials in which participants successfully switched away from the devalued to the non-devalued stimulus during the test phase. RTs in switch trials were contrasted with the RTs for choosing the same stimulus before its devaluation. For example, in trials comparing a stimulus worth five points to a stimulus worth 100 points, the median RT of choosing the 100-point stimulus before devaluation was subtracted from the median RT of switching to the five-point stimulus after devaluating the 100-point stimulus.

DVALRTswitchcost=Mdn(RT|switchtonon−devaluedstimulus,testphase)−Mdn(RT|tobedevaluedstimulus,trainingphase)

The *DVAL RT switch costs* were stronger if responses to devalued stimuli were slower during test than training. These costs presumably arose from the cognitive conflict of having to overcome habitual choice tendencies and switch to the previously less valued choice.

As with the Reward Pairs and Unrewarded Habit tasks, we computed the magnitude of the effect of training for choices and RT switch costs to use them in the analyses of Research Questions 3 and 4.

#### Contingency Degradation Task

We used a modified version of the contingency degradation task designed by Vaghi and colleagues (2019) to capture the second criterion of the traditional habit definition. This free-operant task was presented in several 120 s blocks. In each block, participants viewed a white triangle on the screen and were free to press the response key as often as they wished. Unbeknownst to them, each 1 s-bin was associated with a probability of monetary reward if the participant responded and a probability of reward if the participant did not respond during the bin. The contingency, i.e., the relation between the two probabilities varied between blocks but stayed constant within each block. At the end of each block, participants were asked to rate on a visual analogue scale how causal they thought their responses were for the reward to materialize. The scale ranged from -100 to 100 indicating the continuum from “Responses always prevented rewards” to “Responses always caused rewards”.

In blocks with positive contingency, the probability of receiving a reward in each 1 s-bin if at least one response had been made (P(O|A)) was positive (e.g., constantly 60%), while the probability of receiving a reward without having made a response (P(O|~A)) was zero. In negative contingency blocks, the probability of receiving a reward upon having produced a response was zero and the probability of receiving it without having made a response was positive. In blocks with degraded contingency, both the probability of receiving reward contingent on having produced a response and the probability of reward in the absence of a response were positive (but not necessarily of the same magnitude), that is, rewards could be caused by a response but might have also appeared without it. Behavior was deemed habitual in this task if participants with prior training on the positive contingency schedule did not adapt their response rates in blocks with degraded or negative contingency.

We used a modified version of the task that included blocks with positive contingency of P(O|A)=0.6 and P(O|A)=0.3 (both block types having P(O|~A)=0; Table S3 for an overview of all block types and how often they were presented on each assessment day). The positive contingencies were varied between blocks to keep participants engaged in the task. In addition, blocks with degraded contingencies were interspersed. Training on this task was done on each of four consecutive days from the second day of the study (Table S1).

The outcome measure of interest in this task was a ratio score of the number of responses in the (fully and partially) degraded blocks relative to the sum of responses in both the (fully and partially) degraded and contingent (non-degraded) blocks of the last day (Ersche et al., 2021; Vaghi et al., 2019).

$$DGRD\;ratio\;score=m(\frac{button\;presses}{s}|\deg raded\;blocks)/[m(\frac{button\;presses}{s}|degraded\;blocks)+m(\frac{button\;presses}{s}|non-degraded\;blocks)]$$

Thus, this variable was close to 0.5 if participants responded to a similar degree in both contingent and degraded blocks, reflecting habitual behavior, i.e., an insensitivity to changes in contingencies. The score was close to zero the more participants responded during the contingent blocks and the less they responded during degraded blocks, that is, if participants were highly sensitive to changes in action-outcome contingencies. Values between 0.5 and one corresponded to more responses during degraded than contingent blocks, which may reflect misunderstanding of task instructions or lack of attention or motivation. In addition, the causal judgements at the end of each block were used as an outcome measure of interest.

We further computed the magnitude of the effect of training for the ratio score as with the previous tasks by subtracting the score of the first day of training from the score of the last day to use them in the analyses of Research Questions 3 and 4.

#### Sequential Markov Decision Task (2-Step)

The sequential Markov decision task (Kool et al., 2016) used model-free (as opposed to model-based) reinforcement learning to operationalize habitual (as opposed to goal-directed) behavioral control. This task version is freely available (https://perma.cc/56SV-DA2Y) and takes into account several limitations (Akam et al., 2015; Kool et al., 2016, 2017) of the original task (Daw et al., 2011).

Participants started each trial in one of two possible states (Figure S4). Each of these two initial states had the same representation of planet earth in the background but contained two different stimuli in the form of spaceships (i.e., spaceships A vs B in one initial state and spaceships C vs D in the other). Participants needed to choose one of the two spaceships. This choice led to a second state, in which participants faced one of two aliens. The second state did not require a choice but only a press of the space bar in response to seeing the alien, which yielded space treasure (if positive), antimatter (if negative), or nothing. These outcomes, representing points, varied according to a Gaussian random walk with a mean of zero points, a standard deviation of two points, and reflecting boundaries at the minimum of minus four and maximum of five points. Crucially, in each of the two initial states, one spaceship deterministically flew to one alien and the other spaceship to the other alien (i.e., spaceships A and C flew to alien 1 and spaceships B and D to alien 2). Thus, the two states were essentially equivalent. This feature was important to dissociate model-free from model-based control. Model-based control “knew” about the equivalence because it represents the full state space including the transitions between states and can use the outcome in a specific second state to make inferences about the other initial state. In contrast, model-free control had no state representation and could guide choice only by the value of outcomes. Hence, model-free and model-based control made qualitatively different predictions about decisions when the initial state of the trial differed from the one on the previous trial.

Analysis of 2-Step data was based on the computational model provided by Wouter Kool (https://perma.cc/56SV-DA2Y) and the mfit toolbox for Matlab provided by Sam Gershman (https://perma.cc/S2J8-MUVE). This model combined model-free and model-based reinforcement learning. The behavioral data of each participant was fit to this hybrid model, yielding an individual best-fitting estimate of all free parameters (*α, β, λ, π, ρ, ω*; Supplementary Information section 2.3 for details). For the present study, the *ω* parameter was the outcome measure of interest as it captures the relative weight of model-based and model-free reinforcement learning in computing the subjective value of options to guide behavior. For our analyses, we inverted this parameter so that larger values (closer to the maximum of 1) corresponded to more model-free (i.e., presumed habitual) control and smaller values (closer to the minimum of 0) to more model-based control.

#### Working Memory Capacity Tasks

We used three tasks of a working memory test battery (Lewandowsky et al., 2010) to control for the effects of cognitive abilities in our analyses of habitual behavior: numeric memory updating (NMU), spatial short-term memory (SSTM), and sentence span (SS). By using three different tasks assessing the two processes entailed in working memory (storage in the context of processing and relational integration) with two different content types (verbal, including numeric, and spatial) we aimed to achieve a more construct-representative measure of working memory capacity. These three tasks have been shown to correlate highly with a construct-representative measure of working memory capacity (Lewandowsky et al., 2010) and, thus, can be considered as working memory marker tasks.

In the NMU task, participants had to memorize numbers and update them according to mathematical operations. Each trial started with the presentation of a set of three, four or five frames. A number from one to nine then appeared in each frame, one by one, for one second. Next, arithmetic operations ranging from -7 to +7, excluding 0, could appear randomly in any frame. Participants had to memorize the result of the operation for each frame. After two to six updating operations, the trial ended, a question mark appeared consecutively in each frame and participants had to type in the respective current digit for that particular frame. The outcome measure was the participant-specific total proportion of items recalled correctly, ranging from 0 to 1.

In the SSTM, participants had to memorize the position of several dots in a 10x10 grid. In each trial, two to six dots appeared one by one for 900 ms each with 100 ms interstimulus interval. After presentation of the last dot, a cue prompted the participants to reproduce the relative pattern of dots, that is, the location relative to each other. Neither the exact absolute position of the individual dots in the grid nor the sequence of the dots mattered. Participants received 2 points for an exact match, one point for recalling a dot with a deviation of one cell and zero points for higher deviations. The participant-specific total sum of points was divided by the maximum possible score to yield a proportion of correct answers ranging from 0 to 1 similar to the NMU task (see Lewandowsky et al., 2010, p. 573f).

The SS task was a complex-span paradigm comprising a memory component and a secondary processing task (Lewandowsky et al., 2010). The present version of the task used verbal material for both components. For the secondary processing component, participants read a sentence consisting of three to six words for 5 s and had to judge and indicate within this time window whether this sentence was meaningful or not. Examples for a meaningful and meaningless sentence are “All bumblebees can fly.” and “All birds live in the city.”. After the meaningfulness response, a consonant was presented on the screen for 1 s before a 100 ms inter-trial interval and the beginning of the next trial. Participants had to memorize the consonants shown in the order they have been presented and report them back after the end of each list. Lists had a length of three to seven sentences and consonants. The task began with three practice trials containing two, three, and four sentences and consonants, respectively. The outcome measure was the participant-specific total proportion of correctly recalled consonants (including the correct position in the list) ranging from 0 to 1 similar to the other working memory capacity tasks. Participants were instructed to categorize the meaningfulness of the sentences and report the memorized consonants as accurately as possible. Thus, they were not aware that only the correct reporting of consonants was used as a measure of their working memory capacity.

### Questionnaires

All questionnaires were performed by the participants online before the first training session. They were programmed so as not to produce missing responses, that is, participants were not allowed to proceed to the next page if an item had not been answered.

#### Self-Report Habit Index (SRHI)

The Self-Report Habit Index (SRHI; Thurn, 2014; Thurn et al., 2014; Verplanken & Orbell, 2003) is a self-report questionnaire used to measure the habit strength of real-life behaviors. These behaviors were freely definable (e.g., “Taking the car to go to work is something …” or “Drinking alcohol is something …”) and participants completed twelve items (e.g., “I do frequently”, “that makes me feel weird if I do not do it”, or “I would find hard not to do”) for each of them. We assessed the habit strength of eleven different real-life behaviors: brushing teeth, eating meat, having breakfast, drinking coffee, drinking alcohol, smoking, going to school/university/work by bike, going to school/university/work by bus/tram/train, doing sport/exercises, eating snacks/candy, and watching TV/online streaming services (items partly taken from Gardner et al., 2011, 2012; Rees et al., 2018). In line with previous research, we used a 7-point Likert scale (i.e., from 0=“disagree” to 6=“agree”). The response for each item was recoded so that higher numbers corresponded to stronger habits. All twelve responses regarding a particular behavior were summed up and divided by the maximum possible points (i.e., 72) to obtain a value between zero (lowest possible habit strength) and one (highest possible habit strength). These maximally eleven behavior-specific scores were then averaged to a final habit strength score. Because we focused on habits as frequently expressed behavioral routines, we considered only those of the eleven behavior-specific scores for the calculation of the final mean habit score for which participants had indicated that they show the behavior frequently (i.e., response category three or higher in response to the item “I do frequently”). If not at least three of the eleven behaviors were performed frequently, then the final habit strength score was coded as missing to ensure that the final score represented general habitual behavior.

#### Creature of Habit Scale (COHS)

The Creature of Habit Scale (COHS; Ersche et al., 2017; Overmeyer et al., 2020) consists of 27 items comprising a statement about behavioral routines, automatic responses, and habits. Answers were given on a 5-point Likert scale ranging from 1=“strongly disagree” to 5=“strongly agree”. Example items are “I like to park my car or bike always in the same place”, “I tend to do things in the same order every morning (e.g., get up, go to the toilet, have a coffee…)”, and “I tend to like routine”. The final scores for the two distinct subscales of automaticity (11 items) and routine (16 items) were calculated by averaging the respective item scores. Correlation between these subscales is rather low (r=.154; Ersche et al., 2017). Therefore, they were not combined to an overall score but used as separate measures of habitual behavior.

#### Habitual Tendencies Questionnaires (HTQ)

The Habitual Tendencies Questionnaire (HTQ; Ramakrishnan et al., 2021) is an eleven-item self-report questionnaire assessing the subscales Compulsivity, Regularity, and Aversion to Novelty. Example items are “The same thoughts often keep going through my mind over and over again”, “There is comfort in regularity”, and “I look forward to new experiences” (reverse scored). Answers were given on a 7-point Likert scale ranging from 0=“strongly disagree” to 6=“strongly agree”. The final score in this questionnaire was the result of summing up the values of all eleven items. In addition, the scores of the three subscales were formed by summing up the four or three items belonging to each scale, respectively. Both the total score and the subscale scores were used for further analyses.

#### Trier Inventory for Chronic Stress (TICS)

As previous research has indicated a link between habitual behavior and some measures of stress and anxiety (Friedel, 2017; Pool et al., 2021; Wirz et al., 2018), we assessed chronic stress, trait anxiety, and impulsiveness with established questionnaires and explored the associations between these measures and measures of habitual control. However, this was only a side issue for our study to allow future research to examine the relationships between stress and habit formation more closely.

The Trier Inventory for Chronic Stress (TICS; Petrowski et al., 2012; Schulz et al., 2004) is a self-report questionnaire containing 57 items, which assesses chronic stress on nine factors: work overload, social overload, pressure to perform, work discontent, excessive demands at work, lack of social recognition, social tension, social isolation, and chronic worrying. Example items are “I have too many tasks to perform.”, “Although I try, I do not fulfill my duties as I should.”, and “I have unnecessary conflicts with others.” Responses were given to the question, how often participants have experienced an item or felt that way in the last three months, on a 5-point Likert scale with verbal anchors for each point (0 = “never”, 1 = “rarely”, 2 = “sometimes”, 3 = “often”, 4 = “very often”). Based on the item-factor mapping reported by Petrowski and colleagues (2012), the raw item scores of each scale were summed up to calculate the nine scale scores for further analyses.

#### State/Trait Anxiety Inventory (STAI)

We implemented the trait version of the State/Trait Anxiety Inventory (STAI; Spielberger et al., 1999). This version of the STAI consists of 20 items assessing general feelings of anxiousness, worry, and sadness. Example items are “I feel nervous and restless”, “I feel like a failure”, and “I feel inadequate”. Responses to the items are given on a 4-point Likert scale with verbal anchors for each point (1 = “almost never”, 2 = “sometimes”, 3 = “often”, 4 = “almost always”). Raw item scores were summed up to generate a trait anxiety score for further analyses.

#### Barratt Impulsiveness Scale (BIS-15)

The Barratt Impulsiveness Scale short form (BIS-15; Meule et al., 2011; Spinella, 2007) is a 15-item self-report questionnaire used to measure impulsive behavior. Answers were given on a 4-point Likert scale with verbal anchors (1 = “rarely/never”, 2 = “occasionally”, 3 = “often”, 4 = “almost always/always”). Example items are “I act on impulse”, “I say things without thinking”, and “I plan tasks carefully”. The items were grouped into three subscales – motor, non-planning, and attentional impulsiveness – comprising five items each and a sum score of the raw item values for each subscale was calculated for further analyses.

#### Edinburgh Handedness Inventory (EHI)

The Edinburgh Handedness Inventory (EHI; Oldfield, 1971) is a questionnaire giving participants eight behavioral routines (e.g., brushing teeth, writing with a pen, or using scissors) and asking them which hand they prefer to use for performing these tasks. The answers were indicated on a 5-point Likert scale with verbal anchors of “always left”, “usually left”, “both equally”, “usually right”, and “always right”. Answers were coded on an integer scale from 0 (corresponding to “always left”) to 4 (corresponding to “always right”). Thus, sum scores close to the maximum (i.e., 32) represented strong right-handedness, while sum scores close to zero represented strong left-handedness.

#### Social Desirability Scale (SDS-17)

Social desirability is an answer tendency, which might influence choices during the test phase of the Unrewarded Habit task. In the test phase of this task, there was no obvious stimulus feature guiding choices, because the stimuli were not associated with reward and no information told participants what to choose. Therefore, we could have expected random choices or simple choice strategies like always choosing the left stimulus or alternating between stimuli in a pattern. Alternatively, participants could have deduced for the test phase that the experimenters expected them to prefer the stimulus previously instructed to be selected more often. If a participant was likely to respond in a socially desirable manner, they might have preferred this stimulus to supposedly satisfy the experimenter. For this reason, we included the Social Desirability Scale (SDS-17; Stöber, 1999, 2001) in our assessment. This is a 16-item self-report questionnaire to measure social desirability in general. Answers were forced binary decisions between “yes” or “no”. Example items are “I always stay friendly and courteous with other people, even when I am stressed out”, “I occasionally speak badly of others behind their back”, and “I take out my bad moods on others now and then”. A mean score of all items with “yes” responses coded as 1 and “no” responses as 0 (and reversed for six items) was the outcome measure of this questionnaire. Higher scores indicated a stronger tendency to answer in a socially desirable manner. If this mean score showed a statistically significant correlation with the choices in the Unrewarded Habits task, we would use it as a covariate in the analyses of hypothesis H2a to control for tendencies to answer in a socially desirable manner.

#### Sociodemographic Questionnaire

Participant age, gender, education level, socioeconomic status, and ethnicity was assessed to characterize the sample.

### Sampling Plan

There were no previously published studies that examined related research questions, leaving us with no base for an estimation of the expected effect sizes and of the required sample size. Furthermore, the generalized linear mixed effects models (GLMMs) used to analyze the pilot data did not converge with the full random effects structure, because of the insufficient sample size, preventing us from using these analyses as basis for our power analyses. Therefore, we simulated data with an assumed effect size that would be of interest (Cohen’s *d*=0.2 as a typical effect according to Gignac & Szodorai, 2016) and calculated an appropriate sample size to achieve 95% power for this effect size given our analytical approach. We simulated data based on the GLMMs used for the pilot data with the simr package (version 1.0.5; Green & MacLeod, 2016) in R (R Core Team, 2019). We added simulated participants to our pilot sample to reach a sample size of 200, then used subsamples of different size to calculate the observed power with the given sample size, resulting in a power curve for one fixed effect of the GLMM over a range of sample sizes. These analyses showed the strength of our mixed-effects within-subject design: we needed relatively small sample sizes to achieve adequate statistical power, because we model individual choices and RTs on a trial-by-trial level using more information than when using aggregated measures of behavior. For example, the simulation approach yielded a minimum sample size of 20 to achieve 98.20% (CI: 97.17%, 98.93%) power for the small effect of previous choice frequency in the Reward Pairs task. The same procedure for the GLMM of the choice frequencies during the test phase of the Unrewarded Habit task with a small effect of previous choice frequency yielded a minimum sample size of 55 to achieve 96.10% (CI: 94.71%, 97.21%) power for this effect.

In the contingency degradation task, habitual behavior corresponds classically to the absence of an effect of the degradation on response rates. Thus, there was no effect size for this effect to base a power calculation on. However, Vaghi and colleagues (2019) found the hypothesized stronger habitual behavior in patients with obsessive-compulsive disorder compared to control participants, with Cohen’s *d*=0.912. If we were to replicate their effect, we would need a sample size of 66 to reach 95% power (t test, independent groups, *α*=.05, two-tailed; G*Power 3.1.9.2; Faul et al., 2009). A similar issue held for the outcome devaluation task_Luque_, in which the absence of an effect of the devaluation indicates habitual choices. Following the rationale for the contingency degradation task, if we were to replicate Luque and colleagues’ (2019) effect (Cohen’s *d*=0.789) of the amount of training on responding with 95% power, we would require a sample of 86 participants (t test, independent groups, *α*=.05, two-tailed; G*Power 3.1.9.2; Faul et al., 2009). Thus, a bigger concern for the required sample size was the correlational approach used in Research Questions 3 and 4.

Regarding the correlational analyses between different tasks measuring habitual behavior (Research Questions 3), we considered a correlation of *r*≥.5 and a well-fitting unidimensional measurement model as the criterion for a common habit process involved in all six tasks. Our reasoning was based on previous findings with regard to typical associations of different cognitive and behavioral tasks in individual differences research (e.g., Kretzschmar et al., 2016) as well as research on habitual behavior (Friedel et al., 2014; Gillan et al., 2015; Sjoerds et al., 2016). As there were no studies reporting correlations between self-reported habits in questionnaires and experimental data (Research Question 4), we grounded our expectation of such a relation in previous research on the associations between test performance in cognitive tasks and the self-estimate of these cognitive abilities (*r*=.34-.55; von Stumm, 2014), and relations between SRHI scores and real-life behavior in a meta-analysis (*r*=.46; Gardner et al., 2011). However, in order to have a more conservative estimate of the required sample size, we decreased the expected size of the correlation to *r*=.3 (corresponding to a large effect according to Gignac & Szodorai, 2016) between experimental and real-life habitual behavior. Thus, to achieve 95% power, we required a sample size between 190 (RQ3) and 200 (RQ4) participants based on Monte Carlos simulations and the theorized measurement und structural equation models (Muthén & Muthén, 2002; Table 1).

To account for possible dropout during the successive training sessions, we increased the necessary sample size to 220 participants to be able to compensate up to 10% dropout (pilot study: 5.6%). As we used a planned missing design (see the Design section), 110 participants would enter the analyses of outcome devaluation task_Luque_ and the contingency degradation task, respectively. For the analyses across tasks (Research Questions 3 and 4), we used multiple imputation of the missing data of each participant (van Buuren, 2018). Hence, the sample size for these analyses would be 220. In addition, this sample size gave us sufficient statistical power (i.e., >99%) to identify a small effect (Cohen’s *d*=0.2) in all (G)LMMs used in RQs 1 and 2.

Participants were sampled from the database of the Zurich Center for Neuroeconomics and the Social and Neural Systems Laboratory. The behavioral lab allowed for the testing of 36 participants simultaneously. Exclusion criteria were not having performed the online questionnaires and 2-Step at the beginning of the first training session. To be included in the study, participants must not have partaken in the pilot study, be capable of understanding the German language for task instructions, and have normal or corrected-to-normal vision. Furthermore, we only included participants aged 18 to 40 years to avoid age effects and excluded participants with prior diagnosis of neurological or psychiatric disorders.

### Analysis Plan

For the analyses of RQs 1 and 2, we used (G)LMMs (Baayen et al., 2008; Bates et al., 2015). These mixed-effects models have the advantage of enabling the combination of continuous and categorical responses and allowing to model heteroskedasticity (Baayen et al., 2008). Furthermore, these analyses aimed to increase the precision of estimated group (fixed) effects by explicitly modelling the variance of the dependent variable associated with specific participants or items (random effects).

All (G)LMMs were initially set up with the maximal random effects structure (Barr et al., 2013). That is, each fixed effect (main effects and interactions) was included as a random effect with random intercept and slope nested in individual participants. In addition, the stimulus identity (i.e., the specific stimulus irrespective of the condition in which it was used) was included as a random effect (intercept only) controlling for the used subset of the 15 stimuli rated at the beginning of the study. In case of convergence failures or singular model solutions with all appropriate optimizers allowed by the *lmercontrol* function (package lme4, version1.1-21; Bates et al., 2015), the random effects structure was iteratively reduced starting with the random effect accounting for the smallest share of variance (Baayen et al., 2008; Barr et al., 2013). For this and all following tasks, analyses of binary outcome measures (i.e., choice in Reward Pairs and Unrewarded Habit tasks) were conducted as binomial GLMMs. Where the dependent variable was continuous (i.e., ratings, log-transformed RTs), we fit an LMM. All (G)LMMs were bootstrapped to estimate confidence intervals for the regression coefficients and all predictor variables that were not categorical were scaled so that each variable had a mean of zero and a standard deviation of one to enable comparisons of the size of their respective regression coefficients.

The level of statistical significance for all frequentist analyses was set to α=.05, two-tailed, unless stated otherwise. All bootstrapped analyses were based on 5,000 draws.

### RQ1: Does a frequency-based habitization process exist?

#### Choices

For the Reward Pairs task data, we used a GLMM to analyze trial-wise left vs. right choice. Included predictors of choices were the difference in the values of the left and right stimuli (fixed effect, continuous) and the difference in previous choice frequency (fixed effect, continuous). We did not expect these effects to interact but explored their interaction because it may be relevant for future research on addiction. In analogy to value, we assumed that not the absolute choice frequency during training drove choice during test, but the relative frequency compared to the alternative option. We hypothesized that relative previous choice frequency predicted choice during test in addition to relative reward values. Such a predictive relation would support a frequency-based habit process.

#### Response times

An LMM was fit to RTs in the test phase. Specifically, log-transformed RTs were regressed on the value difference between the chosen and unchosen option (fixed effect, continuous), their difference in choice frequency during training (fixed effect, continuous), and the interaction of these effects. We used the difference between chosen and unchosen stimulus in RT analyses (in contrast to left vs. right in analyses of choices) to reduce the number of required regressors and simplify the analysis. If higher frequency of choice during training gave rise to stronger habits during test, then larger differences in choice frequency should be associated with faster responses as well. Hence, we hypothesized to find main effects of value and choice frequency difference, with faster responses for larger, positive differences. The main effect of previous choice frequency would again indicate frequency-based habitual behavior. In addition, we included the score of the EHI as a covariate (fixed effect, continuous) in this analysis to control for generally faster responses with the preferred hand.

#### Stimulus ratings

An additional LMM regressed the ratings of the stimuli used in the Reward Pairs task after the test phase on the ratings before the training (fixed effect, continuous), the reward levels associated with the stimuli (fixed effect, ordinal), the relative choice frequency during training (fixed effect, continuous), and the interactions between these effects. If choice repetition enhanced the subjective value of chosen stimuli, then post-study ratings should be higher for stimuli the participants chose more frequently during training than for stimuli they chose less frequently while controlling for pre-study ratings and reward value, that is, we hypothesized a statistically significant main effect of choice frequency during training.

#### Computational model parameters

We fit the choice data with computational models, aiming to obtain a more detailed understanding of the mechanisms that underlie choices. The computational model consisted of temporal difference reinforcement learning and a kernel capturing choice frequency (see Supplementary Information 2.1 for details about the computational models) thus combining value-based reinforcement learning and frequency-based habit learning. To investigate whether frequency-based learning had a significant role for choices during training and test sessions, we compared models including either re-inforcement learning, a choice kernel, or both (or random choices independent of all kinds of stimulus values) with regard to their fit to the data. We hypothesized that model comparison and selection over the whole group based on the exceedance probability (Daunizeau et al., 2014; Rigoux et al., 2014; Stephan et al., 2009) would favor a model including the choice kernel for the training and test choice data over models without a choice kernel, which would support the assumption that a frequency-based habit process contributes to behavioral control in our task. In addition, we explored model selection for individual participants based on the Bayesian Information Criterion (BIC; Schwarz, 1978). For this, we used the criteria described by Raftery (1995) interpreting BIC differences of 6-10 as strong, and >10 as very strong evidence for the model with the smaller BIC. Thus, we did not assume that all participants necessarily adopt the same strategies in performing the task but might have shown inter-individual differences. Moreover, we explored the fit of additional models (e.g., rate correlation theory; Perez & Dickinson, 2020).

Participants with more than 50% missing trials in any training or test session were excluded from these analyses. They were also excluded from the analyses of the Reward Pairs task if they chose the worse of two stimuli during one training or the test session in at least 50% of trials. In addition, trials with RTs less than 50 ms were discarded, because such fast responses could not be induced by perceived stimulus features.

### RQ2: Is external reinforcement of behavior necessary for frequency-based habitization or can the process arise also in a context without external reinforcement?

#### Choices

All choices made during the test phase were modelled with a GLMM. Specifically, left versus right choice was regressed on the relative frequency of making these choices during training (fixed effect, continuous) in a hierarchical binomial model. We hypothesized that relative choice frequency during training significantly predicts relative choice frequency in the test phase. Given that behavior in the Unrewarded Habit task was not reinforced, it may be more susceptible to researcher demand effects than the Reward Pairs task. To control for such effects, we would include the mean score of the Social Desirability Scale (SDS-17; Stöber, 1999, 2001; see Questionnaires) in the model as covariate of no interest (fixed effect, continuous) if the SDS-17 score would show a statistically significant correlation with choices. The covariate would be z-transformed (i.e., standardized) aiding convergence of the GLMM (Harrison et al., 2018) and making the corresponding regression coefficients interpretable as a standardized effect size (Schielzeth, 2010).

#### Response times

The log-transformed RTs in the test phase were analyzed in an LMM as a function of previous choice frequency and stimulus presentation location. Thus, RTs were regressed on the stimulus presentation location of the chosen stimulus in relation to training (fixed effect, dichotomous, same vs. reversed location) and the difference in previous choice frequencies of the chosen and unchosen option (fixed effect, continuous). If a frequency-based habit had emerged, participants should respond faster in trials with the same stimulus presentation locations as in the training sessions compared to switched locations when choosing the stimulus with a higher choice frequency. Thus, we hypothesized that we would observe an interaction between stimulus presentation location and previous choice frequency with fastest (slowest) RTs for presentations at the same (reversed) locations and high (low) previous choice frequency. Again, we included the EHI score (fixed effect, continuous) in this analysis to control for effects of handedness on RTs.

#### Stimulus ratings

Another LMM regressed the ratings of the used stimuli after the test session on the ratings before trainings (fixed effect, continuous) and the relative choice frequency during training (fixed effect, continuous). Again, we hypothesized that the relative choice frequency affects post-training ratings, with higher ratings of the stimulus chosen more frequently during training compared to those stimuli chosen less frequently, while controlling for pre-training ratings.

Participants were excluded from analyses with the Unrewarded Habits task if they failed to respond in more than 50% of trials in any training or test session or if they chose the wrong stimulus during one of the training sessions in 50% or more of the trials. In addition and similar to the Reward Pairs task, trials with RTs less than 50 ms were discarded.

### RQ3: Is there a universal (i.e., paradigm independent) habitization process?

To analyze associations between experimental paradigms assessing habitual behavior, we used the previously described scores of choices, RTs, stimulus ratings, and free parameters of computational models. The associations between the different paradigms were investigated based on bootstrapped Pearson correlations and their bootstrapped 95% confidence intervals. In case of more than two scores (i.e., choices and RTs, Table S2), we applied confirmatory factor analysis (CFA) to test whether a measurement model with one (Hypothesis 3.1) or two (Hypothesis 3.2) latent factors would fit the data. Model fit was evaluated according to the criteria proposed by Schermelleh-Engel and colleagues (2003). In detail, we investigated the χ^2^ goodness-of-fit statistic, Comparative Fit Index (CFI ⩾ .95), Root Mean Square Error of Approximation (RMSEA ⩽ .08), and Standardized Root Mean Square Residual (SRMR ⩽ .10). Comparison of different measurement models (Hypothesis 3.2) was based on Satorra-Bentler-scaled χ^2^ difference tests (i.e., non-significance indicated equal model fit).

For the analyses of Research Question 3 and the following Research Questions, we imputed missing data of the one task not performed by each participant (see Sampling plan) based on multivariate imputation by chained equations (van Buuren & Groothuis-Oudshoorn, 2011). The imputation procedure takes advantage of the correlational patterns in the dataset, estimating the values of missing data for each participant several times. Since the proportion of missing values in one variable was maximally 50%, 50 imputed data sets were created (White et al., 2011). The resulting statistical parameters of each data set (e.g., correlation coefficients) were pooled based on Rubin’s (1987) rules to answer the Research Question.

### RQ4: How does the habitization process relate to real-life habitual behavior?

Based on the measurement model investigated in Research Question 3, we examined the association between the latent habitization process and self-reported habitual behavior according to the COHS, SRHI, and HTQ questionnaires. In case of just two available scores (i.e., stimulus ratings; computational parameters, see Table S2), the measurement model would be empirically under-identified and, therefore, we examined the association based on bootstrapped Pearson correlations and their bootstrapped 95% confidence intervals for each paradigm. In case of conflicting results regarding the association with the three questionnaires, the association with the SRHI was regarded most valid.

### RQ5: Is there a difference in validity with regard to various behavioral measures of habitual behavior?

To evaluate the hypotheses of the previous four Research Questions, it was sufficient if the results for at least one behavioral measure provided evidence for an effect of frequency-based behavior for each Research Question. However, to evaluate the validity of the different behavioral measures, we examined the result pattern across all Research Questions with a focus on criterion validity (i.e., real-life habitual behavior; RQ4). If, for example, the results for RQ3 based on RTs and choices provided evidence for a universal habitization process but the results for RQ4 provided evidence for criterion validity only for RTs but not for choices, then RTs should be considered a more valid behavioral measure of habitual behavior than choices. As outlined above, we considered this Research Question as exploratory (and future research may want to revisit this question from a clinical perspective).

## Results

### Participants

257 participants showed up for the first on-site assessment, of which 26 dropped out over the course of the five days of assessment. Further 10 participants were excluded because of a prior diagnosis of neurological or psychiatric disorders and one because of being older than 40 years (see Sampling plan for ex-/inclusion criteria). Thus, the full sample consisted of 220 participants. For all analyses including data of the Reward Pairs or Unrewarded Habits task, there were additional a-priori defined exclusion criteria (see Analysis plan of RQs 1 and 2 and Supplementary Analyses) resulting in further exclusion of seven and six participants, respectively. One participant was excluded in both Reward Pairs and Unrewarded Habits tasks, the remaining exclusions concerned only one or the other task. For one additional participant, test data of the Unrewarded Habits task was lost due to a technical error. Therefore, the final sample size for analyses of RQ1 and RQ2 was 213; for RQs 3-5 it was 208.

Participants of the full sample (N=220) were on average 23.25 years old (SD=3.35, range 18-39 years). 109 participants reported a female gender, 110 male, and one diverse. 215 participants were currently living in Switzerland, two in Germany, one in Austria, and two did not specify their current residency. For 156 participants (70.9%), German was their mother tongue. Of the remaining 62 participants, 13 reported elementary proficiency, 24 intermediate proficiency, and 27 advanced proficiency in German. No participant indicated having only basic knowledge of German. Asked for their educational level, one participant indicated still going to school, one had completed secondary school, 127 participants (57.7%) had a diploma that would allow them to attend a tertiary education institution, and 91 participants (41.4%) had a degree from a tertiary education institution (e.g., a university or a university of applied sciences). Asked for their current occupation (for which we allowed multiple responses), 206 participants (93.6%) indicated being a student, five participants were unemployed, six participants were in an apprenticeship, 26 participants were employees, and three participants were self-employed.

#### RQ1: Does a frequency-based habitization process exist?

The purpose of the Reward Pairs task was to investigate whether mere frequency of behavior would drive habitization, leading to a gradual change in behavior during test proportional to choice frequency during training. The Reward Pairs task is unique in that it manipulates option values and choice frequency independently, allowing us to disentangle the influence of value-based and frequency-based processes underlying habitization. The subsequent sections present findings relating choices, response times, and stimulus ratings in the Reward Pairs task to frequency-based learning and decision making. For these analyses, 1 participant was excluded for having more than 50% missing trials in at least one training session; 3 participants were excluded for choosing the less valuable of two stimuli during at least one training or the test session in at least 50% of trials; and 3 participants were excluded for having less than 50% accuracy in the explicit knowledge test about stimulus values at the end of the experiment. This resulted in a final sample size of n=213 participants for the analyses of RQ1. Additional analyses and information can be found in the Supplementary Information 3.1.

#### Choices

**Figure 2:**
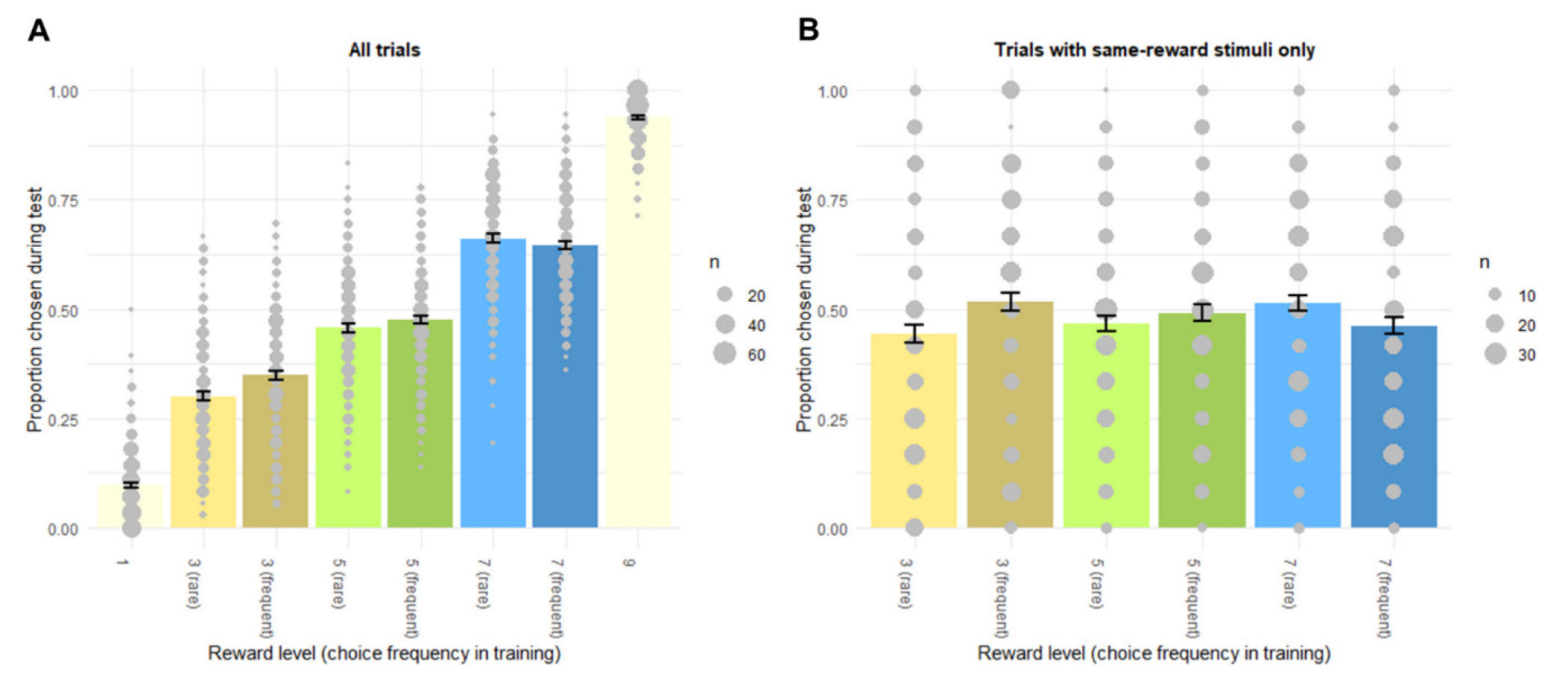
Choices during the test phase of the Reward Pairs task. (**A**) Displayed is the proportion of choice during test as a function of reward value and choice frequency during training for all trials of the test phase. (**B**) The association of choice proportions between training and test becomes more apparent when only focusing on trials in which participants chose between two stimuli with the same reward level. Error bars depict standard errors. N=213.

We tested the effect of choice frequency during training on choice frequency in the test phase (hypothesis H1a; Figure 2). Therefore, we fit a GLMM regressing choices during the test phase (left vs. right) on the difference in reward levels between the left and right option and the difference in choice frequencies between the left and right option during training sessions. Random effects of all regressors and their interaction per participant were included in the model. The difference in reward levels between the two options had a statistically significant effect on choice during the test phase (*b*=2.258, *z*=40.364, *p*<.001; Box 1) as did the difference in choice frequency during training (*b*=0.222, *z*=6.497, *p*<.001). There was no statistically significant interaction between value and choice frequency (*b*=-0.031, *z*=-1.075, *p*=.282). Thus, as one would expect, during the test phase participants chose higher-value options more often than lower-value options (which, incidentally, excludes the possibility that choice was based only on relative value, both for frequently chosen stimuli and for infrequently chosen stimuli). More importantly, participants also chose options more frequently that they had chosen more frequently during training. The results point toward both value-based and frequency-based processes underlying choices in our paradigm. Thus, hypothesis H1a was confirmed.

#### Response times

**Figure 3:**
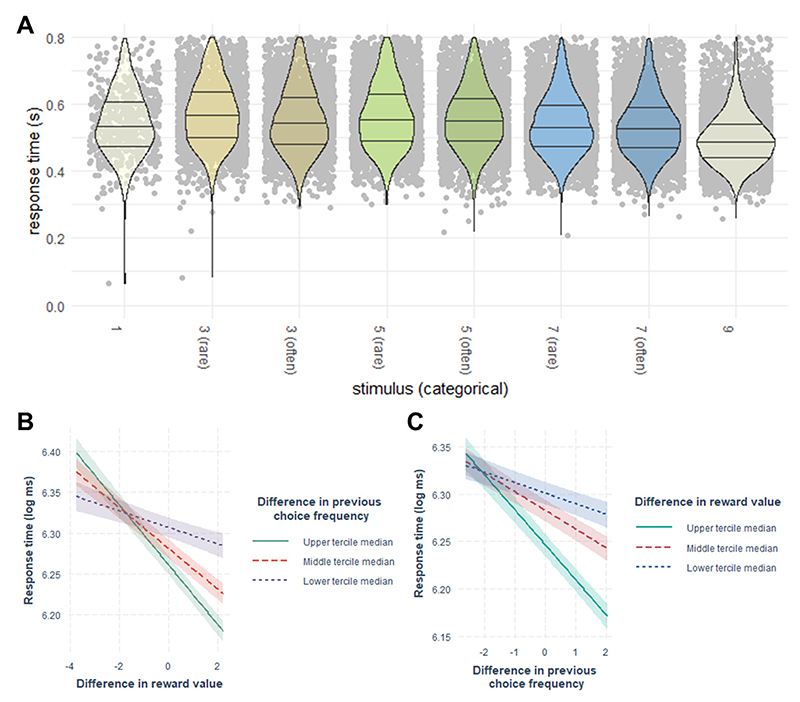
Response times during the test phase of the Reward Pairs task. **(A)** Distribution of median response times for the test phase of the Reward Pairs task dependent on reward value as well as choice frequency during training. **(B)** Reward value and choice frequency also interacted in the corresponding linear mixed effects model (Box 2). Presented are the average effects of the difference in stimulus value between chosen and unchosen stimulus for the difference in previous choice frequency between chosen and unchosen stimulus. For illustration, the difference in previous choice frequency of the chosen and unchosen option is split into terciles. We find that RTs decrease with increasing value difference and do so more steeply for stimuli chosen more frequently during training. **(C)** shows the same data as (B) but with the two variables switched. Response times were less dependent on previous choice frequency when participants chose low-valued stimuli than when they chose high-valued stimuli. N=213.

We tested whether choice frequency during the training session would affect RTs in the test phase (hypothesis H1b). Thus, we fit a LMM regressing log-transformed RTs on the difference in reward levels between the left and right option and the difference in choice frequencies between the left and right option during training sessions. Test phase RTs were statistically significantly shorter if the chosen option had a higher value than the unchosen option (*b*=-0.024, *t*(217)=-16.861, *p*<.001; Box 2) and if the chosen option was chosen more frequently during training (*b*=-0.020, *t*(196)=-13.298, *p*<.001). Moreover, there was a statistically significant interaction between the difference in reward level and the difference in choice frequencies during training (*b*=-0.012, *t*(27640)=-12.790, *p*<.001). The decrease in RTs was particularly prominent when the chosen option had a high reward level and a high previous choice frequency (Figure 3). In contrast, there was virtually no change in RTs over different reward values when previous choice frequency of the chosen stimulus was lower than that of the unchosen stimulus (negative values of the difference in choice frequency; Figure 3). Taken together, these results indicate that RTs become faster when choosing more valuable and previously more frequently chosen options. The results point toward both value-based and frequency-based processes underlying RTs in our paradigm. Thus, hypothesis H1b was confirmed.

#### Stimulus ratings

We tested whether choice frequency during the training session would affect stimulus ratings after the test phase (hypothesis H1c). Hence, we fit a LMM regressing the stimulus ratings after the test phase on reward value, choice frequency during training, and stimulus ratings before the training. Random effects of all regressors and their interaction per participant and per stimulus identity were included in the model. Stimulus ratings before the training had a statistically significant effect in stimulus ratings after the test phase (*b*=0.175, *t*(248)=5.393, *p*<.001; Box 3) as did reward value (*b*=0.593, *t*(406)=17.271, *p*<.001). In contrast, choice frequency during training did not have a statistically significant effect (*b*=-0.029, *t*(1238)=-0.667, *p*=.505). The results point toward value-based but not frequency-based processes underlying changes in stimulus valuation in our paradigm. Thus, hypothesis H1c was rejected.

#### Computational modeling

To address hypothesis H1d, we fit four choice models to participants’ behavioral data: reinforcement learning only, choice kernel only, combined reinforcement learning and choice kernel, and random choice (for details see Supplementary Information). The re-inforcement learning (RL) model learns stimulus values by repeated experience of the outcomes following each choice. In contrast, the choice kernel (CK) learns the value of choosing one stimulus simply by counting how often this stimulus is chosen. Thus, it operationalizes the idea that habit strength is proportional to choice frequency. We hypothesized the combination of RL and CK to fit participants’ behavioral data best as their choice behavior should be guided by the overt reinforcement value of stimuli (because participants’ goal in the RP task is to maximize the points earned) but also by previous choice frequency (i.e., habit strength). The random choice model served as a benchmark without learning to test the other models against.

**Figure 4:**
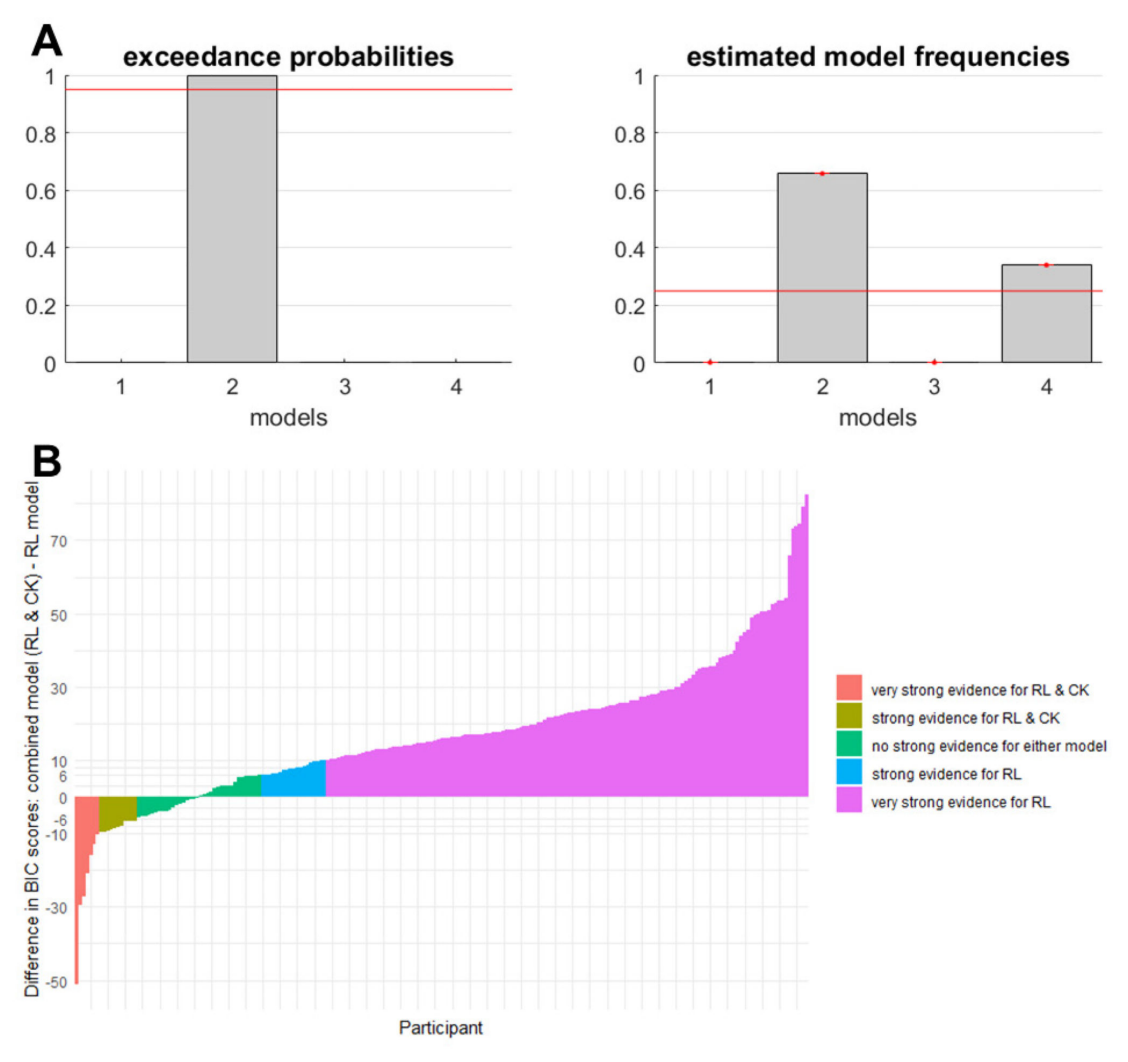
Exceedance probabilities and estimated model frequencies for choice data of the test phase of the Reward Pairs task. Over the group of participants **(A)**, the exceedence probabilities for the RL model was close to 1 signifying a very strong belief that this model was more likely generating the observed data than the other models in the comparison set. Models: 1 – random choice, 2 – reinforcement learning, 3 – choice kernel, 4 – reinforcement learning and choice kernel. On an individual level **(B)**, evidence was strongly or very strongly in favor of the combined RL and CK model for 18 participants; there was insufficient evidence for either model for 36 participants; and evidence favored the RL only model strongly or very strongly for 159 participants. Absolute BIC score differences categorized according to Raftery (1995): values between 6 and 10 signify strong evidence, values above 10 very strong evidence for one model over the other.

First, we compared model fits over the whole group of participants. Exceedance probabilities based on AIC scores yielded very strong evidence favoring the RL model without choice kernel (model 2 in Figure 4A). Thus, hypothesis H1d was rejected on the group level. Note though that according to our model recovery analyses (see Supplementary Information 2.2.1), we had a chance of around 25% to misclassify data generated by the combined RL and CK model as being generated by the RL model. This could have biased the estimated model frequencies and exceedance probability towards the RL model. Second, we explored individual model fits to get a more nuanced picture (Figure 4B): 7 participants showed very strong evidence (absolute BIC difference greater than 10) for the model combining RL and CK (red bars in Figure 4B); 11 participants showed strong evidence (absolute BIC difference between 6 and 10) for the combined model (ocher bars); for 36 participants, the absolute difference of BIC scores between the RL model and the combined model was between 0 and 6 meaning that there was insufficient evidence for either model and both were equally likely (green bars); 19 participants showed strong evidence for the RL only model (blue bars); and 140 participants had very strong evidence for the RL only model (pink bars). In summary, model comparisons on the group level did not favor the model combining RL and CK, providing little evidence for a habit process driven solely by choice frequency. However, on an individual level, the model combining RL and CK explained behavior best for a portion (about 8%) of participants, suggesting that some participants may be particularly sensitive to previous choice frequency.

##### Post-hoc analyses of computational models

**Figure 5:**
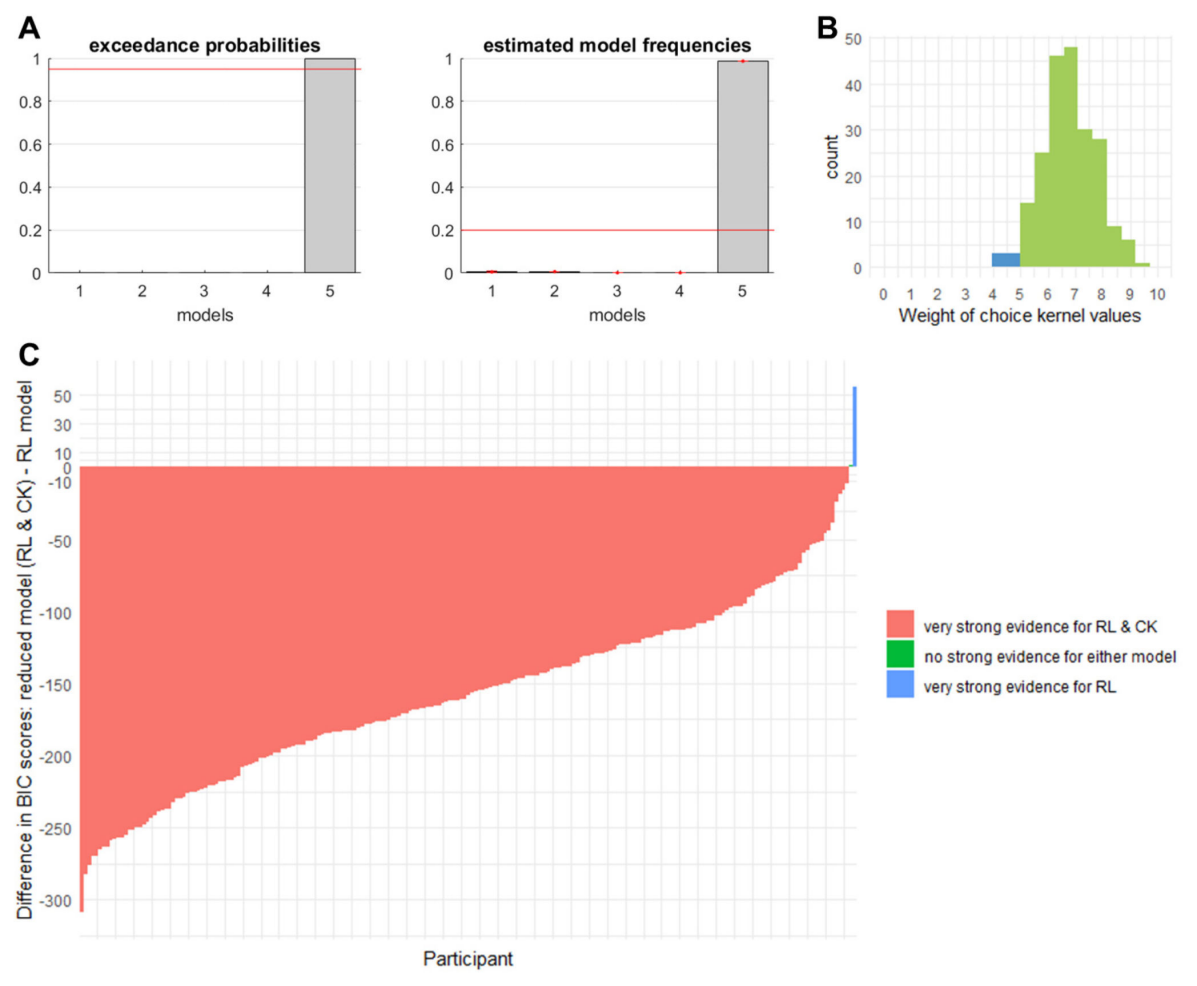
Exploratory computational modeling analyses of choice behavior during test. When including the reduced model combining RL and CK, this reduced model was strongly preferred over the group of participants with an exceedance probability close to 1 **(A)**. Models: 1 – random choice, 2 – reinforcement learning, 3 – choice kernel, 4 – reinforcement learning and choice kernel, 5 – reduced model combining RL and CK. The inverse temperature parameter of the CK, β_h_, had a distribution with a mean of 6.78 indicating an imbalance between weighing RL and CK values towards the CK **(B)**. Participants, for which the weighting parameter implied favoring RL are colored blue, those favoring CK green. On an individual level **(C)**, evidence was very strongly in favor of the reduced model combining RL and CK for 211 participants; there was insufficient evidence for either model for one participant; and evidence favored the RL only model strongly for one participant. Absolute BIC score differences categorized according to Raftery (1995): values between 6 and 10 signify strong evidence, values above 10 very strong evidence for one model over the other.

We had pre-registered the analysis of an additional model in which the inverse temperature parameter weighting the reinforcement learning value β_q_ is not free but determined by the weight for the choice kernel by β_q_=10-β_h_. Thus, a greater weight of the choice kernel automatically implied a lower weight of reinforcement learning values and vice versa. We included the parameter β_h_ in the analyses for RQs 3 and 4 to obtain an estimation of the weight participants gave to CK in their choices (see Supplementary Information 2.1.5; in the following, we call this model the fifth model or the reduced model combining RL and CK). In an additional exploratory, non-registered analysis, we repeated the model comparison including this fifth model. Including it showed strong evidence for the reduced model combining RL and CK over the whole group (Figure 5A). On an individual level, BIC score comparisons revealed evidence favoring the RL model for only one participant and indifference between the RL and the reduced model combining RL and CK for one participant. For the remaining 211 participants, BIC score differences greater than ten indicated very strong evidence in favor of the reduced model combining RL and CK. The parameter β_h_ had a mean value of 6.78 (Figure 5B) indicating a shift in balance between RL and CK towards CK (a value of 5 corresponds to equivalent weighting of RL and CK). Mean β_h_ was significantly larger than 5 (*t*(207)=26.512, *p*<.001). Therefore, these exploratory analyses revealed very strong evidence that our participants indeed took previous choice frequency into account for current choices and that RL and CK values complement each other instead of having just an additive effect.

#### Summary

Results of the analyses of choices and RTs provided support for the hypothesis that previous choice frequency on its own has an effect on later behavior in accordance with the assumption that habit strength might be proportional to behavioral frequency. In contrast, analyses of stimulus ratings did not support the hypothesis. The registered comparisons of different computational models of choice behavior during test did not support the hypothesis for registered comparisons, while an exploratory analysis including a fifth model did. As registered, we deemed support for one of the hypotheses nested in RQ1 sufficient to affirm the RQ. Thus, there was support for the assumption of a frequency-based habitization process.

### RQ2: Is external reinforcement of behavior necessary for frequency-based habitization or can the process arise also in a context without external reinforcement?

The Unrewarded Habit task manipulated instructed choice frequency in the absence of external reinforcement and thereby investigated whether choice frequency alone can drive habitization of behavior, independent of external reinforcement. The subsequent sections present findings relating choices, response times, and stimulus ratings in the Unrewarded Habit task to frequency-based learning and decision making. For these analyses, 3 participants were excluded for having more than 50% missing trials in at least one training session and 4 participants were excluded for choosing the wrong stimulus during at least one training or the test session in at least 50% of trials resulting in a final sample size of n=213 participants for analyses of RQ2. Additional analyses and information can be found in the Supplementary Information 3.2.

#### Choices

**Figure 6:**
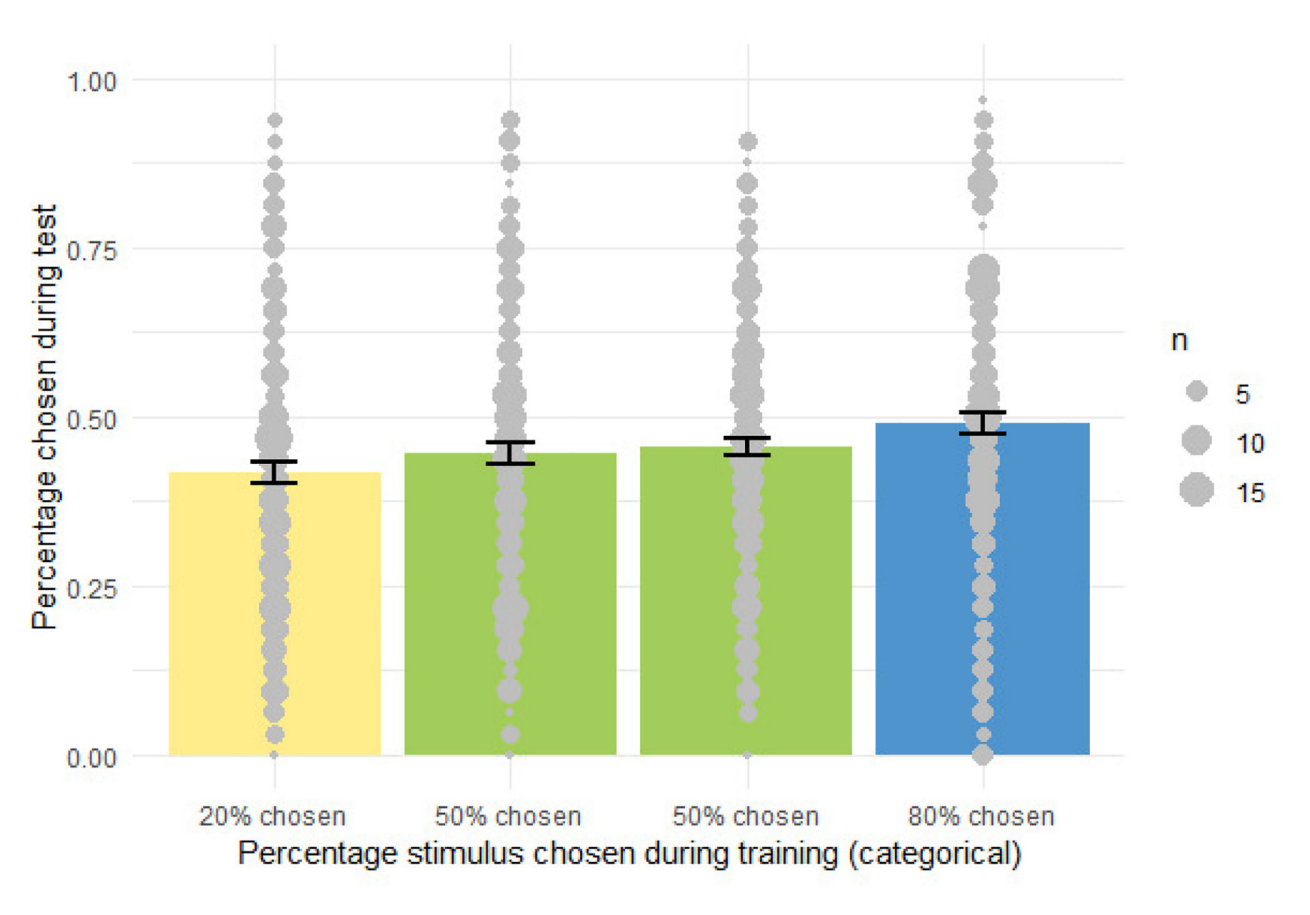
Choices during the test phase of the Unrewarded Habits task. The proportion of choosing a stimulus during test increased with instructed choice frequency during training. Note that the average percentage across all four stimuli is less than 50% because most participants missed some trials (see Supplementary Information 3.2.1). Error bars depict standard errors. N=213.

We tested the effect of choice frequency during training on choice frequency in the test phase (H2a; Figure 6). The corresponding GLMM regressed choice (left vs. right) on the difference in choice frequency during training sessions. Random effects of the regressor per participant were included in the model. Choice frequency during training had a positive and statistically significant effect on choice in the test phase (*b*=0.144, *z*=2.060, *p*=.039; Box 4). Thus, participants chose the left stimulus more frequently during test if they had chosen it more frequently during training. The findings suggest that frequency-based habit learning mechanisms are separate from value-based learning mechanisms. Thus, hypothesis H2a was confirmed. Please note that the mean score of the Social Desirability Scale (SDS-17) did not show a statistically significant correlation with the choice score of the Unrewarded Habits task (*r*=-.06 [-.20; .07], *p*=.37) and was, thus, not included as a covariate in the GLMM.

#### Response times

We tested whether choice frequency during training affected RTs in the test phase (H2b). The corresponding LMM regressed log-transformed RTs (in ms) during test on the difference in choice frequency between the chosen and the unchosen option during training, the location of stimuli in respect to training, their interaction, and the EHI score. Random effects of all regressors and their interaction per participant were included in the model. There was no significant effect of choice frequency during training (*b*=-0.004, *t*(196.909)=-1.222, *p*=.223; Box 5). Stimulus location had a statistically significant effect (*b*=0.028, *t*(204.715)=4.145, *p*<.001) indicating that stimuli presented in the same locations as during training elicit a faster response during the test phase. Previous choice frequency and stimulus location did not statistically significantly interact. Together, the response time data in the Unrewarded Habit task do not support the hypothesis of a frequency-based habit learning mechanism separate from value-based learning. Accordingly, hypothesis H2b was rejected.

#### Stimulus ratings

We tested whether choice frequencies during the training session would affect stimulus ratings after the test phase (H2c). We fit a LMM regressing the stimulus ratings after the test phase on choice frequency during training and stimulus ratings before the training. Random effects of choice frequency by participant and stimulus identity were included in the model. Stimulus ratings before the training had a statistically significant effect on stimulus ratings after the test phase (*b*=0.333, *t*(819.780)=10.295, *p*<.001; Box 6). Choice frequency during training did not have a statistically significant effect (*b*=0.041, *t*(203.760)=1.271, *p*=.205). Thus, there was no influence of frequency-based processes underlying changes in stimulus valuation in our paradigm and hypothesis H2c was rejected.

#### Summary

The results of the Unrewarded Habits task confirm the hypothesis of a frequency-based habit process in the absence of external reinforcement with regard to overt choices but not with regard to RTs or stimulus ratings. As support for one hypothesis nested in RQ2 suffices to affirm it, our results indicate that a frequency-based habit process does not necessarily rely on an externally reinforced learning process being at work simultaneously.

### RQ3: Is there a universal (i.e., paradigm independent) habitization process?

Using scores of participants’ behavior in all six habit tasks, we first examined to what extent these tasks measure the same underlying construct via correlations and confirmatory factor analysis (convergent validity). We analyzed the data separated by the modality of parameter (i.e., choice, response times, stimulus ratings, and parameters of computational models), but we report all correlations across different modalities in the Supplementary Information (Tables S6 and S7). Since all behavioral scores of the tasks were coded such that larger positive values corresponded to stronger habitual behavior, we expected positive correlations between task and questionnaire scores. As preceding analyses, we examined whether the previously used habit tasks showed the same effects as in the original studies using them. We were able to replicate previous effects for each of the four behavioral tasks (see Supplementary Information 3.3-3.6). Adding the data of three working memory capacity tasks, we then investigated the discriminant validity of the habit tasks.

### Convergent Validity

#### Choices

We found no evidence for convergent validity (i.e., all *r*’s < .50). Due to the small correlations (*r*_*max*_ = -.09 [-.22; .05], see Table 2) between the five habit paradigms providing a behavioral score based on choices, a one-dimensional measurement model did not fit the data (i.e., the models did not converge or insufficient model fits occurred according to the criteria by Schermelleh-Engel and colleagues (2003)). Further exploratory analyses (e.g., exploratory factor analysis) were not successful in identifying a common underlying habitization process.

#### Response times

The highest correlation between the three habit paradigms was *r*_max_ = .20 [.03; .37] and, thus, we found no evidence for convergent validity (i.e., all *r*’s < .50). Testing different versions (e.g., congeneric, tau-equivalent) of a one-dimensional measurement model based on a CFA led to improper solutions (e.g., negative variances) and model fits indicating a non-fitting measurement model (paired with non-significant factor loadings). Thus, we found no evidence for a common habitization process.

#### Stimulus ratings

The correlation between stimulus ratings of the Reward Pairs and Unrewarded Habits tasks was *r* = -.15 [-.31; .02], which implies no evidence for a common habitization process.

#### Computational parameters

The highest correlation between the computational modeling parameters of the Reward Pairs and Kool’s Markov sequential decision task was *r* = -.10 [-.22; .02]. Thus, the computational parameters provide no evidence for a common habitization process.

#### Summary

Independent of the behavioral measure (i.e., choices, response times, stimulus ratings, computational parameters), the data provide no evidence for convergent validity and, hence, for a common habitization process underlying these six tasks. In fact, most of the associations were near zero or even negative, providing strong evidence that the six habit tasks tap into different aspect of habits. Thus, hypothesis H3.1 was rejected.

### Discriminant Validity

According to hypothesis H3.2, we assumed that a common habitization process can be distinguished from working memory based on a two-dimensional measurement model. However, as we did not find any evidence for convergent validity of the six habit paradigms, we investigated the relation between each habit paradigm and working memory separately.

#### Preceding analyses

The correlations between the three working memory tasks were between .13 and .20 (Table 3). A one-dimensional measurement model for working memory (with two constrained factor loadings) fit the data well: *χ*^2^(1) = 0.12, *p* = .73, CFI = 1.00; RMSEA = 0.00, 90%CI = [0.00; 0.13]; SRMR = .01. In further analysis of hypothesis H3.2, the latent variable for working memory and the observed behavioral measures for each paradigm were correlated. All reported correlations were based on well-fitting models (i.e., CFI ⩾ .95, RMSEA ⩽ .08, SRMR ⩽ .10).

#### Choices

The correlations between choice scores of the habit tasks and working memory capacity provided evidence for discriminant validity of the habit tasks. All correlations were smaller than .3 (*r*_*max*_ = .23 [.01; .45]; Table 3), thus, tasks shared less than 10% variance with each other. Moreover, all absolute correlation coefficients were as large as or larger than the corresponding correlation between choice scores of the habit tasks (Table 2). However, the confidence intervals of a habit task’s correlations with all other habit tasks and with working memory capacity greatly overlapped. Accordingly, we do not interpret correlations with working memory to be substantially larger than those with other habit tasks.

#### Response times

Correlations between response time scores of habit tasks and working memory capacity provided evidence for discriminant validity of all habit paradigms (i.e., all *r*’s < .30; *r*_*max*_ = .16 [-.06; .38], see Table 3).

#### Stimulus ratings

Correlations between stimulus ratings scores of habit tasks with working memory capacity provided evidence for discriminant validity of all habit paradigms (i.e., all *r*’s < .30; *r*_*max*_ = -.02 [-.24; .20], see Table 3).

#### Computational parameters

Correlations between parameters of the computational models of habit tasks with working memory capacity provided evidence for discriminant validity of the Reward Pairs task (*r*_*max*_ = -.08 [-.30; .14], see Table 3). The parameter quantifying the balance between model-free and model-based control in the 2-Step task, ω, correlated substantially with working memory capacity (*r* = -.41 [-.63; -.19, see Table 3). As we had reversed ω so that a value closer to 1 reflected more model-free control and a value closer to 0 more model-based control, this correlation indicated more model-based control being associated with higher working memory capacity and provided only weak evidence for discriminant validity of the 2-Step task.

#### Summary

The present findings provide evidence for the discriminant validity of the frequency-based habitization tasks with regard to working memory capacity. In other words, habitization as measured with these tasks was independent of working memory, with the exception of the Markov sequential decision task 2-Step, which shared some variance with working memory.

### RQ4: How does the habitization process relate to real-life habitual behavior?

We expected a correlation of at least *r*=.3 between the common underlying habitization process and real-life habitual behavior. Due to the missing evidence of a common underlying habitization process, we investigated the relation between each habit paradigm and each habit questionnaire (i.e., COHS, SRHI, HTQ) separately. As with the analyses for convergent validity, we expected positive correlations between task and questionnaire scores of habitual behavior.

#### Choices

No choice score of any habit task showed a correlation of at least .3 with any of the habit questionnaires (*r*_*max*_ = -.24 [-.40; -.07], Table 4). Thus, the data provided no evidence for the construct validity of any of the habit tasks with this modality.

#### Response times

No response time score of any habit task showed a correlation of at least .3 with any of the habit questionnaires (*r*_*max*_ = -.15 [-.33; .03], Table 4). Thus, the data provided no evidence for the construct validity of any of the habit tasks with this modality.

#### Stimulus ratings

No stimulus rating score of any habit task showed a correlation of at least .3 with any of the habit questionnaires (*r*_*max*_ = -.12 [-.26; .02], Table 4). Thus, the data provided no evidence for the construct validity of any of the habit tasks with this modality.

#### Computational parameters

No parameter of the corresponding computational model of any habit task showed a correlation of at least .3 with any of the habit questionnaires (*r*_*max*_ = .10 [-.04; .24], Table 4). Thus, the data provided no evidence for the construct validity of any of the habit tasks with this modality.

#### Summary

The data collected in this study provided no evidence for the criterion validity of any habit task regarding real-life habits as measured with self-report questionnaires.

### RQ5: Is there a difference in validity with regard to various behavioral measures of habitual behavior?

In this exploratory research question, we examined whether one type of our behavioral measures (choices, response times, stimulus ratings, computational parameters) showed systematically stronger associations with other measures of habitual behavior. Thus, we qualitatively explored patterns in the analyses of RQs 3 and 4. However, these analyses revealed overall substantially smaller cross-correlations than expected and all confidence intervals of correlations between different behavioral measures overlapped strongly. Therefore, none of the various behavioral measures seems to be at an advantage over the others regarding associations with other measures of habitual behavior.

### Analyses regarding the association of anxiety and chronic stress with measures of habits

As a side issue of our study, we explored the associations of anxiety and chronic stress measured via the State/Trait Anxiety Inventory (STAI; Spielberger et al., 1999) and Trier Inventory for Chronic Stress (TICS; Petrowski et al., 2012; Schulz et al., 2004) with all measures of habits (see Tables S9-S11). For anxiety, we found insubstantial to small correlations (according to the classification of Gignac & Szodorai, 2016) with task measures of habits (*r*_*max*_ = -.14 [-.28; .01], Table S9) and medium to large correlations with questionnaire measures of real-life habits (*r*_*max*_ = .59 [.50; .67], Table S11). For chronic stress, we found insubstantial to medium correlations with task measures of habits (*r*_*max*_ = -.23 [-.37; -.10], Table S9) and medium to large correlations with questionnaire measures of real-life habits (*r*_*max*_ = .60 [.50; .68], Table S11). Note that we would have assumed positive correlations between the degree of anxiety/chronic stress and habitual behavior from the previous literature and some of the found associations are in opposite direction. For the relation between habit tasks and questionnaires, these findings add to previously reported mixed findings regarding the association of experimentally induced habits with trait anxiety (Gillan, 2021; Gillan et al., 2016; Glück et al., 2023; Pool et al., 2022) and stress (Friedel, 2017; Pool et al., 2022; Schwabe & Wolf, 2009; Wirz et al., 2018), though the differentiation between acute and chronic stress seems to be relevant to resolve the conflicting nature of our and previous findings.

## Discussion

Our study aimed to empirically test the notion that behavioral frequency contributes to habit strength with two new tasks and assess the associations of behavior in these tasks with paradigms used previously to measure human habits in the lab. Furthermore, we included all three currently available self-report questionnaires to measure habitual tendencies in real-life behavior. Analyses of behavior in the Reward Pairs and Unrewarded Habits tasks showed evidence for the hypothesized effect of previous choice frequency in addition to, and even independent from, external reinforcement. However, we could not confirm that the tasks we used measure the same underlying latent construct of habitual behavior. In addition, associations between behavioral tasks and self-report questionnaires were low and, thus, provided no evidence that the behavioral tasks we used measure something related to self-reported real-life habits. Thus, if taken at face value, our data suggest that habits are specific to stimuli and behaviors used rather than reflecting one common habitization process.

### Effects of frequency and reinforcement

We found an effect of previous choice frequency on future choice frequency and response times irrespective of external reinforcement value. Hence, we provide empirical evidence for the notion that current behavior is shaped in part by the mere frequency with which agents produced a specific behavior in the past (Guthrie, 1959; Miller et al., 2019). This effect was smaller than the effect of reinforcement on behavior and occurred primarily in overt choice and to a lesser degree in response times. Our findings regarding frequency-based learning processes raise the possibility that one path to the development of habits consists of repeating a behavior in a stable context, which might entrench that behavior into a routine that can be triggered automatically by context stimuli. This might be a useful lever when trying to change maladaptive behavior or develop beneficial routines (e.g., regarding physical exercise, dietary choice, or social media use). From a clinical perspective, it will be interesting to see how pure frequency-based learning relates to disorders such as addiction and compulsion. From a theoretical perspective, we would suppose this effect (or the elicitation and maintenance of behavior more generally) to be faster and stronger and to be associated with stronger extinction resistance if reinforcement is used in addition to mere repetition. This is corroborated by the comparatively smaller effect of repetition in the Unrewarded Habits compared to the Reward Pairs task.

At the same time, our findings regarding the influence of choice frequency and its interpretation of supporting increasing habitization of behavior are at odds with some reports of “instant habits” (Gollwitzer, 1999, p. 499) or the “automatic effects of instructions” (Meiran et al., 2017, p. 509) due to using implementation intentions during the instruction of a task. In these studies, the use of explicit ifthen instructions to participants increased automaticity of behavior inferred from response times or accuracy during task execution. The effect of implementation intentions cannot be explained through behavioral frequency as it is assumed to affect behavior immediately after instructions. Yet, in how far implementation intentions really induce habit-like behavior is still an open question. One study combined implementation intentions with the Symmetrical Outcome-Revaluation task (Watson et al., 2023) finding an influence of the instructions on behavior that is better explained with reduced stimulus-outcome learning instead of a change of stimulus-response learning, which is supposed to underlie habitual behavior. More research is needed to find commonalities and differences between the effects of instructions and behavioral repetition on habitual behavior.

Our findings did not suggest an interaction between behavioral frequency and reinforcement in most analyses of the Reward Pairs task. It is an interesting question for future research to test whether there are conditions in which the two effects together impact and shape habitual behavior more strongly than would be expected from adding up their individual effects. For example, it might be that repetition has a relatively larger effect in combination with substances inducing large reinforcing effects like cocaine (Ito et al., 2002; Redish et al., 2008; Robbins & Everitt, 1999) than in our experimental conditions using small amounts of snacks or money. This might then lead to faster habitization of substance use and could contribute to the development of substance use disorders. Yet, we currently lack empirical evidence for this proposition. However, the effect of behavior repetition is already used as a part of certain therapeutic approaches to treat alcohol use disorders, chronic tic disorder, and trichotillomania, that is, Cognitive Bias Modification and Habit Reversal Training (Azrin & Nunn, 1973; Bate et al., 2011; Gladwin et al., 2017; Stock, 2017). Both therapeutic approaches rely in part on the frequent repetition of instrumental behavior that is diametrical to maladaptive or burdensome routines or automatically triggered responses.

Our finding of an influence of choice frequency on value-based decision making converges with previous reports of past repetitive behavior affecting current behavior in the fields of working memory (Oberauer et al., 2015), perceptual decision making (Abrahamyan et al., 2016; Braun et al., 2018; Urai et al., 2019), and choice-induced preference changes (Brehm, 1956; Izuma & Murayama, 2013; Sharot et al., 2009). Furthermore, work on derived relations between stimuli (e.g., Liefooghe et al., 2020) showed that stimulus-stimulus associations can be learned by repetition without direct reinforcement but be inferred from overlapping stimulus material. These derived associations can even lead to automaticity as observed in a Stroop task. Thus, this learning process can work without direct reinforcement of behavior and still lead to automaticity, a hallmark feature of habits (Du et al., 2022; Seger & Spiering, 2011). Together with our findings regarding frequency-based habitization of behavior, this might create an interesting line of research examining habits on a more fundamental level, that is, on the level of the constituent features of habitual behavior. The nature of these features depends on the definition one uses in habit research. For example, Seger and Spiering (2011) suggested habits to be automatic, inflexible, learned slowly or incrementally, unconscious, and insensitive to changes in reinforcer value. Gardner (2015) names automatic impulse generation, impulses (or urges) to perform behavior, cue-dependency, and an underlying stimulus-response association as fundamental characteristics of habits. Finding approaches to examine these features will advance the field.

### Validity of habit tasks

The lack of associations across tasks seems not to have been caused by unusual behavior of our participants. Indeed, our participants showed the same behavioral effects as the participants of the original studies (see Supplementary Information 3.3-3.6). In the devaluation tasks using food outcomes by Tricomi and colleagues (Pool et al., 2022; Tricomi et al., 2009), participants did not show a change in response rates after devaluation by satiety (i.e., they seemed insensitive to changes in outcome value in their behavior, though their self-reported valuation of the devalued snack item decreased). This pattern replicates that of the original study (Tricomi et al., 2009) and the one successful replication study (Pool et al., 2022), while being at odds with previously reported failed replications (de Wit et al., 2018; Gera et al., 2023). Participants’ behavior in the outcome devaluation task by Luque and colleagues (2020) using monetary rewards revealed goal-directed changes in overt responses during devaluation but showed increased response times when participants had to switch their learned response to the now more valuable option. Thus, we replicated the original study’s (Luque et al., 2020) finding of response time switch costs associated with habitual behavior in the absence of an effect on overt choice. Participants’ behavior in the contingency degradation task was consistent with the findings for the original study’s (Vaghi et al., 2019) control group in that they goal-directedly adapted their response rates to changes in the contingency between action and outcome, that is, neither the original authors nor we found evidence for the development of habits in a non-clinical sample. In the sequential Markov decision task, participants showed behavior corresponding to a mixture of model-free and model-based behavior similar to the original study (Kool et al., 2016). Yet, there were no substantial associations between different habit tasks (see Tables 2, S6, and S7). Therefore, the lack of association seems not to be caused by our participants behaving differently than those in previous publications with these tasks and we have to conclude that the various habit tasks used in our study do not measure a single latent “habit” construct.

One possible reason for the lack of associations between habit tasks is that these tasks might not be “process pure” (Seger, 2018, p. 154), that is, all tasks might measure processes besides the development of habitual behavior. If the amount of variance related to processes different from habit is relatively large and does not overlap across tasks, the habit tasks would not share enough variance to make an association between them evident. This might also happen if habits are dependent on the specific stimuli or responses used. As habits are strongly context-dependent in general (De Houwer, 2019; Gardner, 2015), this dependency on the specifics of the tasks seems plausible. In this case, specific features of the stimulus material or characteristics of the learning environment would shape how automatic and habitual a certain behavior becomes with repetition. However, this notion appears to be incompatible with the assumption of a stable, trait-like individual propensity to develop habits, which is implicitly assumed, for example, in the literature examining differences in habitual behavior in substance use disorders (e.g., Everitt & Robbins, 2016). Assuming that a “habit trait” exists but we were not able to measure it with the tasks we used in our study, habit researchers will need to find better ways of measuring habitual behavior in the lab to uncover it.

In addition to the lack of associations between tasks, analyses examining the construct validity of habit tasks provided no evidence for an association between any experimental measure and self-reports of real-life habits. Thus, our findings point towards a lack of validity of all used tasks. This pattern of findings is similar to that of Eisenberg and colleagues (2019) who in their seminal study on self-regulation showed a lack of associations between various tasks and questionnaires supposed to measure some aspect of self-regulation. Furthermore, task measures did not show substantial associations with real-life outcomes in their study – in their perspective a result of the behavioral tasks either not successfully probing the cognitive functions that are relevant in real life or having a “contrived nature”, compromising their ecological validity. The second argument of Eisenberg and colleagues (2019) is also in line with Cattell’s (1957) proposition that task and questionnaire data in general do not show more than small correlations at best even if they are supposed to measure the same construct as their method-specific variance is vastly greater than the shared construct-related variance. Circumventing this issue by using a latent modeling approach which would have accounted for method-specific variance was not possible because the measures within the same class of method (i.e., tasks) were mostly unrelated.

More generally, our findings converge with the notion that it is not easy to elicit habits in humans performing tasks in the lab (Gera et al., 2023). Indeed, tasks specifically designed to robustly establish habits result in substantial variation in the degree of habitual behavior across participants (e.g., Pool et al., 2022, Fig. 3; van Timmeren et al., 2023, Fig. 2B; van Timmeren & de Wit, 2023, Fig. 2C). This is in line with our own findings with the previously used habit tasks (see Supplementary Information 3.3.-3.6). Accordingly, some authors even call into question whether the commonly used tasks measure habits at all (e.g., Buabang et al., 2021; De Houwer et al., 2018, 2023), while others just call into question certain aspects of habitual behavior like automaticity (Du & Haith, 2023). While our data suggest that it is in principle possible to elicit habits in some humans in the lab, they also show that, like other behaviors, habits are more situation-specific and task-dependent than often assumed.

### Limitations

Although we took great care to avoid major shortcomings of our preregistered study, there were still limitations worth keeping in mind. The sample was a convenience sample mostly comprised of university students. Thus, our sample was relatively young, likely with a decreased range of cognitive and learning abilities compared to the population. This decreased variance might in turn have led to underestimating associations across different measures and reduced generalizability of our findings. Second, testing took around 90-120 min per day over the course of five consecutive days in addition to two online sessions before the first on-site session of around 45 and 60 min, respectively. This long duration might have strained participants’ motivation and attention. Although we balanced the sequence of tasks across participants to counter order effects, reduced motivation and attention might have prevented participants from showing their maximum performance in some tasks. By consequence, this might have decreased variance of behavior across participants further, leading to additional underestimation of associations. However, this caveat is hard to avoid in this type of study and could only be counteracted with a reduced set of tasks or a larger sample with more subgroups in a planned missing design. Yet, such an approach might have created problems with the imputation procedure due to the greater proportion of missing data.

Another challenge was creating a task that works without directly reinforcing behavior, yet having control over what participants do. In the Unrewarded Habits task, we instructed participants which stimuli to choose during training and they generally complied with our instructions. However, one could argue that following instructions might have been a rewarding experience for them, thus creating an internal reinforcement signal. This effect is outside our experimental control, which is why we only speak about the effects of external reinforcement in our research questions and the interpretation of our findings.

Conflicting findings in the habit literature might be partly due to misalignments of the definition of habits used in different studies (De Houwer et al., 2023). In this study, we examined habits in terms of functional causation, that is, “as behavior that is due to certain environmental events under certain conditions” (De Houwer et al., 2023, p. 871). What we define as habitual behavior in the Reward Pairs and Unrewarded Habits tasks is supposed to be caused by previous choice frequency, while habitual behavior in the devaluation and degradation tasks (Luque et al., 2020; Tricomi et al., 2009; Vaghi et al., 2019) is defined as behavior “insensitive to changes in rewards or reward contingencies” (De Houwer et al., 2023, p. 871). These definitions of habitual behavior correspond to habits in terms of functional causation. However, with the used computational models we made an assumption about the mental causes of habitual behavior, that is, changes in subjective value representations due to reinforcement and choice frequency. Thus, we covered two of the four suggested kinds of definition of habitual behavior, but caution needs to be exercised if one intends to generalize our findings to habits that are defined descriptively or representationally.

An additional caveat is that we do not know the reliability of the used paradigms. Repeating the full procedure on the same participants was beyond the scope of this already large study. Therefore, we cannot make any statement about the reliability of the experimental measures, and it is possible that a lack thereof has led to the lack of associations between tasks and between tasks and questionnaires. However, the conclusions would remain the same. Whether the used paradigms lack reliability or validity – we as a field should invest efforts to modify tasks or generate new behavioral tasks and examine their reliability and validity before using them in further studies and claiming that the results of those studies speak to mechanisms of habitual behavior. This is a long-term endeavor based on iterations of theory building/refinement, generation of new tasks or refinement of old ones, and the empirical examination regarding their psychometric properties. This process might be facilitated by devising benchmark findings that are reproducible, generalize across methodological variations, and are theoretically informative as was done in the field of short-term and working memory research (Oberauer et al., 2018). The effect of behavioral frequency found to be at work in our study could be one of those benchmarks together with automaticity, reinforcer revaluation insensitivity, cue-dependency, and other hallmark features of habitual behavior.

## Conclusions

In conclusion, a main contribution of this study is to provide evidence that a frequency-based mechanism is at play during instrumental learning. This process works in addition to, and independent of, value-based learning processes and might be one mechanism driving habitization of behavior. As such, the effects of behavioral repetition might be considered as one of a number of characteristics defining habitual behavior in future studies and could contribute to the emergence of maladaptive behavioral routines or aid in shaping beneficial every-day habits. However, the indicators of frequency-based habitization were not associated with measures of habits from other behavioral paradigms. Moreover, those other measures were not meaningfully associated with each other, and no experimental indicator of habitual behavior was associated with self-report measures of real-life habits. This set of findings calls into question current approaches of inducing and measuring habitual behavior in the lab and calls for a rigorous reassessment of our understanding and measurement of human habitual behavior in the lab.

## Supplementary Material

Supplementary Information

## Figures and Tables

**Table 1:** Design table.

Question	Operational Hypothesis	Sampling plan (e.g., power analysis)	Analysis Plan	Interpretation given to different outcomes
RQ1: Does a frequency-based habitization process exist?	H1a: Reward Pairs task: Stimuli chosen more frequently in training are preferred in choice tests after training over stimuli less frequently chosen in training.	Testing the difference in the magnitude of correlations in H4.2 yielded the highest demands on our sample size (n=138, see below). We thus used simulations to calculate the statistical power for a small effect (equivalent to Cohen’s *d*=0.2) in the (generalized) linear mixed effects models used for H1a, H1b, H2a, and H2b representative for the remaining analyses of RQ1 and RQ2, yielding a power of >99% for the analyses with this sample size.	GLMM regressing choice (left vs. right) on value difference of the left and right stimuli, difference in previous choice frequency, and their interaction.	Main effect of difference in previous choice frequency statistically significant with higher choice probability for previously frequently chosen stimuli interpreted as evidence for frequency-based habit. Otherwise: No evidence for a frequency-based habitization process in this task based on this specific behavioral measure of habitization.
	H1b: Reward Pairs task: Stimuli chosen more frequently in training elicit faster responses in choice tests after training than stimuli less frequently chosen in training.		LMM regressing RT on value difference of chosen and unchosen options, difference in choice frequency during training, their interaction, and the EHI score (controlling for hand preference).	Main effect of difference in previous choice frequency statistically significant with faster RTs for previously frequently chosen stimuli interpreted as evidence for frequency-based habit. Otherwise: No evidence for a frequency-based habitization process in this task based on this specific behavioral measure of habitization.
	H1c: Reward Pairs task: Stimuli chosen more frequently in training elicit a stronger increase in liking ratings from the beginning to the end of the study than less frequently chosen stimuli.		LMM regressing post-study stimulus ratings on pre-study ratings, reward levels, relative choice frequency during training, and their interactions.	Main effect of previous choice frequency statistically significant with higher ratings for previously frequently chosen stimuli interpreted as evidence for frequency-based habit. Otherwise: No evidence for a frequency-based habitization process in this task based on this specific behavioral measure of habitization.
	H1d: Reward Pairs task: Comparison of computational models capturing participants’ choices favor a model with a choice kernel over models without such a kernel.		Comparison on group level based on exceedance probabilities between models of reinforcement learning, choice kernel, both, and random choice explaining participants’ choices.	Selection of model combining reinforcement learning and choice kernel as best-explaining model (i.e., greatest exceedance probability) interpreted as evidence for frequency-based habit. Otherwise: No evidence for a frequency-based habitization process in this task based on this specific behavioral measure of habitization.
RQ2: Is external reinforcement of behavior necessary for frequency-based habitization or can the process arise also in a context without external reinforcement?	H2a: Unrewarded Habit task: Stimuli chosen more frequently in training are preferred in choice tests after training over stimuli less frequently chosen in training.		GLMM regressing choice (left vs. right) on difference in previous choice frequency of the left and right stimuli.	Main effect of difference in previous choice frequency statistically significant with higher choice probability for previously frequently chosen stimuli in the same location interpreted as evidence for frequency-based habit independent from reinforcement. Otherwise: No evidence for a frequency-based habitization process independent from reinforcement in this task based on this specific behavioral measure of habitization.
	H2b: Unrewarded Habit task: Stimuli chosen more frequently in training elicit faster responses in choice tests after training than stimuli less frequently chosen in training.		LMM regressing RT on stimulus presentation location, difference in choice frequency during training of chosen and unchosen options, their interaction, and the EHI score.	Interaction effect of difference in previous choice frequency and stimulus location statistically significant with faster RTs for previously frequently chosen stimuli interpreted as evidence for frequency-based habit independent from reinforcement. Otherwise: No evidence for a frequency-based habitization process independent from reinforcement in this task based on this specific behavioral measure of habitization.
	H2c: Unrewarded Habit task: Stimuli chosen more frequently in training elicit a stronger increase in liking ratings from the beginning to the end of the study than less frequently chosen stimuli.		LMM regressing post-study stimulus ratings on pre-study ratings and relative choice frequency during training.	Main effect of previous choice frequency statistically significant with higher ratings for previously frequently chosen stimuli interpreted as evidence for frequency-based habit independent from reinforcement. Otherwise: No evidence for a frequency-based habitization process independent from reinforcement in this task based on this specific behavioral measure of habitization.
RQ3: Is there a universal (i.e., paradigm independent) habitization process?	H3.1: The data of the measures of habitual behavior from all six habit paradigms (i.e., Reward Pairs, Unrewarded Habit, outcome devaluation (Tricomi, Luque), contingency degradation, and 2-Step task) fit a one-dimensional measurement model. In addition, the correlations between all six habit paradigms are statistically significant and larger than .50 (convergent validity).	As statistically significant correlations are a prerequisite for confirmatory factor analyses, we require a sample size of 138 participants to achieve 95% power for a correlation of at least *r*=.3 (bivariate normal model, α=.05, two-tailed; G*Power 3.1.9.2; Faul et al., 2009)). In addition, we ran Monte Carlo simulations (Muthén & Muthén, 2002) to determine the required sample size of the most complex measurement model (i.e., 3 to 6 indicators per latent factor; 2 latent factors; factor loadings ≥ .50; factor correlation ≥ .35). A sample size of 190 is needed to achieve a 95% power.	Confirmatory factor analysis (CFA) and bootstrapped Pearson correlations based on behavioral scores of all habit paradigms (i.e., Reward Pairs, Unrewarded Habit, outcome devaluation, contingency degradation, and 2-Step task). Evaluation of model fit according to Schermelleh-Engel et al. (2003): X^2^ goodness-of-fit statistic, CFI ≥ .95, RMSEA ≤ .08, and SRMR ≥ .10. Comparing different models: Satorra-Bentler-scaled x2 difference test (i.e., nonsignificance indicates equal model fit).	Statistically significant and positive correlations as well as an empirically well-fitting one-dimensional measurement model are interpreted as evidence for a frequency-based habit process involved in all six tasks. Otherwise: There is no common habitization process involved in all six tasks and, thus, the six paradigms are not equally suitable for measuring the same habitization process. In this case, we will investigate in an exploratory approach which habit paradigms measure a common underlying habitization process (RQ3) and which paradigms are most strongly associated with real-life habitual behavior (RQ4).
	H3.2: The data of all six habit paradigms and the three working memory tasks will fit a two-dimensional measurement model (i.e., each a latent factor for habitization and for working memory) significantly better than a one-dimensional measurement model (discriminant validity).			A two-dimensional measurement model is interpreted as evidence that habitization is (partly) independent of working memory. Otherwise: Habit paradigms and working memory task measure the same underlying process.
RQ4: How does the habitization process measured with the experimental paradigms relate to real-life habitual behavior?	H4: The correlations between the latent habitization process and self-report questionnaire scores of real-life habitual behavior (i.e., COHS, SRHI, HTQ) are statistically significant and larger than .3.	To achieve 95% power for a latent correlation of at least *r*=.3, we require a sample size of 200 participants based on Monte Carlo simulations of the theorized structural equation model (i.e., 5-6 indicators per latent factor; 2 latent factors; factor loadings ≥ .50; factor correlation ≥ .30; Muthén & Muthén, 2002).	SEM to investigate the relation between latent factors. Evaluation of model fit according to Schermelleh-Engel et al. (2003): X^2^ goodness-of-fit statistic, CFI ≥ .95, RMSEA ≤ .08, and SRMR ≤ .10.	Statistically significant correlations with *r* ≥ .3 interpreted as evidence for criterion validity. In case of conflicting results regarding the association with the three questionnaires, the association with the SRHI will be regarded most valid. Otherwise: No evidence for criterion validity.

CFA, Confirmatory Factor Analysis. CFI, Comparative Fit Index. COHS, Creature of habit Scale. EHI, Edinburgh Handedness Inventory. (G)LMM, (generalized) linear mixed effects model. HTQ, Habitual Tendencies Questionnaire. RMSEA, Root Mean Square Error. RQ, Research Question. RT, response time. SEM, Structural Equation Modeling. SRHI, Self-Report Habit Index. SRMR, Standardized Root Mean Square Residual.

**Table 2:** Correlations between various habit tasks.

Choice	Reward Pairs	Unrewarded Habits	Luque	Tricomi
Reward Pairs	1			
Unrewarded Habits	-.09 [-.22; .05]	1		
Luque	.06 [-.12; .24]	-.09 [-.25; .06]	1	
Tricomi	.08 [-.06; .22]	.07 [-.08; .21]	.07 [-.10; .25]	1
Vaghi	-.05 [-.23; .13]	.04 [-.17; .23]	.00 [-.29; .31]	-.08 [-.29; .13]
**Response Times**	**Reward Pairs**	**Unrewarded Habits**		
Reward Pairs	1			
Unrewarded Habits	.10 [-.04; .22]	1		
Luque	.01 [-.16; .16]	.20 [.03; .37]		
**Stimulus Ratings**	**Reward Pairs**			
Reward Pairs	1			
Unrewarded Habits	-.15 [-.31; .02]			
**Computational Parameters**	**Reward Pairs, α_CK_**	**Reward Pairs, β_CK_**		
Reward Pairs, **α_CK_**	1			
Reward Pairs, **β_CK_**	-.30 [-.41; -18]	1		
Kool	-.10 [-.22; .02]	.08 [-.07; .22]		

All correlations have been bootstrapped and include 95% confidence intervals. Tasks used in previous studies are named after the first author of the respective original article: Kool – Sequential Markov Decision task; Luque – Outcome Devaluation task_LUQUE_, Tricomi – Outcome Devaluation task_TRICOMI_; Vaghi – Contingency Degradation task.

**Table 3:** Manifest correlations between various working memory capacity tasks (upper most panel) and latent correlations between a latent working memory capacity factor and the various behavioral habit scores.

Working Memory Capacity	Numerical Memory Updating	Sentence Span
Numerical Memory Updating	1	
Sentence Span	.20 [.08; .34]	1
Spatial Short-term memory	.13 [-.01; .27]	.16 [.02; .31]
**Choice**	**Working Memory Capacity**	
Reward Pairs	-.11 [-.33; .11]	
Unrewarded Habits	.23 [.01; .45]	
Luque	-.08 [-.31; .15]	
Tricomi	-.08 [-.30; .14]	
Vaghi	.18 [-.05; .42]	
**Response Times**	**Working Memory Capacity**	
Reward Pairs	-.06 [-.28; .16]	
Unrewarded Habits	.16 [-.06; .38]	
Luque	.03 [-.21; .26]	
**Stimulus Ratings**	**Working Memory Capacity**	
Reward Pairs	-.02 [-.24; .20]	
Unrewarded Habits	.00 [-.22; .22]	
**Computational Parameters**	**Working Memory Capacity**	
Reward Pairs, alpha_CK_	.02 [-.20; .24]	
Reward Pairs, beta_CK_	-.08 [-.30; .14]	
Kool	-.41 [-.63; -.19]	

All latent correlations have been bootstrapped and include 95% confidence intervals.

**Table 4:** Correlations between habit tasks and questionnaires with bootstrapped 95% confidence intervals.

Choice		Reward Pairs	Unrewarded Habits	Luque	Tricomi	Vaghi
COHS	automaticity	.15 [.02; .28]	-.01 [-.14; .12]	-.00 [-.18; .17]	.08 [-.04; .20]	-.18 [-.33; .03]
	routine	-.00 [-.15; .14]	.09 [-.04; .22]	-.12 [-.32; .08]	-.12 [-.25; .01]	-.09 [-.29; .10]
HTQ	total score	-.02 [-.16; .12]	.13 [-.00; .25]	-.10 [-.26; .08]	-.08 [-.20; .04]	-.25 [-.43; -.04]
	compulsivity	.01 [-.13; .16]	.00 [-.13; .13]	.05 [-.13; .23]	-.00 [-.14; .14]	-.26 [-.42; -.07]
	regularity	-.05 [-.20; .10]	.05 [-.09; .20]	-.01 [-.20; .17]	-.00 [-.15; .15]	-.17 [-.36; .02]
	aversion to novelty	-.00 [-.14; .13]	.18 [.05; .30]	-.20 [-.34; .04]	-.14 [-.25; -.03]	-.07 [-.24; .12]
SRHI		.01 [-.13; .14]	.02 [-.11; .15]	-.09 [-.27; .09]	-.03 [-.15; .09]	-.05 [-.23; .13]
**Response Times**		**Reward Pairs**	**Unrewarded Habits**	**Luque**		
COHS	automaticity	.11 [-.01; .23]	-.05 [-.20; .09]	-.11 [-.27; .06]		
	routine	-.02 [-.16; .13]	-.11 [-.24; .03]	-.13 [-.32; .06]		
HTQ	total score	-.00 [-.14; .14]	-.00 [-.13; .13]	-.03 [-.23; .16]		
	compulsivity	.10 [-.04; .24]	.02 [-.11; .15]	-.04 [-.23; .15]		
	regularity	-.02 [-.16; .13]	-.08 [-.21; .06]	-.09 [-.28; .09]		
	aversion to novelty	-.08 [-.21; .05]	.04 [-.09; .18]	.05 [-.13; .23]		
SRHI		-.08 [-.22; .07]	-.14 [-.27; .00]	-.06 [-.25; .13]		
**Stimulus Ratings**		**Reward Pairs**	**Unrewarded Habits**			
COHS	automaticity	.09 [-.06; .24]	-.12 [-.26; .02]			
	routine	.08 [-.06; .22]	.04 [-.09; .18]			
HTQ	total score	-.01 [-.15; .13]	.05 [-.09; .19]			
	compulsivity	.01 [-.14; .16]	-.09 [-.24; .06]			
	regularity	-.06 [-.21; .09]	.09 [-.04; .22]			
	aversion to novelty	.02 [-.12; .16]	.10 [-.04; .24]			
SRHI		.11 [-.02; .24]	.03 [-.11; .16]			
**Computational Parameters**		**Reward Pairs, alpha_CK_**	**Reward Pairs, beta_CK_**	**Kool**		
COHS	automaticity	-.05 [-.19; .09]	-.00 [-.15; .15]	.05 [-.10; .20]		
	routine	-.03 [-.16; .10]	.08 [-.06; .21]	.03 [-.13; .20]		
HTQ	total score	-.08 [-.22; .07]	.06 [-.09; .20]	.03 [-.11; .17]		
	compulsivity	-.04 [-.19; .11]	.02 [-.12; .16]	.00 [-.15; .15]		
	regularity	-.06 [-.20; .08]	.02 [-.12; .16]	.10 [-.04; .24]		
	aversion to novelty	.05 [-.19; .09]	.07 [-.07; .21]	-.02 [-.15; .11]		
SRHI		-.01 [-.18; .16]	-.05 [-.19; .08]	.07 [-.07; .22]		

Tasks used in previous studies are named after the first author of the respective original article: Kool – Sequential Markov Decision task; Luque – Outcome Devaluation task_LUQUE_, Tricomi – Outcome Devaluation task_TRICOMI_; Vaghi – Contingency Degradation task. COHS, Creature of Habit Scale; HTQ, Habitual tendencies Questionnaire; SRHI, Self-Report Habit Index.

## Data Availability

The study material, data, and analysis code are available at the first author’s OSF page: https://osf.io/qjwht/. The registration of this Registered Report can be found here: https://doi.org/10.17605/OSF.IO/EQ9JD.

## Box 1 Hierarchical logistic mixed effects regression of choices during the test phase of the Reward Pairs task

**Table:**

Generalized linear mixed model fit by maximum likelihood (Laplace Approximation) Family: binomial (logit) Formula: action ~ diff_value * diff_choice_frequency + (diff_value + diff_choice_frequency | ID) Random effects:							
	Groups	Name	Variance	Std.Dev.		Corr	
ID		Intercept	0.048	0.22			
		diff_value	0.472	0.687	0.04		
		diff_choice_frequency	0.183	0.034	-0.09	-0.44	
Number of observations: 28,320; groups: ID, 213							
Fixed effects:							
		Estimate	95% CI	OR	z value	Pr(>|z|)	
Intercept		0.023	[-0.021, 0.068]	-	1.028	0.304	
diff_value		2.258	[2.146, 2.367]	9.565	40.364	<.001	***
diff_choice_frequency		0.222	[0.155, 0.288]	1.248	6.497	<.001	***
diff_value:diff_choice_frequency		-0.031	[-0.086, 0.025]	0.97	-1.075	0.282	
Significance codes: *** *p*<0.001, ** *p*<0.01, * *p*<0.05 95% CIs (confidence intervals) based on bootstrapping with 5000 randomly drawn samples.							
Correlation of Fixed Effects:							
		Intercept	-1	-2			
(1) diff_value		0.026					
(2) diff_choice_frequency		-0.057	-0.359				
(3) diff_value:diff_choice_frequency		-0.105	-0.009	0.001			

Participants’ choices were regressed on the difference in reward values (diff_value) and previous choice frequency (diff_choice_frequency) as well as their interaction. Both variables were z-transformed to facilitate model fitting and both main effects and the interaction effect were treated as random effects for both intercepts and slopes per subject. We also included a random effect for the identity of the chosen stimulus (stim_rawID_chosen) to control for stimulus-specific effects independent of reward value and choice frequency. As anticipated (see Analysis plan), we encountered issues with non-convergence or singular analysis results and iteratively reduced the random-effects structure starting with the random effect explaining the least variance. Specifically, we eliminated the random effects of the identity of the chosen stimulus and the interaction between value and choice frequency differences. The fixed effects entail both the unstandardized regression coefficients (Estimate) and standardized odds ratios (OR).

## Box 2 Hierarchical linear mixed effects regression of response times during the test phase of the Reward Pairs task

**Table:**

Linear mixed model fit by REML. t-tests use Satterthwaite’s method Formula: log(RT)~diff_value_chosen*diff_choice_frequency_chosen+score_EHI + (diff_value_chosen+diff_choice_frequency_chosen | ID) Random effects:								
	Groups	Name	Variance	Std.Dev.		Corr		
ID		Intercept	0.007	0.084				
		diff_value_chosen	2.54E-04	0.016	-0.08			
		diff_choice_frequency_chosen	2.84E-04	0.017	-0.05	-0.3		
Residual			0.021	0.145				
Number of observations: 28,320; groups: ID, 213								
Fixed effects:								
		Estimate	95% CI	β	df	t value	Pr(<|t|)	
Intercept		6.311	[6.259, 6.361]	-	2.12E+02	245.871	<.001	***
diff_value_chosen		-0.024	[-0.027, -0.022]	-0.14	2.17E+02	-16.861	<.001	***
diff_choice_frequency_chosen		-0.02	[-0.023, -0.017]	-0.115	1.96E+02	-13.298	<.001	***
diff_value_chosen: diff_choice_frequency_chosen		-0.012	[-0.013, -0.010]	-0.038	2.76E+04	-12.79	<.001	***
score_EHI		-0.001	[-0.003, 0.001]	-0.066	2.11E+02	-1.125	0.262	
Significance codes: *** *p*<0.001, ** *p*<0.01, * *p*<0.05 95% CIs (confidence intervals) based on bootstrapping with 5000 randomly drawn samples.								
Correlation of Fixed Effects:								
		Intercept	-1	-2	-3			
(1) diff_value_chosen		-0.014						
(2) diff_choice_frequency_chosen		-0.009	-0.312					
(3) diff_value_chosen: diff_choice_frequency_chosen		-0.01	-0.115	0.053				
(4) score_EHI		-0.974	0.001	-0.001	-0.001			

Participants’ response times (RT) in ms were regressed on the differences in reward values between the chosen and unchosen options (diff_value_chosen) and the differences in previous choice frequencies (diff_choice_frequency_chosen). Both variables were z-transformed to facilitate model fitting. We also included a random effect for the identity of the chosen stimulus (stim_rawID_chosen) to control for stimulus-specific effects independent of reward value and choice frequency and a fixed-effect of no interest of the Edinburgh Handedness Inventory (EHI) to control for response time differences due to faster responding with the dominant hand. We eliminated the random effects of the identity of the chosen stimulus and the interaction between value and choice frequency differences to avoid issues of non-convergence and singular results. The fixed effects entail both the unstandardized (Estimate) and standardized (β) regression coefficients.

## Box 3 Hierarchical linear mixed effects regression of stimulus ratings in the Reward Pairs task

**Table:**

Linear mixed model fit by REML. t-tests use Satterthwaite’s method Formula: rating_post ~ rating_pre * value * frequency + (rating_pre + value | ID) + (1 | stim_rawID) Random effects:							
Groups	Name	Variance	Sth.Dev.	Corr			
ID	Intercept	0.083	0.288				
	rating_pre	0.03	0.174	-0.77			
	value	0.124	0.353	0.11	-0.67		
stim_rawID	Intercept	0.027	0.166				
Residual		0.408	0.638				
Number of obs: 1,688; groups: ID, 211; stim_rawID, 15							
Fixed effects:							
	Estimate	95% CI	β	df	t value	Pr(>|t|)	
Intercept	0.034	[-0.073, 0.141]	-	2.37E+01	0.628	0.536	
rating_pre	0.175	[0.112, 0.240]	0.171	2.48E+02	5.393	<.001	***
value	0.593	[0.526, 0.660]	0.588	4.06E+02	17.271	<.001	***
frequency	-0.029	[-0.090, 0.045]	-0.011	1.24E+03	-0.667	0.505	
rating_pre:value	0.015	[-0.045, 0.073]	0.014	8.30E+02	0.478	0.633	
rating_pre:frequency	-0.009	[-0.080, 0.061]	-0.006	1.28E+03	-0.263	0.792	
valuefrequency	-0.022	[-0.090, 0.045]	-0.015	1.24E+03	-0.631	0.528	
rating_pre:value:frequency	0.059	[-0.011, 0.130]	0.041	1.30E+03	1.652	0.099	
Significance codes: *** *p*<0.001, ** *p*<0.01, * *p*<0.05 95% CIs (confidence intervals) based on bootstrapping with 5000 randomly drawn samples.							
Correlation of Fixed Effects:							
	Intercept	-1	-2	-3	-4	-5	-6
(1) rating_pre	-0.173						
(2) value	0.161	-0.201					
(3) frequency	-0.322	0.037	-0.206				
(4) rating_pre:value	-0.06	0.23	-0.03	0.029			
(5) rating_pre:frequency	0.016	-0.528	0.019	-0.044	-0.239		
(6) valuefrequency	-0.131	0.028	-0.5	0.002	0.021	-0.014	
(7) rating_pre: value frequency	0.015	-0.222	0.018	-0.011	-0.574	-0.014	-0.034

Ratings at the beginning (rating_pre) and end of the study (rating_post) as well as stimulus values (value) and choice frequency during training (frequency) were z-transformed to facilitate model fitting. Ratings after the study were regressed on ratings before the study, value of stimuli, choice frequency during training, and their two- and three-way interactions. All effects were added as random effects on intercepts and slopes by participant as well as the raw stimulus identity (stim_rawID). We had to eliminate some random effects to avoid issues of non-convergence and singular results. Specifically, the effects with least variance explained and, thus, excluded were all interaction terms and the random effect of previous choice frequency. The fixed effects entail both the unstandardized (Estimate) and standardized (β) regression coefficients. Note that n=211 in this analysis, as two participants encountered technical errors during the stimulus ratings at the end of the study.

## Box 4 Hierarchical logistic mixed effects regression of choices during the test phase of the Unrewarded Habit task

**Table:**

Generalized linear mixed model fit by maximum likelihood (Laplace Approximation) Family: binomial (logit) Formula: action ~ diff_choice_frequency + (diff_choice_frequency | ID) Random effects:						
Groups	Name	Variance	Sth.Dev.	Corr		
ID	Intercept	0.265	0.515			
	diff_choice_frequency	0.937	0.968	0.07		
Number of observations:12,332; groups: ID, 213						
Fixed effects:						
	Estimate	95% CI	OR	z value	Pr(<|z|)	
Intercept	-0.059	[-0.140, 0.022]	-	-1.464	0.143	
diff_choice_frequency	0.144	[0.008, 0.285]	1.155	2.06	0.039	*
Significance codes: *** *p*<0.001, ** *p*<0.01, * *p*<0.05						
95% CIs (confidence intervals) based on bootstrapping with 5000 randomly drawn samples.						
Correlation of Fixed Effects:						
	Intercept					
(1) diff_choice_frequency	0.06					

The difference in previous choice frequency (diff_choice_frequency) was z-transformed before the analysis and used as random effect on both intercept and slope per subject. A random effect for the identity of the chosen stimulus (stim_rawID_chosen) had to be eliminated to avoid issues of non-convergence and singular results. The fixed effects entail both the unstandardized regression coefficients (Estimate) and standardized odds ratios (OR).

## Box 5 Hierarchical linear mixed effects regression assessing Response Times during the test phase of the Unrewarded Habit

**Table:**

Linear mixed model fit by REML. t-tests use Satterthwaite’s method Formula: log(RT) ~ stim_location*diff_choice_frequency + (stim_location*diff_choice_frequency | ID) + 1 | stim_rawID_chosen) Random effects:							
	Groups	Name	Variance	Sth.Dev.	Corr		
ID		Intercept	0.015	0.121			
		stim_location reversed	0.007	0.082	0.04		
		diff_choice_frequency_chosen	0.001	0.026	0.27	-0.37	
		stim_loc reversed: diff_choice_freq	0.001	0.033	-0.23	0.78	-0.75
Residual			0.047	0.216			
Number of observations: 12,332; groups: ID, 213								
Fixed effects:							
	Estimate	95% CI	β	df	t value	Pr(<|t|)	
Intercept	5.824	[5.750, 5.898]	-	210.325	156.362	<.001	***
stim_location reversed	0.028	[0.015, 0.042]	0.056	204.715	4.145	<.001	***
diff_choice_frequency_chosen	-0.004	[-0.011, 0.002]	-0.017	196.909	-1.222	0.223
stim_loc reversed: diff_choice_frequency_chosen	0.007	[-0.002, 0.017]	0.021	186.483	1.572	0.118
score_EHI	-0.001	[-0.003, 0.002]	-0.023	210.095	-0.664	0.507
Significance codes: *** *p*<0.001, ** *p*<0.01, * *p*<0.05 95% CIs (confidence intervals) based on bootstrapping with 5000 randomly drawn samples.							
Correlation of Fixed Effects:							
	Intercept	-1	-2	-3			
(1) stim_location reversed	-0.023						
(2) diff_choice_frequency_chosen	0.034	-0.143					
(3) stim_loc reversed: diff_choice_frequency_chosen	-0.027	0.317	-0.708				
(4) score_EHI	-0.972	-0.002	-0.006	0.004			

Participants’ response times (RT) in ms were regressed on the differences in previous choice frequencies (diff_choice_frequency_chosen), the stimulus location with respect to their location during training (stim_location_reversed), and their interaction. The difference in previous choice frequency was z-transformed to facilitate model fitting. We also included a random effect for the identity of the chosen stimulus (stim_rawID_chosen) to control for stimulus-specific effects independent of reward value and choice frequency and a fixed-effect of no interest of the Edinburgh Handedness Inventory (EHI) to control for response time differences due to faster responding with the dominant hand. As before, we eliminated the random effects of the identity of the chosen stimulus to avoid issues of non-convergence and singular results. The fixed effects entail both the unstandardized (Estimate) and standardized (β) regression coefficients.

## Box 6 Hierarchical linear mixed effects regression of ratings of the stimuli of the Unrewarded Habit task

**Table:**

Linear mixed model fit by REML. t-tests use Satterthwaite’s method Formula: rating_post ~ rating_pre + frequency + (frequency | ID) Random effects:							
Groups	Name	Variance	Sth.Dev.	Corr			
ID	Intercept	0.207	0.455				
	frequency	0.056	0.237	-0.2			
Residual		0.631	0.794				
Number of observations: 836; groups: ID, 211							
Fixed effects:							
	Estimate	95% CI	β	df	t value	Pr(<|t|)	
Intercept	0.016	[-0.063, 0.099]	-	208.494	0.393	0.695	
rating_pre	0.333	[0.269, 0.398]	0.338	819.78	10.295	<.001	***
frequency	0.041	[-0.023, 0.105]	0.041	203.76	1.271	0.205	
Significance codes: *** *p*<0.001, ** *p*<0.01, * *p*<0.05							
95% CIs (confidence intervals) based on bootstrapping with 5000 randomly drawn samples.							
Correlation of Fixed effects:
	Intercept	-1					
(1) rating_pre	0.01						
(2) frequency	-0.078	0.035					

Participants’ ratings of the stimuli after the test phase (rating_post) were regressed on ratings before the training (rating_pre) and choice frequency during training (frequency). We also model the random effects of these variables and of the stimulus identity (stim_rawID). Ratings at the beginning (rating_pre) and at the end of the study (rating_post) as well as choice frequency during training (frequency) were z-transformed before the analysis. We eliminated the random effects of the identity of the chosen stimulus to avoid issues of non-convergence and singular results. The fixed effects entail both the unstandardized (Estimate) and standardized (β) regression coefficients. Note that n=211 in this analysis, as two participants encountered technical errors during the stimulus ratings at the end of the study.
