# Supplementary material for: Characterizing Human Habits in the Lab
Source: Collabra Psychol. Author manuscript; Available in PMC 2024 Mar 8. (PMC7615722; doi:10.1525/collabra.92949)
Supplement: Supplementary Information [file EMS194390-supplement-Supplementary_Information.pdf]

# Supplementary Information
# Characterizing human habits in the lab

Stephan Nebe, André Kretzschmar, Maike C. Brandt, and Philippe N. Tobler

## 1. Supplementary information about the study procedure

The study took place on five consecutive days with an online assessment (Day 0) before the on-site assessments (Days 1-5). To have as little interference with the training sessions as possible, all tasks without extended training (outcome devaluation (Tricomi et al., 2009), Markov decision task [2-Step], and Working Memory Capacity tasks) and the questionnaires were completed before onset of the training of the other tasks. Specifically, the Working Memory Capacity and first outcome devaluation (Tricomi et al., 2009) tasks were completed on Day 1 before all other tasks, whereas the 2-Step task and questionnaires (Table S1) were completed online on Day 0. The Unrewarded Habit and Reward Pairs tasks start on Day 1 (five training sessions in total), while the second outcome devaluation task (Luque et al., 2020) and the contingency degradation started on Day 2 (four training sessions in total; Table S1). From then onwards, these four tasks were performed each day including Day 5, on which a test phase followed the last training.

Our six experimental paradigms used different kinds of behaviors as measures of interest. These included choices, response times (RTs), stimulus ratings, and free parameters from computational models (Table S2 gives an overview of which behavioral measures are used in which task). Where applicable, we tested the hypotheses of our Research Questions separately for the different behavioral measures. This approach allowed us to test whether one behavioral measure outperformed the others in terms of its validity (see Research Question 5).

*Table S1. Procedure of training and test phases of the task paradigms and the questionnaires.*

| | Day 0 | Day1 | | Day 2 | | Day3 | Day 4 | Day 5 | |
|---|---|---|---|---|---|---|---|---|---|
| | Test | Training | Test | Training | Test | Training | Training | Training | Test |
| Reward Pairs task | - | + | - | + | - | + | + | + | + |
| Unrewarded Habit task | - | + | - | + | - | + | + | + | + |
| Outcome devaluation task$_{Tricomi}$ | - | + | + | - | - | - | - | - | - |
| Outcome devaluation task$_{Luque}$ | - | - | - | + | + | + | + | + | + |
| Contingency degradation task | - | - | - | + | + | + | + | + | + |
| Working Memory Capacity tasks | - | - | + | - | - | - | - | - | - |
| Markov decision task (2-step; online) | + | - | - | - | - | - | - | - | - |
| Self-report habit index (SRHI; online) | + | - | - | - | - | - | - | - | - |
| Creature of habit scale (COHS; online) | + | - | - | - | - | - | - | - | - |
| Habitual Tendencies Questionnaires (HTQ; online) | + | - | - | - | - | - | - | - | - |
| Edinburgh Handedness Inventory (EHI; online) | + | - | - | - | - | - | - | - | - |
| Social Desirability Scale (SDS-17; online) | + | - | - | - | - | - | - | - | - |

*Table S2. Behavioral measures used in each of the six experimental paradigms. This table shows which measures (choices, response times, stimulus ratings, computational parameters) were measured in which paradigm. Analyses regarding Research Question 1-4 were performed for each available measure.*

| | choice | response times | stimulus ratings | computational parameters |
|---|---|---|---|---|
| Reward Pairs task | + | + | + | + |
| Unrewarded Habit task | + | + | + | - |
| Outcome devaluation task$_{Tricomi}$ | + | - | - | - |
| Outcome devaluation task$_{Luque}$ | + | + | - | - |
| Contingency degradation task | + | - | - | - |
| Markov decision task (2-step) | - | - | - | + |

## 1.1 Supplementary information on contingency degradation paradigm

The contingency degradation task was a modified version of a previously used paradigm (Vaghi et al., 2019). The used probabilities of an outcome contingent and non-contingent on participants' behavior can be found in Table S3.

*Table S3. Programmed contingencies of outcomes in the Contingency Degradation task. The table also indicates how often blocks of these contingencies were repeated on the five days of the study, and provides a high-level description of blocks with regard to contingency and degradation.*

| | Programmed contingency | | | Repetition on Day | | | Contingency (degradation) |
|---|---|---|---|---|---|---|---|
| | P(O\|A) | P(O\|~A) | ΔP | 2 | 3-4 | 5 | |
| 1 | 0.0 | 0.6 | -0.6 | 2 | 0 | 2 | negative contingency (fully degraded) |
| 2 | 0.0 | 0.3 | -0.3 | 2 | 0 | 2 | negative contingency (fully degraded) |
| 3 | 0.3 | 0.6 | -0.3 | 1 | 1 | 1 | negative contingency (partially degraded) |
| 4 | 0.3 | 0.3 | 0.0 | 1 | 1 | 1 | zero contingency (partially degraded) |
| 5 | 0.3 | 0.0 | 0.3 | 2 | 4 | 2 | positive contingency (not degraded) |
| 6 | 0.6 | 0.0 | 0.6 | 2 | 4 | 2 | positive contingency (not degraded) |
| 7 | 0.6 | 0.3 | 0.3 | 1 | 1 | 1 | positive contingency (partially degraded) |
| 8 | 0.6 | 0.6 | 0.0 | 1 | 1 | 1 | zero contingency (partially degraded) |

## 1.2 Supplementary information on Reward Pairs task

The Reward Pairs task comprised eight different stimuli and a specific scheme of pairing two stimuli per trial in the daily training sessions. Table S4 lists each stimulus pair presented during the training session.

*Table S4. Identities, reward values, and frequency of all stimulus pairs presented (in random order) during each training session of the Reward Pairs task. Please note that the presentation location (left vs. right) of stimuli 1 and 2 were balanced for each pair.*

| Stimulus 1 | Stimulus 2 | Value stimulus 1 | Value stimulus 2 | Frequency |
|---|---|---|---|---|
| 1 | 2 | 1 | 3 | 10 |
| 1 | 3 | 1 | 3 | 30 |
| 2 | 4 | 3 | 5 | 30 |
| 3 | 5 | 3 | 5 | 10 |
| 4 | 6 | 5 | 7 | 10 |
| 5 | 7 | 5 | 7 | 30 |
| 6 | 8 | 7 | 9 | 30 |
| 7 | 8 | 7 | 9 | 10 |

# 2. Computational models

## 2.1 Computational models of choices in training and test phases of the Reward Pairs task

We used computational models of overt choices to examine possible mechanisms driving decision making. Therefore, we adapted and combined previously published choice models for the Reward Pairs task. The full computational model consisted of temporal difference reinforcement learning and a kernel capturing choice frequency. Specifically, we considered four different models: (1) random choice behavior with a possible side bias, (2) reinforcement learning, (3) choice kernel, and (4) combined reinforcement learning and choice kernel. During the preparation of this Registered Report, we had tested various additional models to capture additional aspects of choices (including response stickiness, decreasing learning rates over the course of training, forgetting of values between training sessions, separate learning rates for chosen and unchosen options, learning of relative in addition to absolute reinforcement values of stimuli, and combinations of these processes). Most of these models had insufficient model or parameter recovery, were unable to recover the staircase pattern of choice behavior in the simulated data of the model combining reinforcement learning and a choice kernel, or added little information regarding our specific Research Questions. Therefore, we decided to limit ourselves to the four models that were necessary and sufficient to examine our hypotheses. However, we provided the code to simulate data with and fit all of these models in the OSF repository associated with this Registered Report.

### *2.1.1 Reinforcement learning*

We used a standard model-free reinforcement learning model (Rescorla & Wagner, 1972; Sutton & Barto, 1998). Individual value expectations for each stimulus were initialized to the participant-specific pre-training ratings. For this analysis, we rescaled the stimulus ratings, because they could range from zero to 100, while the reinforcement learning values could range from zero to nine. We therefore subtracted the individually smallest liking rating of the

stimulus set chosen to be used in the experiment from each rating and divided the result by 11.15. Thus, the largest theoretically possible (but very unlikely in this set of rather neutral stimuli) difference of 100 between the least and best liked stimulus in the chosen set would be rescaled to 8.97, which is close to the largest possible reinforcement learning value (i.e. 9). With the feedback in each trial (given for both chosen and unchosen options), participants updated their value expectations according to

$$Q_{t+1}^{k} = Q_{t}^{k} + \alpha_q (r_t - Q_{t}^{k})$$

$Q_t^k$ corresponded to the reinforcement learning value associated with choosing a particular stimulus *k* in trial *t*, $(r_t - Q_t^k)$ the reward prediction error comparing the actually received $(r_t)$ to the expected outcome $(Q_t^k)$ and $\alpha_q$ the learning rate determining the step size of the value update, bounded by 0 and 1. The learning rate was a free parameter, which was estimated for each participant to provide the best fit to the choice data using maximum likelihood optimization. We used the same learning rate for chosen and unchosen options as previous research found no indication of a difference (Findling et al., 2019).

#### ***2.1.2 Choice kernel***

The choice kernel of a choice option increases whenever participants select the option (irrespective of the outcome of choice; Miller et al., 2019; Wilson & Collins, 2019). This choice frequency-based process operationalized habit strength in our computational framework. Participants started with a habit strength of zero for each stimulus. After each decision, these strengths were updated according to

$$H_{t+1}^{k} = H_{t}^{k} + \alpha_h (a_t - H_{t}^{k})$$

$H_t^k$ denotes the habit strength associated with stimulus *k* in trial *t*. The learning rate $\alpha_h$ determined the step size of the update, bounded between 0 and 1. The action $a_t$ was set to 1 for the chosen and 0 for the unchosen stimulus. Again, the learning rate was a free parameter estimated for each individual participant to best fit their choice data.

### *2.1.3 Random choice with possible side bias*

When one conceives of the model space as a spectrum with the full model including all of the mentioned parameters on one end, then the other end of this spectrum is a model with random choice behavior combined with a possible side bias (in a task with lateralized stimulus presentation). The side bias captures the tendency to choose the left or right stimulus irrespective of stimulus identity. We implemented this model of random choice behavior in the Reward Pairs task by setting all parameters in the softmax function (see below) to zero except for the side bias parameter *b* (see Wilson & Collins, 2019, p.7, Box 2, Model 1, for a similar approach to modelling random choice). The side bias parameter *b* could have values in the range of 0 to 5 with values close to 0 denoting a strong tendency towards the right, close to 5 favoring the left, and a value of 2.5 meaning no bias at all. According to our simulations, this range of the side bias parameter was large enough to allow for fine-grained differences in choice tendency while not being too large to lose the capacity to differentiate at the extremes of the range. The side bias was the free parameter of the random-choice model.

### *2.1.4 Calculating the probability of choice*

All model parameters were combined to calculate the probability of choice using a softmax function. This function computed the probability of choosing the left (option 1) over the right option (option 2) in trial *t*:

$$p_t^1 = \frac{exp(Q_t^1 * \beta_q + H_t^1 * \beta_h + b)}{exp(Q_t^1 * \beta_q + H_t^1 * \beta_h + b) + exp(Q_t^2 * \beta_q + H_t^2 * \beta_h + (5 - b))}$$

The current estimates of value ($Q_t^k$) and frequency-based habit strength ($H_t^k$) were weighted by $\beta_q$ and $\beta_h$, respectively. The inverse temperature parameters $\beta_q$ and $\beta_h$ were bounded by 0 and 10 and estimated for each participant.

We fitted all four models to individual data of all five training sessions. In each participant, we used maximum likelihood optimization to identify the best fitting values of all free parameters. These parameters served to model the participant's data in the test session,

calculating the likelihood of each model given the data and model parameters. Then, we computed a BIC (Schwarz, 1978) for each model and participant for the training and test phase separately and selected the best-fitting model on the participant level by the minimum BIC. We used the criteria described by Raftery (1995) interpreting BIC differences of 6-10 as strong, and >10 as very strong evidence for the model with the smaller BIC. For model comparison on the group level, we calculated the exceedance probability (Rigoux et al., 2014; Stephan et al., 2009) of each model in the set using the VBA toolbox (Daunizeau et al., 2014).

#### *2.1.5 Analyses including the computational models*

We used the computational modelling approach to test the hypothesis that a frequency-based learning process influenced choices during training and test phases of the Reward Pairs task (H1d) and to examine the relations between the Reward Pairs task and other experimental tasks and questionnaires measuring habitual behavior (RQs 3 and 4). To test the hypotheses regarding associations with other tasks and questionnaires, we fitted an additional model, in which the weighting of values from reinforcement learning and the choice kernel complemented each other. That is, if the influence of reinforcement learning on choices increased, the influence of the choice kernel decreased and vice versa. The parameter weighting reinforcement learning and choice kernel against each other ($\beta_q$=10-$\beta_h$) provided an individual estimate of the relative influence of the two processes on choice (equivalent to ω of the 2-Step task; Otto et al., 2013) and entered analyses of the association with other measures of habits.

### 2.2 Model diagnostics

We tested parameter recovery and model recovery of the computational models as well as whether they could descriptively recover the behavioral phenomenon of interest. For this purpose, we simulated 1,000 participant data sets of the training and test phases for each of the four models in the model space with uncorrelated values of the free parameters of each model sampled from a uniform distribution over the possible range of parameter values (i.e.

values between 0 and 1 for the learning rate $\alpha$, between 0 and 10 for $\beta$, and between 0 and 5 for $b$).

#### ***2.2.1 Model recovery***

We took subsets of 200 simulated participants (due to the high time requirement of this process) for each model and fit all models to these data sets. Then we calculated BIC scores for each simulated participant for each model checking how often the data generating model was favored by the model selection. This procedure allowed us to assess the amount of uncertainty about the favored model for real participant data. The confusion matrix (Figure S1) shows how often the data-generating model (rows in Figure S1) was identified as the best-fitting model (columns in Figure S1) by model comparisons based on the BIC. Ideally, the diagonal would have values close to one, meaning close to 100% of data sets generated with a specific model are identified correctly, and values close to zero for all off-diagonal cells. This was the case for the random choice, reinforcement learning, and choice kernel models for both training and test phases. The model combining reinforcement learning and choice kernel was less frequently identified correctly (68% of cases in the simulated data sets). Thus, for a participant for whom the combined model was identified to fit the data best, it was very likely the actual data generating model (out of the tested set of models). In contrast, if the best fitting model was the pure reinforcement learning model, there was a chance of roughly 25% that the actual data generating model was the model combining reinforcement learning and choice kernel. Moreover, if the best fitting model was the combined model, there was little chance that the actual model was the pure reinforcement learning model. As our Research Question aimed at identifying the combined model, the pattern of potential confusions made our analyses more conservative.

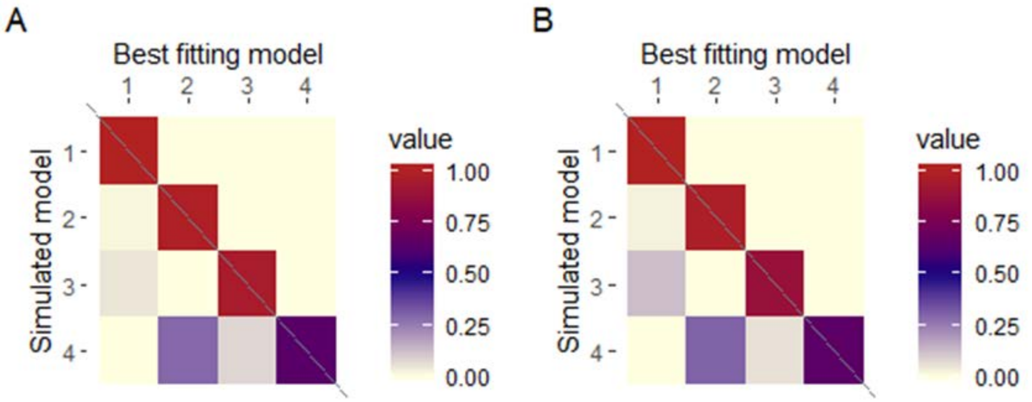


*Figure S1. Confusion matrix of simulated data for the four models (**A**, training sessions; **B**, test phase). The models are (1) random choice, (2) reinforcement learning, (3) choice kernel, and (4) reinforcement learning and choice kernel.*

### *2.2.2 Parameter recovery*

We applied the same procedure to estimate the best-fitting parameter values for each simulated participant for each model that we used to analyze real participant data. This procedure allowed us to test how well we could recover the data-generating parameter values and, thus, how much uncertainty about the parameter values from real participant data we had to take into account. The resulting measure captured reliability of the estimated parameters and should be at best close to one, but stochasticity in action selection in the data simulations introduces noise, which in turn decreases the reliability of parameter recovery. We followed previously published guidelines to evaluate these recoverability estimates. As described in Cichetti (1994), measures of internal consistency were deemed unacceptable below .7, fair between .7 and .79, good between .8 and .89, and excellent above .89, while measures of intraclass correlation were deemed poor below .4, fair between .4 and .59, good between .6 and .74, and excellent above .74. Lance, Butts, & Michels (2006) recommend a reliability of .8 as cut-off for acceptable reliability in basic research. Parameter recovery was good overall (Table S5) with a median correlation between the true, data-generating and the estimated parameters over all five models and parameters of *r*=.814. However, the correlation between true and estimated parameters varied over the models (see last column of Table S5) and the

individual parameters (see last row in Table S5). Of most interest was the recoverability of the parameters $\alpha_h$ and $\beta_h$ of the model combining reinforcement learning and choice kernel with complementing inverse temperatures (i.e. $\beta_q = 10 - \beta_h$), because these two parameters were extracted and used in analyses regarding the association with other experimental paradigms and questionnaires measuring habits (H3.1, H3.2, H4.1, & H4.2). Recoverability of $\beta_h$ was excellent (r=.956), while the recoverability of $\alpha_h$ was fair (r=.576). Therefore, $\alpha_h$ was not used in the analyses of associations with other tasks and questionnaires and we focused on $\beta_h$ as a measure of the trade-off between reinforcement learning and choice kernel values in determining individual decision making.

*Table S5. Parameter recovery for all models. Each cell contains the Pearson correlation between the true, data generating parameter values with the estimated/recovered parameter values based on 1,000 simulated participants per model. The last column displays the median correlation of the respective model over all parameters, the last row the median of correlations of the respective parameter over all models, and the entry in the last column and last row the median over all models and parameters.*

| Model | $\alpha_h$ | $\beta_h$ | $\alpha_q$ | $\beta_q$ | bias | Median (model) |
|---|---|---|---|---|---|---|
| Random choice | - | - | - | - | .998 | .998 |
| Choice kernel | - | - | .732 | .941 | - | .837 |
| Reinforcement learning | .881 | .929 | - | - | - | .905 |
| Reinforcement learning & choice kernel | .668 | .746 | .674 | .904 | - | .710 |
| Reinforcement learning & choice kernel ($\beta_h$=1-$\beta_q$) | .576 | .956 | .667 | - | - | .667 |
| Median (parameter) | .668 | .929 | .674 | .922 | .998 | .814 |

### *2.2.3 Recovery of value- and frequency-based choice*

Finally, we checked whether the data simulated with different computational models could capture dependence of choices in the simulated test session on reward level and choice frequency during training (Figure S2). Only the model combining reinforcement learning and the choice kernel displayed the assumed choice pattern, which was also evident in the pilot data: stimuli were more likely to be chosen during test if they were associated with higher reward levels and, within one reward level, higher choice frequency during training (i.e. a staircase pattern over all 8 stimuli; Figure S2D). The model including only reinforcement learning (Figure S2B) yielded choices only dependent on the reward level but without

differentiation between frequently and rarely chosen stimuli within one reward level. The models of random choice and the choice kernel showed on average no effects of previous choice frequency and reward value, that is, each stimulus had a chance of being chosen of 50% over the whole group. The average choice frequency of 50% for each stimulus and the wide spread in the choice kernel model across individuals were a result of purely random choices at the beginning of training, in which each stimulus had a value of zero. As the choice kernel only added up previous choice frequency, this initial random selection of stimuli led to an exacerbation of preference for these stimuli independent of any other stimulus feature. The higher the learning rate and/or inverse temperature of the choice kernel model, the more extreme the preference for the stimuli that were chosen randomly at the beginning. Thus, each simulated agent could have extreme preferences for individual stimuli in the test phase, but as these preferences were based on random choices at the beginning of training, the average preference for any stimulus was still near 50% over the group of simulated participants.

To illustrate that the choice kernel model indeed learned preferences for specific stimuli, we additionally assigned each of the eight stimuli to a bin according to the preference of each simulated agent at the end of the training sessions. The bins represented stimuli with a habit strength of 0 to 0.2, 0.2 to 0.4, 0.4 to 0.6, 0.6 to 0.8 and 0.8 to 1 at the end of the training sessions. We then displayed the average choice frequency during the test phase for the binned stimuli (Figure S3). As expected, the choice kernel model acquired (choice history-dependent) stimulus preferences over training (independent of any objective stimulus feature) and applied these preferences in the test phase.

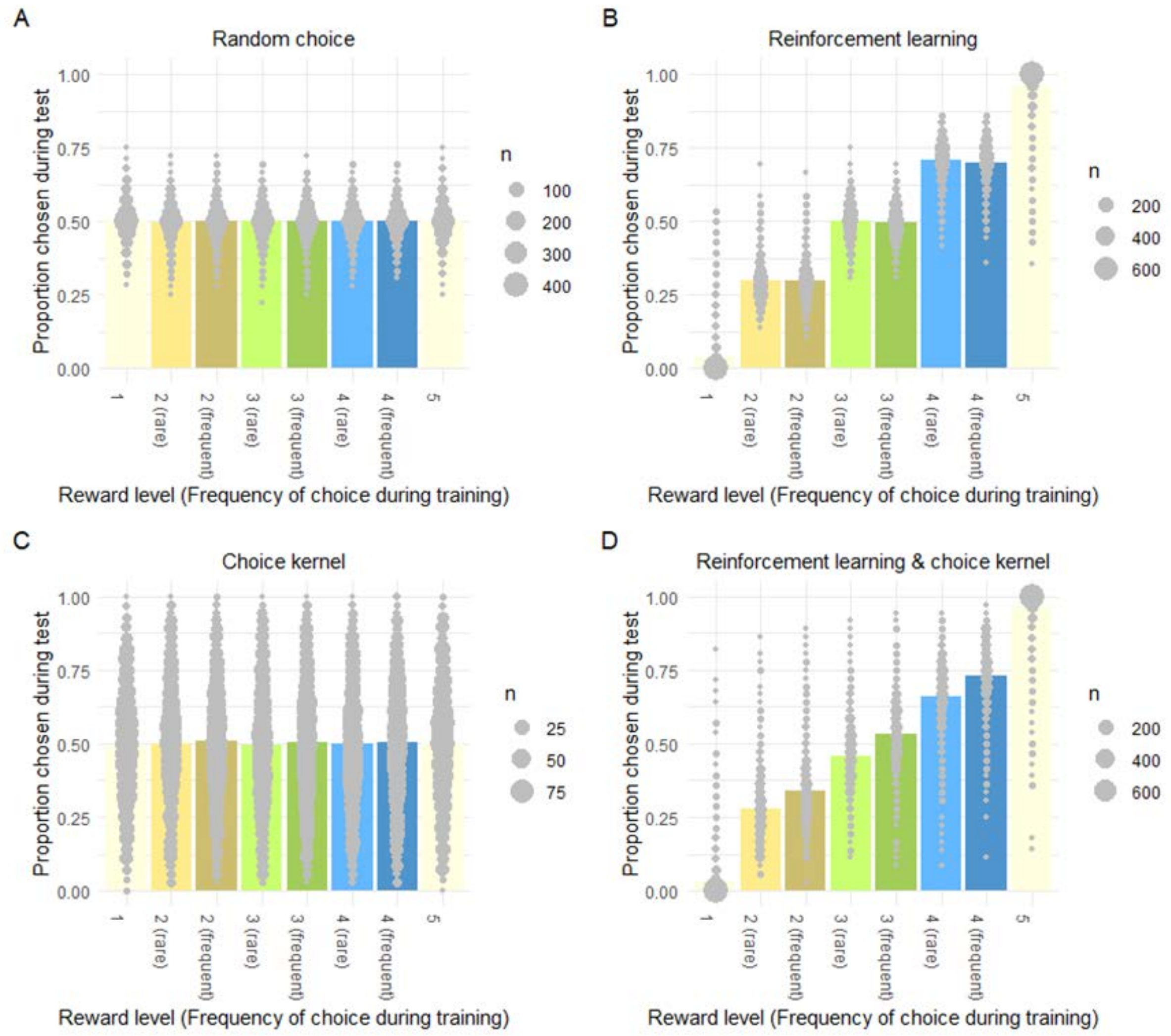


*Figure S2. Choice patterns during the test phase of the Reward Pairs task in simulated data. Displayed is the proportion of choosing stimuli as a function of their reward value (1 to 5 points) and the frequency they were chosen during the training sessions (frequent, rare). Comparing the four models (**A**: random choice, **B**: Reinforcement learning, **C**: Choice kernel, **D**: Reinforcement learning & choice kernel), only the fourth model, which combined reinforcement learning and choice kernel, demonstrated the full staircase pattern over reward values and previous choice frequency hypothesized from theory and observed in the pilot data.*

The second feature of individual choices we looked at for model validation was the psychometric curve (Wichmann & Hill, 2001) as originally derived from psychophysics experiments. In our case, the curves reflected the probability of choosing a stimulus in each trial as a function of the value difference between the two options presented in the three data sets simulated with each of the computational models. As expected for probability calculated via a softmax function, they followed an S-shape with asymptotes approaching a choice probability of zero for negative value differences and one with positive value differences (Figure S4). In other words, the probability of choosing a stimulus increased with increasing

value difference in favor of that stimulus. As the stimulus values in the random choice model always remained at zero, we could not draw a psychometric curve for this model.

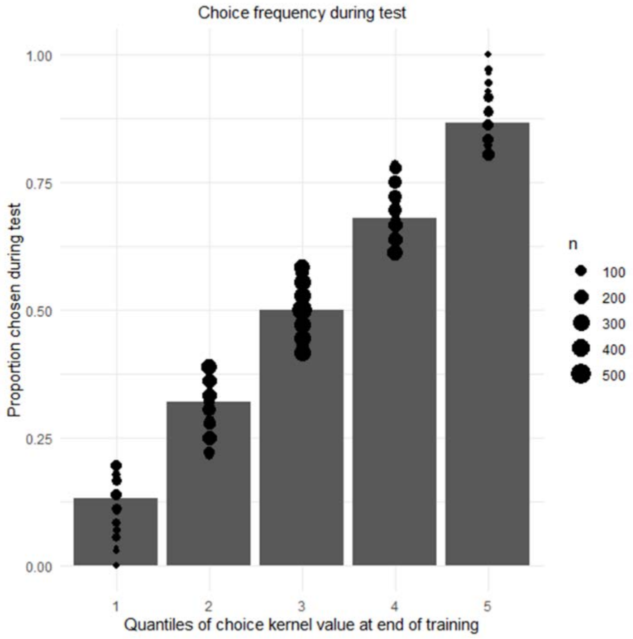


*Figure S3. Choice pattern of the choice kernel model in the test phase for the stimuli binned according to their choice kernel value at the end of the training sessions. The choice kernel model learned (choice-history dependent) preferences for specific stimuli in training and applied them to the test phase, even though they were independent of any objective stimulus feature.*

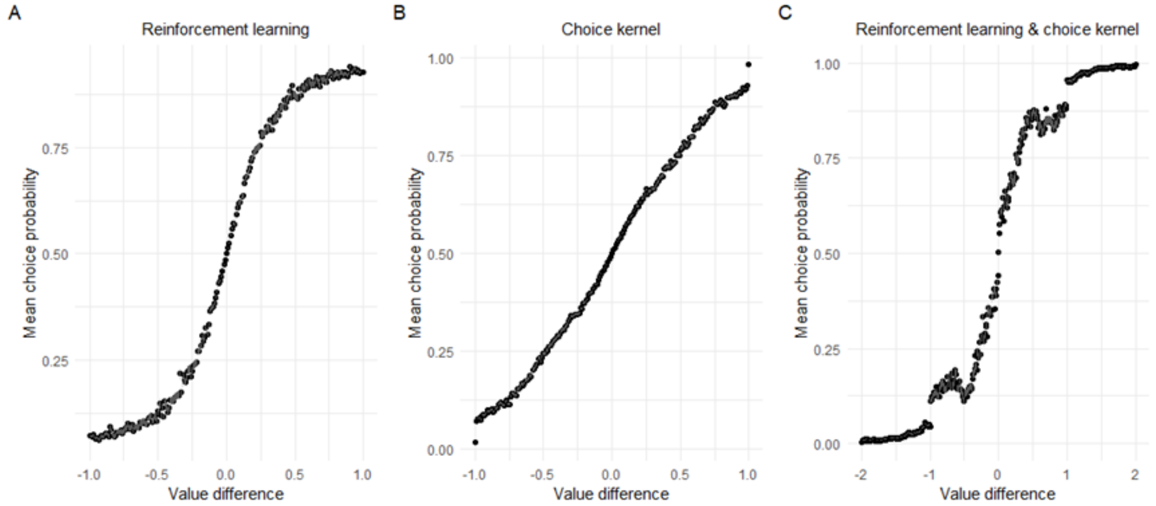


*Figure S4. Psychometric curves showing the value difference between left and right option on the x-axis and the choice probability on the y-axis. The mean choice probability for value differences was rounded to two decimals and the choice probabilities were averaged over all trials in the same value-difference bin. (**A**) reinforcement learning, (**B**) choice kernel, and (**C**) combined model. The (mostly extremely small) standard error of the mean is represented by the grey area around each data point.*

**2.3 Sequential Markov Decision Task (2-Step)**

For the 2-Step task (Figure S5) we fit behavior to a hybrid of model-free and model-based reinforcement learning (Daw et al., 2011; Kool et al., 2016). The model-free part consisted of a state-action-reward-state-action SARSA (λ) reinforcement learning algorithm (Rummery & Niranjan, 1994). where a model-free value for a state-action pair $Q_{MF}(s,a)$ was updated in each stage *i* and trial *t* according to:

$$Q_{MF}(s_{i,t},a_{i,t}) = Q_{MF}(s_{i,t},a_{i,t}) + \alpha\delta_{i,t}e_{i,t}(s,a)$$

with $\alpha$ corresponding to the learning rate or step size parameter, $e$ to the eligibility trace, and $\delta$ to the prediction error. The prediction error at the first stage was the difference between the value of the second stage reached after choice in the first stage and the value of this first stage state-action pair: $\delta_{1,t} = Q_{MF}(s_{2,t},a_{2,t}) - Q_{MF}(s_{1,t},a_{1,t})$. The prediction error at the second stage was the difference between the actually received outcome and the expected outcome represented by the second-stage value: $\delta_{2,t} = r_{2,t} - Q_{MF}(s_{2,t},a_{2,t})$. The eligibility trace was set to 0 at the beginning of each trial and then updated according to $e_{i,t}(s_{i,t},a_{i,t}) = e_{i-1,t}(s_{i,t},a_{i,t}) + 1$ before the Q value was updated (Kool et al., 2017). The model-based part used Bellman's equation to compute the value of a state-action pair by combining the maximum possible value of the next states with the probability to reach these states:

$$Q_{MB}(s_A,a_j) = P(s_B|s_A,a_j) \max_{a \in \{a_A,a_B\}} Q_{MF}(s_B,a) +$$

$$P(s_C|s_A,a_j) \max_{a \in \{a_A,a_B\}} Q_{MF}(s_C,a).$$

The model-based algorithm differentiated between the two 2nd-stage states, $s_B$ and $s_C$. Thus, it took the conditional probability of reaching either of the two 2nd-stage states through an action $a_j$ in one of the 1st-stage states $s_A$ (which did not need to be differentiated, because they were equivalent regarding the transition structure to the 2nd stage) and multiplied this probability with the highest valued state-action pair on the 2nd stage. Model-free and model-based values were then combined and weighted by an individual free parameter $\omega$:

$$Q_{net}(s_A, a_j) = \omega Q_{MB}(s_A, a_j) + (1 - \omega) Q_{MF}(s_A, a_j)$$

The net value of each option served to calculate choice probabilities with a softmax function:

$$P(a_{i,t} = a | s_{i,t}) = \frac{\exp\left(\beta\left[Q_{net}(s_{i,t}, a) + \pi * rep(a) + \rho * resp(a)\right]\right)}{\sum_{a'} \exp\left(\beta\left[Q_{net}(s_{i,t}, a') + \pi * rep(a') + \rho * resp(a')\right]\right)}$$

The free parameters $\pi$ and $\rho$ captured two types of stickiness. If the first-stage choice was the same as in the preceding trial, rep(a) was set to 1 (and 0 otherwise). Thus, $\pi$ represents the tendency to repeat the same choice in general with values greater 1 reflecting perseveration and below 1 switching at the first stage. The same principle holds for $\rho$ weighting the variable resp(a), which was set to 1 if the same key was pressed in the preceding trial and 0 otherwise. Hence, $\pi$ and $\rho$ represent stickiness of choices and actions, respectively. The value of the free parameter $\omega$ indicates the relative weight of model-free and model-based values in the computation of choice probabilities and served as the outcome measure of interest in our study.

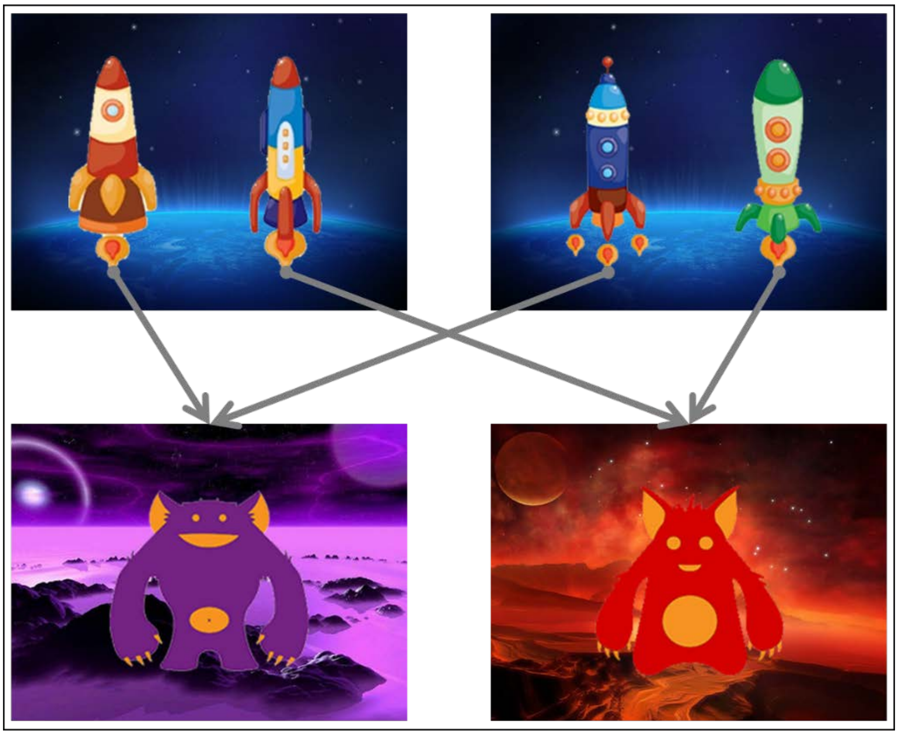

*Figure S5. Design of the sequential Markov decision task (2-Step; Kool et al., 2016). At the beginning of each trial, participants saw one of two first-stage states (upper panel). They chose one of two stimuli (rockets). Each rocket lead deterministically (grey arrows) to one of two second-stage states (aliens). Pressing a response button upon seeing the alien produced that trial's outcome (varying amounts of space treasure or antimatter), followed by the inter-trial interval.*

## 3. Supplementary Analyses

### 3.1 Reward Pairs task

#### *3.1.1 Choice data*

**Training sessions.** In accordance with the registered criteria, one participant was excluded from analyses because they had more than 50% missing trials in at least one training session. Three additional participants were excluded for choosing the worse of two stimuli during one training or the test session in at least 50% of trials. Another three participants were excluded for showing less than 50% accuracy in the debriefing questions about stimulus-reward associations.

Across all training sessions, a participant chose the more valuable stimulus on average in 78.90% of trials (*Mdn*=79.25%, *SD*=5.81%, range: 61.50% - 93.50%) with an increase of the average accuracy per session from 68.45% in the first session (*Mdn*=68.75%, SD=7.31%, range: 50.00% - 90.63%) to 83.62% in the last session (*Mdn*=84.38%, *SD*=6.37%, range: 61.88% - 95.00%; Figure S6A). In the crucial test phase after the fifth training session, participants chose the more valuable stimulus on average in 85.87% of trials (*Mdn*=87.00%, *SD*=6.76%, range: 63.00% - 97.00%). Thus, participants were generally able to perform the task according to the instructions and learned the stimulus-outcome associations. This was also evident in the debriefing questions asked after the test phase on the last assessment day, when participants indicated the reward value associated with the used stimuli (*n*=211 in this analysis, as two participants encountered technical errors during the debriefing questions at the end of the study). Most participants revealed (almost) perfect knowledge: 141 out of 211 participants (66.82%) indicated the correct reward level for all eight stimuli, 39 participants made one error, 20 participants made two errors, eight participants made three errors, and three participants made four errors. In total, participants reported the correct stimulus value on average for 93% of stimuli (*SD*=11%), in line with strong stimulus-value associations.

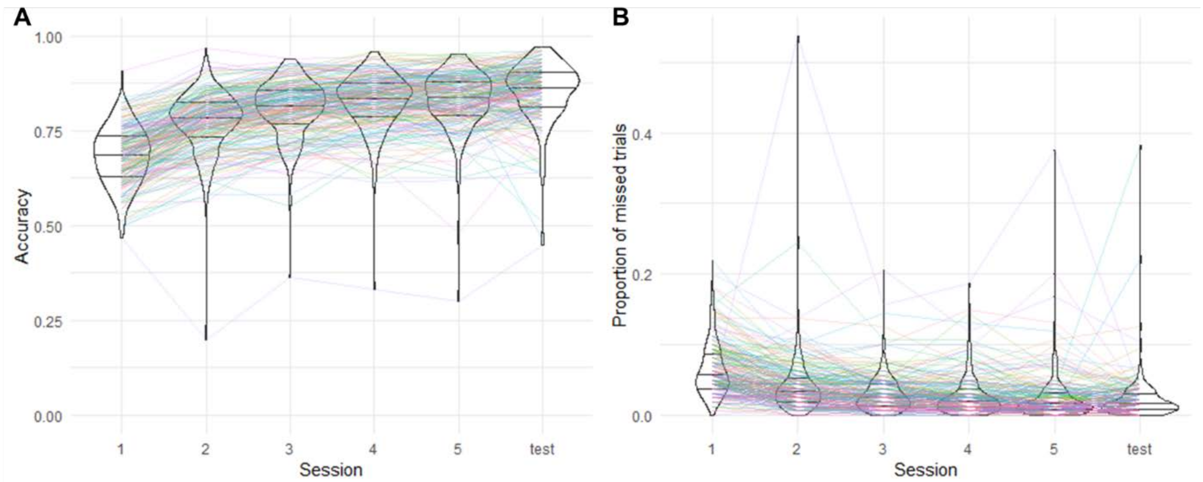


*Figure S6. Choice behavior during the training sessions (sessions 1-5) and the test phase of the Reward Pairs task. Combined violin and line plots displaying average accuracy (**A**) and proportion of missed trials (**B**) per participant and session. Each line represents one participant. The violins display the quartiles of the distributions. N=231 (displayed are all participants before applying the pre-registered exclusion criteria).*

### *3.1.2 Response times*

During both training and test phases, participants had to respond within 800ms from the onset of the stimuli. Across all training sessions, participants on average took 549.38ms to respond to the presented stimuli (*Mdn*=537.81ms, *SD*=94.14ms, range: 59.95ms – 800.00ms) with a slight decrease of the average RT from 565.97ms in the first session (*Mdn*=559.64ms, *SD*=103.20ms, range: 59.95.03ms - 799.91ms) to 532.86ms in the last session (*Mdn*=520.13ms, *SD*=88.66ms, range: 98.76ms - 799.89ms; Figure S7A). This decrease shows participants' growing familiarity and experience with the task. In the test phase, participants on average took 539.74ms to respond to the presented stimuli (*Mdn*=529.13ms, *SD*=92.98ms, range: 66.22ms – 799.96ms). Taken together with the relatively high accuracy, the observation of average response times being faster than the allowed maximum suggests that the task was easy enough for the participants, a prerequisite for habits to ensue.

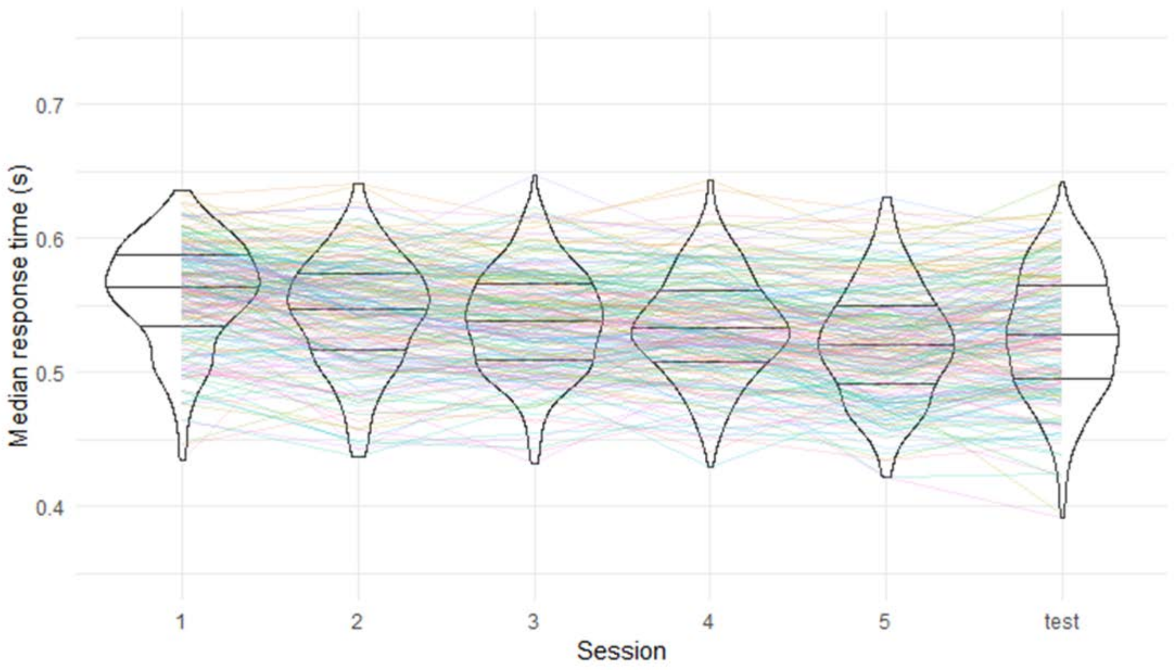


*Figure S7. Response times over the five training sessions (sessions 1-5) and the test phase of the Reward Pairs task. Displayed is the median RT per participant for each training session or test phase. Note the cut y-axis for better visibility. N=213.*

### *3.1.3 Ratings*

Participants rated two sets of 15 stimuli twice on a continuous visual analogue scale at the beginning of the first session by answering the question how much they liked this image on a scale from 0 to 100 with verbal anchor points “not at all” and “very much”, respectively. The eight individually most neutral stimuli of the first set and the four most neutral stimuli of the second set were used in the Reward Pairs and Unrewarded Habit task, respectively. All 30 stimuli were rated again at the end of the study. In addition, we included a set of ten stimuli in the ratings before and after the experiment. These additional stimuli were not used in any experiment but served only to estimate how re-test reliable stimulus ratings were in general.

We calculated the pre-post rating correlations of the stimuli of the first set using repeated measures correlations (rmcorr package; Bakdash & Marusich, 2017), which take into account that each participant rated several stimuli without having to average the ratings per participant. Hence, the repeated measures correlation finds the slope that fits individual data best, when allowing for participant-specific intercepts. The repeated measures correlation of

the stimuli used in the experiment (eight out of 15) was $r_{rm}(1602)=.134$ (95% $CI$=[.086, .182], $p<.001$ (Figure S8). Differentiating the same reward pair stimuli into more or less frequently chosen stimuli  shows very similar pre-post correlations in both subsets (frequently chosen stimuli: $r_{rm}(457)=.144$, 95% $CI$=[.053, .232], $p=.002$; rarely chosen stimuli: $r_{rm}(457)=.164$, 95% $CI$=[.074, .252], $p<.001$). Interestingly, the repeated-measures correlation between ratings before and after the study was higher for the stimuli not used in the experiment (seven out of 15): $r_{rm}(1373)=.293$ (95% $CI$=[.244, .340], $p<.001$. Presumably, this difference reflects the association of the used stimuli but not of the not-used stimuli with reward.

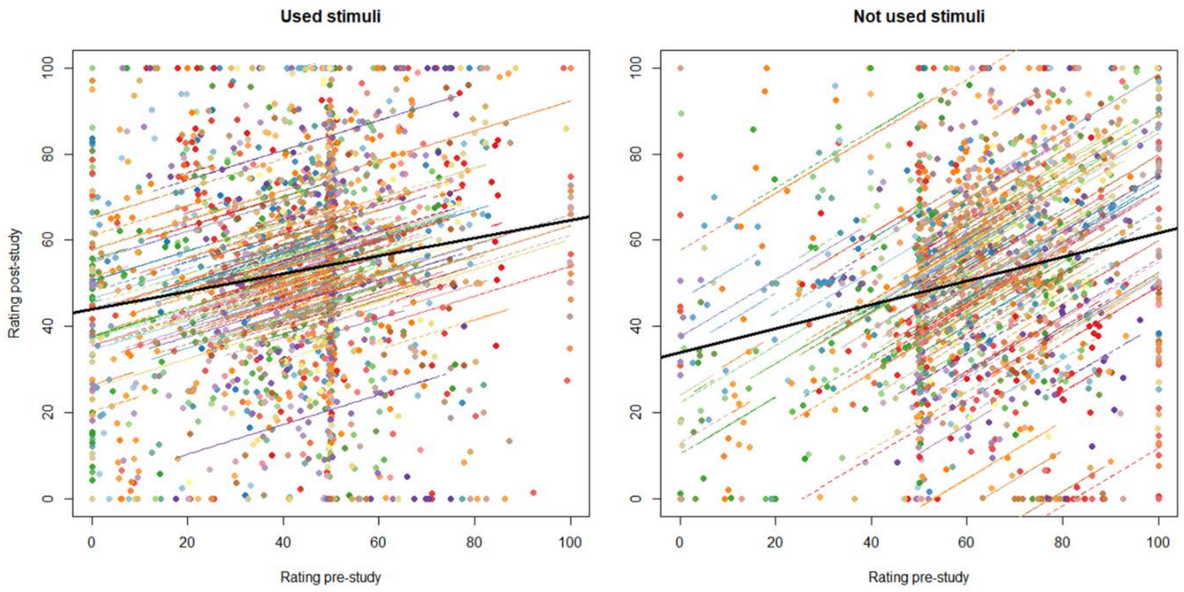


*Figure S8. Repeated-measures correlation plots for ratings of those eight geometric shapes used as stimuli in the Reward Pairs task on the left and of the seven shapes not used as stimuli on the right. Ratings at the start of the first session of the study are displayed on the x-axis; ratings after the end of the last session on the y-axis. The bold black line indicates the repeated-measures correlation over the whole group. Individual correlations are shown as colored lines. N=211.*

For comparison, the repeated-measures correlation for the additional set of ten stimuli, which were not used in any experiment was $r_{rm}(1970)=.622$, 95% $CI$=[.595, .649], $p<.001$. This correlation is numerically larger than the correlation of not used stimuli in the Reward Pairs task (see above) and similar to the correlation of not used stimuli in the Unrewarded Habits task (see below, 3.2.3).

## 3.2 Unrewarded Habit task

### *3.2.1 Choice data*

In accordance with the registered exclusion criteria, three participants were excluded from analyses because they had more than 50% missing trials in at least one training session. Four additional participants were excluded for not choosing the indicated stimulus in more than 50% of trials in at least one of the training sessions. Across all five training sessions, a participant chose the stimulus indicated to be chosen on average in 94.58% of trials (*SD*=5.16%, range: 51.62% - 100%) with virtually no change from 94.41% in the first session (*SD*=6.37%, range: 50.42% - 100%) to 94.10% in the last session (*SD*=4.92%, range: 59.58% - 100%; Figure S9A). This is to be expected in a task that does not require learning for correct performance.

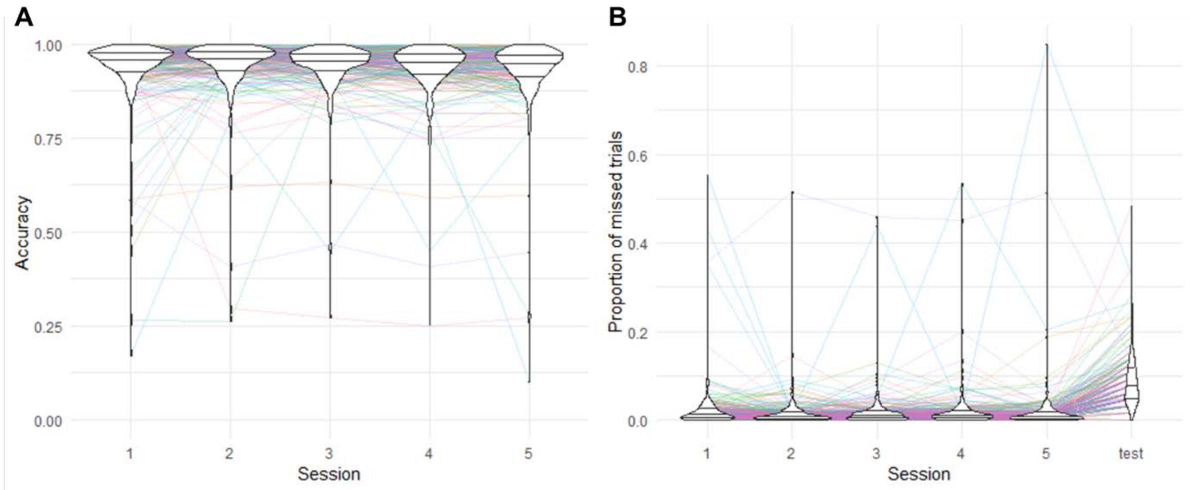


*Figure S9. Accuracy (**A**) and proportion of missed trials (**B**) per participant and session of the Unrewarded Habit task (training sessions 1-5 and test phase; as there are no correct choices in the test phase of this task, there is also no accuracy for the test phase). Each line represents the average of one participant. The violins display the quartiles of the distributions. N=231 (displayed are all participants before applying exclusion criteria).*

In an additional exploratory analysis, we have set up a generalized linear mixed effects model including a predictor of whether the chosen stimulus is in the same location as it was during training or not. This predictor did not have a statistically significant effect on choice proportions (*b*=0.075, OR=1.077, *p*=.588). However, the analysis yielded two additional interesting findings. First, including stimulus location of the chosen stimulus increased the

main effect of previous choice frequency (*b*=-0.257, OR=1.293, *p*=.001). As indicated by this change of the main effect, stimulus location also interacted statistically significantly with previous choice frequency (*b*=-0.140, OR=0.870, *p*=.037). This interaction implies that presenting stimuli in the same location as during training enhances the effect of previous choice frequency.

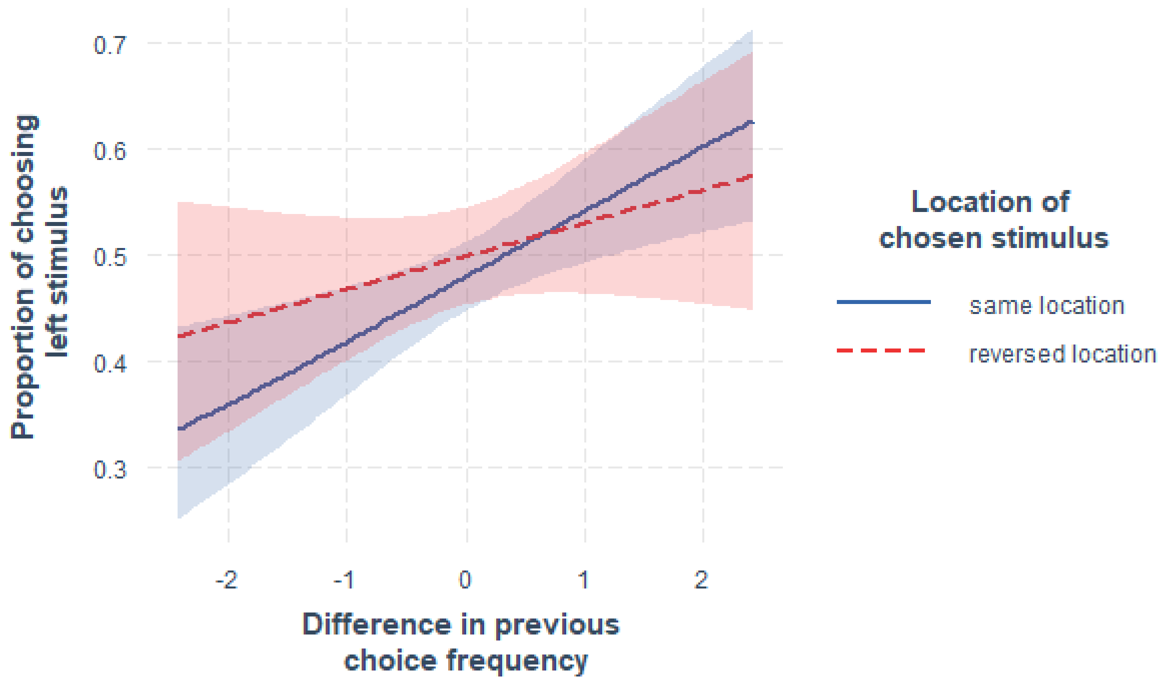


*Figure S10. . Interaction plot showing the effects of previous choice frequency (difference between left and right stimulus) and the location of the chosen stimulus depending on where it was presented during training (where stimulus presentation locations were not balanced but held constant). The difference in previous choice frequency was more positive (negative) if the left (right) stimulus had been chosen more frequently than the right (left) one during training. Thus, having chosen the left stimulus more frequently during training was associated with a higher proportion of choosing it again during test. This effect was more pronounced (i.e., the slope of the line was steeper) if the chosen stimulus was presented in the same location as during training.*

### ***3.2.2 Response times***

Across all training sessions, a participant on average took 276.99ms to respond to the presented stimuli (*Mdn*=274.65ms, *SD*=56.16ms, range: 50.00ms – 499.97ms) with an expected decrease of the average RT per session from 287.29ms in the first session (*Mdn*=284.58ms, *SD*=57.39ms, range: 50.10ms - 499.97ms) to 271.03ms in the last session (*Mdn*=269.49ms, *SD*=56.68ms, range: 50.15ms - 499.94ms; Figure S11). Thus as training

progressed, participants responded more quickly. By contrast, they responded more slowly during the test phase, in which choice was not instructed anymore but free: m=344.99ms, *Mdn*=349.01ms, *SD*=76.73ms, range: 51.16-499.99ms.

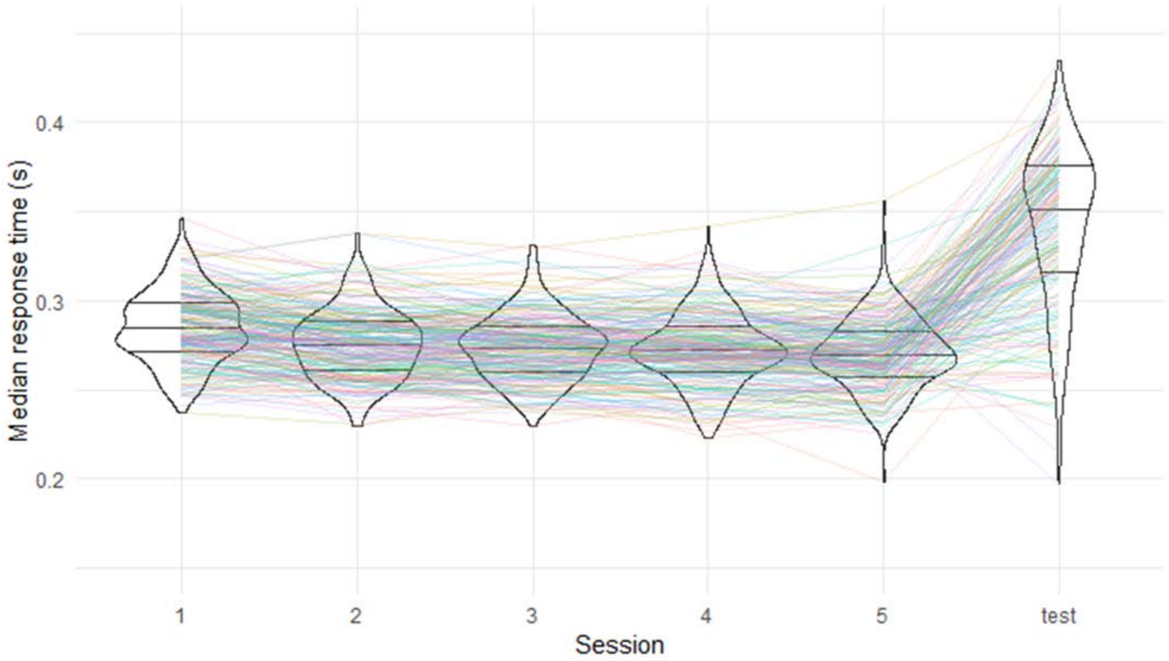


*Figure S11. Response times over the five training sessions (sessions 1-5) and the test phase of the Unrewarded Habit task. Displayed is the median RT per participant for each training session and for the test phase. Note the cut y-axis for better visibility. N=213.*

### *3.2.3 Ratings*

Participants rated the full set of used and unused stimuli before and after the study. The repeated measures correlation of the used stimuli (four out of 15) was $r_{rm}(683)=.328$ (95% *CI*=[.260, .394], *p*<.001; Figure S12). The correlation of pre-post ratings for the stimulus chosen frequently during training was positive (Spearman's $\rho$=.364, *p*<.001; Pearson's *r*(226)=.395, 95% *CI*=[.279, .499] , *p*<.001) and numerically slightly higher than the pre-post correlation of the rarely chosen stimulus (Spearman's $\rho$=.292, *p*<.001; Pearson's *r*(226)=.303, 95% *CI*=[.180, .417] , *p*<.001). The repeated measures correlation of the unused stimuli (eleven out of 15) was again higher than the one for used stimuli with $r_{rm}(2279)=.678$, 95% *CI*=[.655, .700], *p*<.001). These results indicate that there were larger changes in the rating of used and frequently chosen stimuli compared to unused and rarely chosen stimuli, which is consistent with our expectations in the main study.

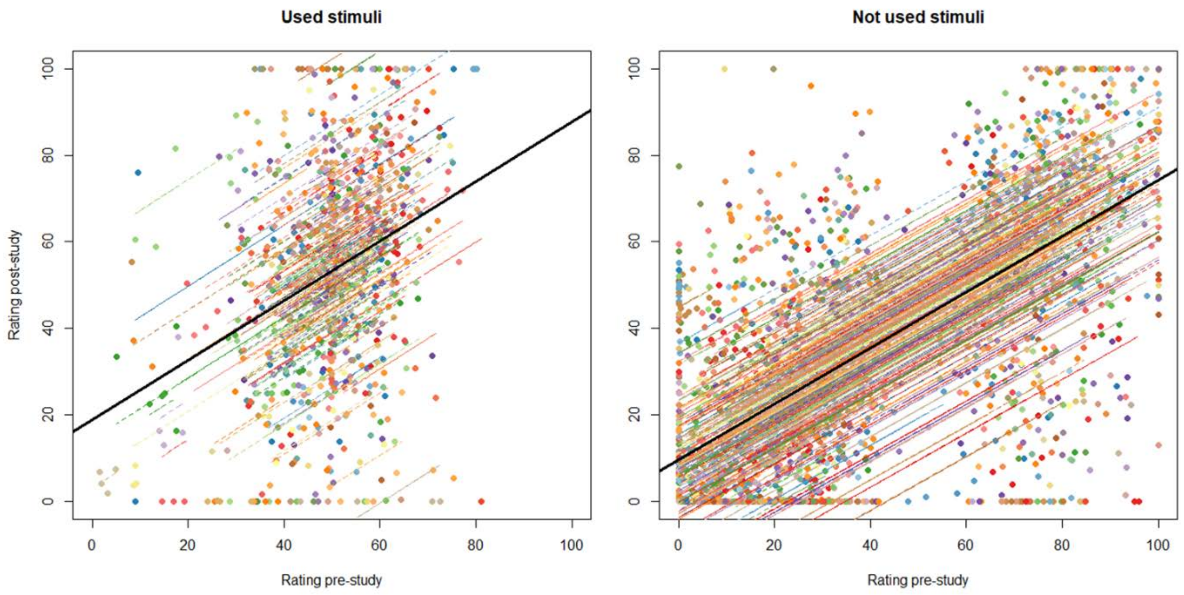


*Figure S12. Repeated-measures correlation plots for ratings of those four abstract images used as stimuli in the Unrewarded Habits task on the left and of the eleven images not used as stimuli on the right. Ratings at the start of the first session of the study are displayed on the x-axis; ratings after the end of the last session on the y-axis. The bold black line indicates the repeated-measures correlation over the whole group. Individual correlations are shown as colored lines. N=211.*

## 3.3 Outcome Devaluation task (Tricomi/Pool)

The outcome devaluation task by Tricomi and colleagues worked similarly as reported previously (Pool et al., 2022; Tricomi et al., 2009). Participants showed no decrease in responses per second for either the snack item devalued by satiation or for the still valued snack item (Figure S13A and B). However, the devaluation procedure worked as intended, because hunger decreased from before the task to after the devaluation procedure (Figure S13C) and the value of the devalued snack decreased more strongly than the one not devalued (Figure S13D and E). The amount eaten during devaluation did not have an effect on the change of behavior from before to after devaluation. Thus, participants showed the expected decrease in self-reported value through specific satiation, but they did not adapt their response rate for the devalued snack. This insensitivity to devaluation is usually interpreted as a sign of habitual behavior.

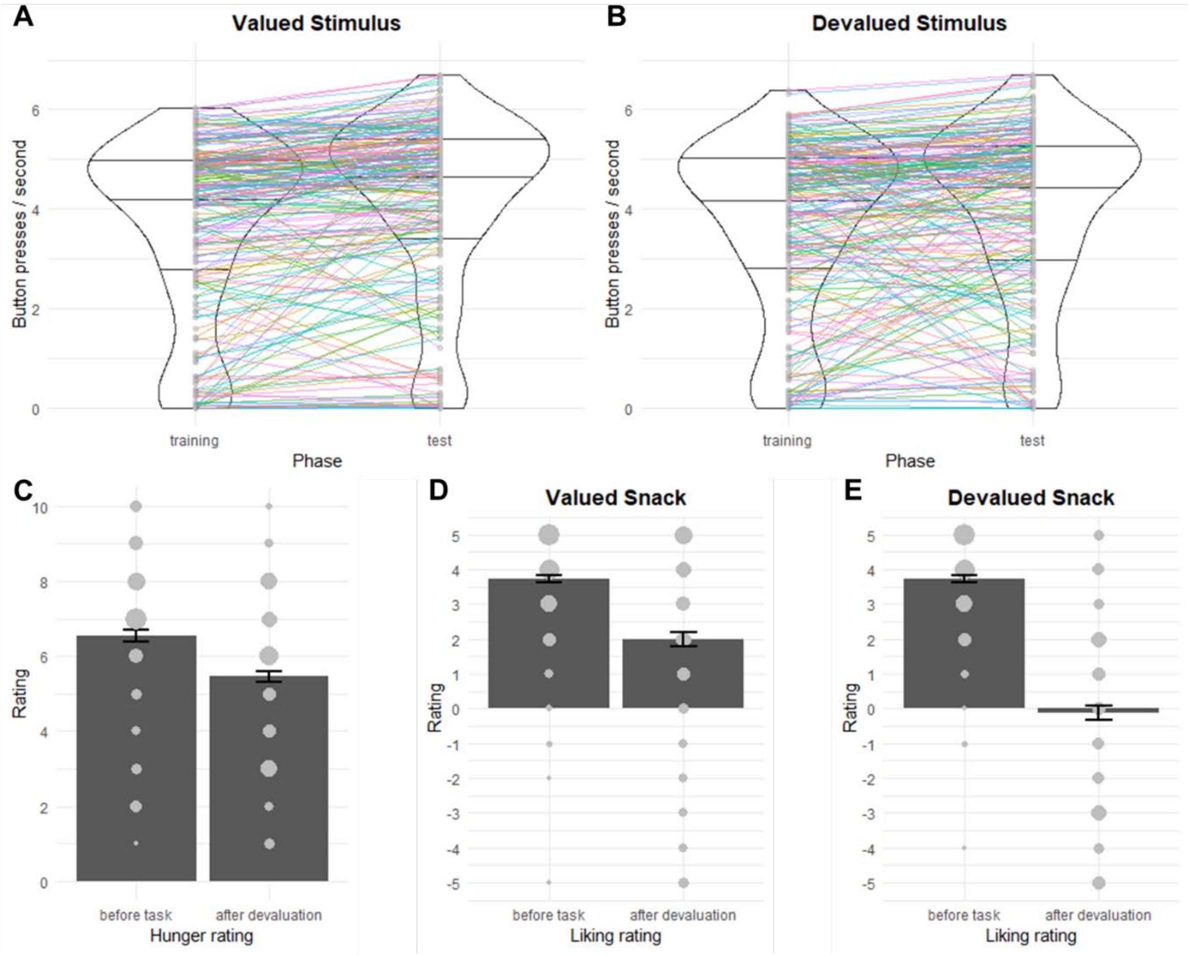


*Figure S13. Average button presses per second during the last half of the training session and the test phase are shown for each participant for the not devalued snack item (**A**) and for the snack item devalued by specific satiation (**B**; compare with Fig. 2 of Tricomi, Balleine, & O'Doherty, 2009, and Fig. 3 of Pool et al., 2022). (**C**) Hunger ratings before the task and after the devaluation procedure show an expected decrease. The liking ratings of the not devalued snack item (**D**) decreased less than those of the devalued snack item (**E**). Error bars depict standard errors. N=220.*

### 3.4 Outcome Devaluation task (Luque)

The outcome devaluation task by Luque and colleagues (2020) worked similarly as in their original study. Participants were mostly capable of switching their responses away from the devalued stimulus to the still valuable one during the devaluation blocks, both in the first session of training and in the last one (Figure S14A). However, the response times during those successful response switches were longer during the last than the first session (Figure S14B). These increased response times were previously interpreted as switch costs for overcoming a habitual choice tendency after devaluation (Luque et al., 2020).

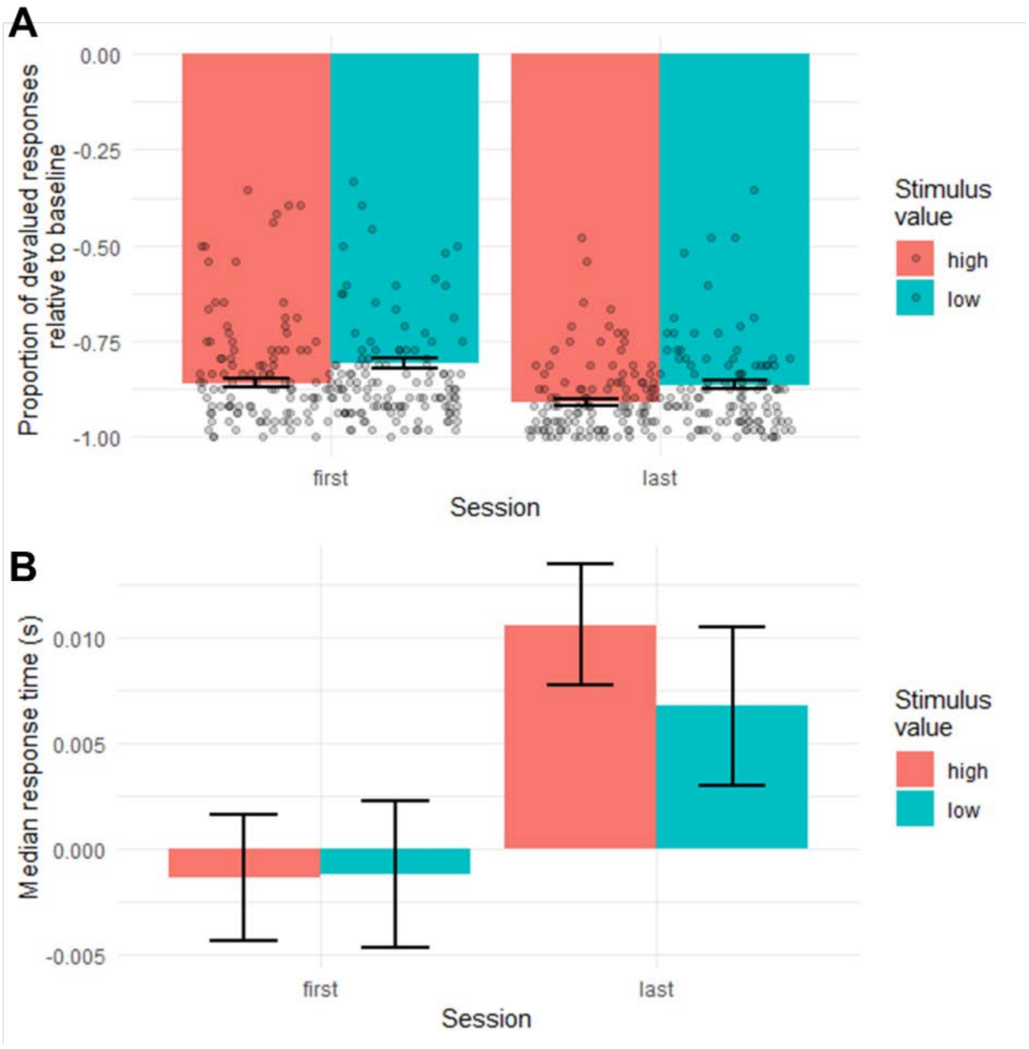


*Figure S14. Behavior in the Luque task. (**A**) Proportion of suboptimal responses during the devalued blocks in the first session participants performed the task (i.e., session 2 of our study) and in the last of the four sessions (i.e., session 5 of our study) for the high (red) and low value (blue) stimulus relative to baseline. A value of -1 corresponded to perfect switching away from the devalued to the still-valued stimulus, while a value of 0 reflected habitually selecting the devalued stimulus in all corresponding trials (compare to Fig. 2E and H in Luque et al., 2020). (**B**) Median response times for those trials, in which participants successfully switched away from the devalued to the still-valued stimulus during devaluation blocks, that is, switch costs (compare to Fig. 2F and I in Luque et al., 2020). While overt response rates did not change over the course of training, switch costs increased from the first to the last session. Error bars depict standard errors. N=114.*

### 3.5 Contingency Degradation task (Vaghi)

In the contingency degradation task by Vaghi and colleagues (2019), our participants showed behavior very similar to that of the original study's control group. Participants largely adapted their behavior during blocks with a degraded contingency between actions and outcomes compared to non-degraded blocks. This was evident in similar proportion during the first and last session of training (Figure S15A and B) with virtually no change over the course of four

training sessions (Figure S15C). Thus, participants in this task failed to show the insensitivity to a contingency degradation that would have been interpreted as habitual behavior and showed goal-directed, contingency-driven behavioral adaptation instead. This was also evident in participants' strong positive association between the actual difference in probabilities of outcomes given an action and no action (i.e., the contingency between actions and outcomes) and their button presses per second (repeated-measures correlation  across all four sessions: $r_{rm}(4373)=.661$, 95% $CI$=[.644, 677], $p<.001$) and between participants' block-wise rating of the causality between action and outcome and their response rate per second (repeated-measures correlation  across all four sessions: $r_{rm}(4758)=.753$, 95% $CI$=[.740, .765], $p<.001$).

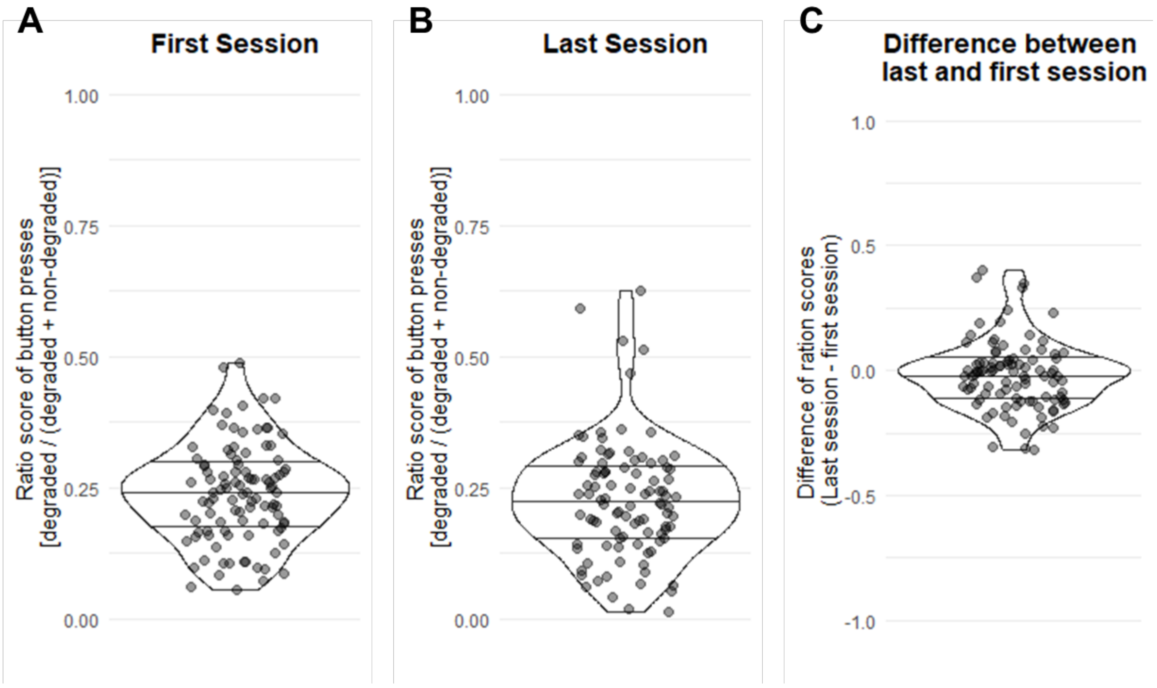


*Figure S15. Ratio score of button presses per second for blocks, in which the contingency between action and outcome was degraded or not degraded for the first (**A**) and last (**B**) of the four training sessions. Values near 0 corresponded to very few or no responses during degraded compared to non-degraded blocks; a value of 0.5 indicated as many responses during degraded as during non-degraded blocks; and values close to 1 reflected many more responses during degraded than non-degraded blocks. Participants in our study in general showed a decrease in responding during degraded compared to non-degraded blocks (compare to Fig. 4 in Vaghi et al. 2019; note that we reversed the scoring compared to Vaghi and colleagues). (**C**) Difference of ratio scores for the first and last session of training indicating an average change of 0 over the whole group of participants. N=97.*

### 3.6 Sequential Markov Decision Task (2-Step; Kool)

Participants in our study performed a modified version of Daw and colleagues' (2011) sequential Markov decision-making task (Kool et al., 2016). As in the previous reports, participants showed a strong tendency to repeat behavior that was rewarded before and used their knowledge of the task structure (i.e., the transition structure between first- and second-stage states) to increase their payout (Figure S16). This main effect of reward (i.e., an increased probability of repeating previously rewarded behavior irrespective of whether the first-stage state was the same as or different than in the previous trial) is an indicator of model-based control in this version of the 2-Step task. By contrast, an interaction effect of previous outcome and start state (i.e., an increased probability of repeating previously rewarded behavior when the start state was the same rather than different to the one in the previous trial) is an indicator of model-free control. Our participants descriptively showed both model-free and model-based influences on behavior similar to the original study's findings (Kool et al., 2016).

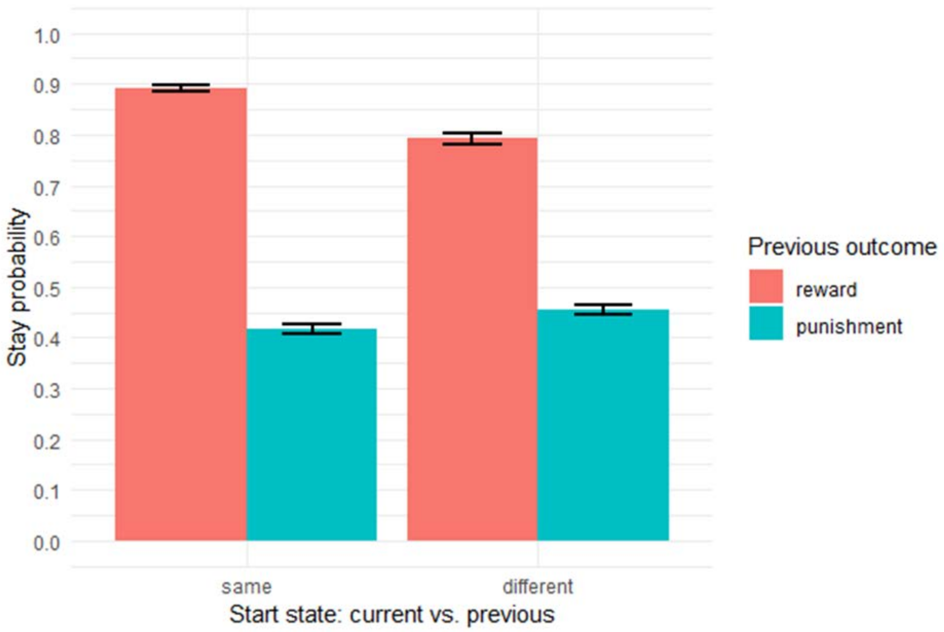


*Figure S16. The probability to repeat the same second-stage choice compared to the previous trial dependent on whether the first-stage state (i.e., the start state) of the current trial was the same or different than the one in the last trial and whether the previous trial's second-stage choice was rewarded or punished (compare to Fig. 16 in Kool, Cushman, & Gershman, 2016). Participants showed a higher probability of repeating previously rewarded behavior compared to punished behavior irrespective of the start state (which is a proxy for model-based control*

*in this task version), but this effect was numerically even stronger for the same start state (which corresponds to model-free behavior in this task version).*

### 3.7 Correlations between all task and questionnaire measures

In addition to the correlations between tasks within one modality (i.e., choices, response times, ratings, and computational parameters), we explored correlations between the habit tasks across different modalities (Table S6, Table S7). We did not find more substantial associations between the tasks than reported in the analyses of RQ3 in the main manuscript (see Table 2). We further explored correlations between the three habit questionnaires (Table S8) and correlations of anxiety and chronic stress with all habit tasks and questionnaires (Table S9, Table S10, and Table S11.

*Table S6. Correlations between Reward Pairs task, Unrewarded Habits task and all other tasks.*

| | | Reward Pairs | | | | | | | Unrewarded Habits | | | | |
|---|---|---|---|---|---|---|---|---|---|---|---|---|---|
| | | Choice | ΔChoice | RT | ΔRT | Rating | Comp. ($\alpha_{CK}$) | Comp. ($\beta_{CK}$) | Choice | ΔChoice | RT | ΔRT | Rating |
| **Reward Pairs** | ΔChoice | .65<br>[ .57; .72] | 1 | | | | | | | | | | |
| | RT | .37<br>[ .24; .49] | .18<br>[ .03; .33] | 1 | | | | | | | | | |
| | ΔRT | .15<br>[ .02; .27] | .22<br>[ .09; .35] | .43<br>[ .31; .53] | 1 | | | | | | | | |
| | Rating | .16<br>[ .03; .29] | .02<br>[-.14; .19] | .01<br>[-.13; .14] | -.07<br>[-.21; .07] | 1 | | | | | | | |
| | Comp. ($\alpha_{CK}$) | .04<br>[-.09; .17] | -.04<br>[-.19; .10] | .05<br>[-.10; .19] | -.02<br>[-.16; .13] | -.07<br>[-.22; .09] | 1 | | | | | | |
| | Comp. ($\beta_{CK}$) | -.01<br>[-.14; .12] | -.09<br>[-.21; .03] | -.08<br>[-.22; .06] | .04<br>[-.10; .18] | -.03<br>[-.16; .11] | -.30<br>[-.41; -.18] | 1 | | | | | |
| **Unrewarded Habits** | Choice | -.09<br>[-.22; .05] | -.03<br>[-.16; .11] | -.10<br>[-.24; .05] | -.04<br>[-.19; .11] | -.10<br>[-.24; .05] | .01<br>[-.14; .16] | .00<br>[-.13; .13] | 1 | | | | |
| | ΔChoice | -.09<br>[-.22; .06] | -.03<br>[-.17; .11] | -.03<br>[-.17; .12] | .01<br>[-.13; .15] | -.09<br>[-.22; .04] | .05<br>[-.08; .19] | -.07<br>[-.19; .05] | .74<br>[ .68; .80] | 1 | | | |
| | RT | .06<br>[-.07; .17] | .02<br>[-.11; .15] | .10<br>[-.04; .22] | .18<br>[ .02; .31] | .11<br>[-.06; .26] | -.02<br>[-.15; .11] | -.00<br>[-.13; .13] | .02<br>[-.13; .16] | .10<br>[-.04; .23] | 1 | | |
| | ΔRT | .13<br>[-.00; .25] | .07<br>[-.07; .21] | .03<br>[-.09; .15] | .08<br>[-.08; .23] | .14<br>[-.02; .29] | .01<br>[-.14; .16] | -.04<br>[-.17; .09] | .10<br>[-.06; .26] | .08<br>[-.09; .25] | .58<br>[ .46; .67] | 1 | |
| | Rating | -.09<br>[-.22; .04] | -.03<br>[-.15; .10] | -.05<br>[-.19; .09] | -.09<br>[-.23; .05] | -.15<br>[-.31; .02] | .02<br>[-.13; .17] | -.01<br>[-.13; .12] | .51<br>[ .41; .60] | .36<br>[ .25; .47] | -.14<br>[-.28; .01] | -.04<br>[-.18; .10] | 1 |
| **Luque** | Choice | .06<br>[-.12; .24] | .06<br>[-.11; .23] | -.04<br>[-.20; .13] | .01<br>[-.14; .17] | .08<br>[-.15; .30] | -.09<br>[-.24; .09] | .14<br>[-.05; .31] | -.09<br>[-.25; .06] | -.07<br>[-.22; .07] | -.07<br>[-.24; .10] | .02<br>[-.15; .19] | -.13<br>[-.30; .04] |
| | ΔChoice | -.07<br>[-.25; .10] | .02<br>[-.16; .21] | -.04<br>[-.21; .12] | -.05<br>[-.22; .13] | -.08<br>[-.28; .12] | -.01<br>[-.19; .15] | -.03<br>[-.18; .13] | -.07<br>[-.23; .09] | -.08<br>[-.22; .07] | -.13<br>[-.29; .04] | -.13<br>[-.30; .03] | -.15<br>[-.32; .02] |
| | RT | -.15<br>[-.35; .06] | -.11<br>[-.29; .09] | .01<br>[-.16; .16] | .07<br>[-.12; .24] | -.02<br>[-.23; .18] | .01<br>[-.15; .17] | -.04<br>[-.23; .14] | -.17<br>[-.35; .01] | -.12<br>[-.29; .06] | .20<br>[ .03; .37] | .04<br>[-.13; .22] | -.23<br>[-.42; -.03] |
| | ΔRT | -.13<br>[-.32; .05] | -.07<br>[-.25; .12] | .00<br>[-.18; .17] | .03<br>[-.14; .19] | -.09<br>[-.29; .10] | .01<br>[-.17; .18] | -.07<br>[-.26; .12] | -.10<br>[-.28; .07] | -.06<br>[-.23; .11] | .10<br>[-.04; .24] | .04<br>[-.12; .20] | -.10<br>[-.31; .12] |
| **Tricomi** | Choice | .08<br>[-.06; .22] | .15<br>[-.01; .30] | -.02<br>[-.16; .11] | .11<br>[-.04; .25] | -.03<br>[-.15; .10] | .01<br>[-.12; .15] | .08<br>[-.03; .20] | .07<br>[-.08; .21] | .07<br>[-.07; .20] | -.05<br>[-.17; .07] | -.04<br>[-.20; .13] | .03<br>[-.10; .15] |
| **Vaghi** | Choice | -.05<br>[-.23; .13] | -.06<br>[-.22; .09] | -.14<br>[-.34; .05] | -.07<br>[-.25; .11] | -.03<br>[-.20; .16] | .04<br>[-.15; .24] | -.03<br>[-.20; .14] | .04<br>[-.17; .23] | .00<br>[-.20; .19] | .07<br>[-.16; .28] | .10<br>[-.08; .28] | -.09<br>[-.28; .12] |
| | ΔChoice | .04<br>[-.13; .21] | .02<br>[-.15; .18] | .03<br>[-.16; .22] | -.00<br>[-.18; .18] | .08<br>[-.09; .25] | .10<br>[-.10; .28] | .03<br>[-.15; .20] | .05<br>[-.14; .24] | .01<br>[-.18; .19] | .06<br>[-.14; .29] | .07<br>[-.11; .24] | -.04<br>[-.22; .17] |
| **Kool** | Comp. | .03<br>[-.11; .17] | .08<br>[-.05; .21] | -.15<br>[-.29; .00] | .04<br>[-.10; .17] | .10<br>[-.04; .23] | -.10<br>[-.22; .02] | .08<br>[-.07; .22] | -.13<br>[-.26; .00] | -.16<br>[-.30; -.02] | .04<br>[-.11; .18] | -.00<br>[-.14; .13] | -.14<br>[-.28; .01] |

$\alpha_{CK}$, learning rate of Choice Kernel; $\beta_{CK}$, weight of Choice Kernel values; ΔChoice / ΔRT, difference in choice scores / RT scores between pretest (1st session) and test (last session) ; Comp, computational parameter; RT, response time.

*Table S7. Correlations between previously used tasks.*

| | | Luque | | | | Tricomi | Vaghi | |
|---|---|---|---|---|---|---|---|---|
| | | Choice | ΔChoice | RT | ΔRT | Choice | Choice | ΔChoice |
| **Luque** | Choice | 1 | | | | | | |
| | ΔChoice | .39 [ .15; .56] | 1 | | | | | |
| | RT | .19 [-.02; .40] | .09 [-.11; .29] | 1 | | | | |
| | ΔRT | .16 [-.06; .35] | .15 [-.08; .36] | .64 [ .49; .76] | 1 | | | |
| **Tricomi** | Choice | .07 [-.10; .25] | .12 [-.07; .29] | .04 [-.12; .21] | .09 [-.08; .25] | 1 | | |
| **Vaghi** | Choice | .00 [-.29; .31] | -.02 [-.22; .18] | .03 [-.37; .60] | .02 [-.23; .35] | -.08 [-.29; .13] | 1 | |
| | ΔChoice | -.04 [-.28; .25] | -.05 [-.24; .14] | .01 [-.31; .39] | .02 [-.21; .29] | -.14 [-.31; .07] | .67 [ .52; .78] | 1 |
| **Kool** | Comp. | .17 [-.03; .35] | .02 [-.17; .20] | -.00 [-.18; .19] | -.02 [-.20; .17] | -.05 [-.17; .07] | -.02 [-.21; .17] | -.02 [-.22; .17] |

ΔChoice / ΔRT, difference in choice scores / RT scores between pretest (1st session) and test (last session); RT, response time.

*Table S8. Correlations between habit questionnaires.*

| | | COHS | | HTQ | | | |
|---|---|---|---|---|---|---|---|
| | | automaticity | routine | total score | compulsivity | regularity | aversion to novelty |
| **COHS** | automaticity | 1 | | | | | |
| | routine | .10 [-.04; .24] | 1 | | | | |
| **HTQ** | total score | .21 [ .09; .32] | .52 [ .39; .63] | 1 | | | |
| | compulsivity | .26 [ .15; .37] | .20 [ .05; .33] | .65 [ .56; .72] | 1 | | |
| | regularity | .17 [ .03; .30] | .56 [ .45; .66] | .65 [ .56; .72] | .24 [ .12; .36] | 1 | |
| | aversion to novelty | .00 [-.13; .13] | .30 [ .16; .42] | .66 [ .58; .73] | .05 [-.09; .18] | .13 [-.02; .28] | 1 |
| **SRHI** | | .03 [-.13; .18] | .30 [ .18; .42] | .20 [ .07; .32] | .07 [-.06; .19] | .23 [ .09; .36] | .11 [-.03; .25] |

COHS, Creature of Habit Scale; HTQ, Habitual tendencies Questionnaire; SRHI, Self-Report Habit Index.

*Table S9. Correlations of anxiety and chronic stress with measures of the Reward Pairs und Unrewarded Habits tasks.*

| | | Reward Pairs | | | | | | | Unrewarded Habits | | | | |
|---|---|---|---|---|---|---|---|---|---|---|---|---|---|
| | | Choice | ΔChoice | RT | ΔRT | Rating | Comp. ($\alpha_{CK}$) | Comp. ($\beta_{CK}$) | Choice | ΔChoice | RT | ΔRT | Rating |
| **STAI** | | -.04 [-.17; .10] | -.02 [-.17; .12] | .02 [-.10; .15] | -.06 [-.20; .07] | .04 [-.10; .18] | .01 [-.15; .17] | -.04 [-.17; .10] | .04 [-.09; .18] | .05 [-.08; .18] | .02 [-.13; .15] | -.06 [-.20; .08] | -.14 [-.28; .01] |
| **TICS** | work overload | .05 [-.08; .18] | -.00 [-.14; .14] | .03 [-.10; .16] | -.07 [-.22; .07] | .12 [-.03; .26] | -.03 [-.21; .14] | .06 [-.07; .20] | -.07 [-.21; .08] | -.04 [-.18; .12] | -.01 [-.16; .13] | -.04 [-.17; .09] | -.16 [-.30; -.02] |
| | social overload | .11 [-.02; .23] | .13 [-.01; .26] | .13 [ .01; .25] | .00 [-.14; .14] | .05 [-.10; .19] | -.09 [-.23; .06] | .06 [-.07; .19] | -.11 [-.24; .03] | -.09 [-.24; .06] | -.06 [-.20; .08] | -.09 [-.22; .05] | -.23 [-.37; -.10] |
| | pressure to perform | .07 [-.06; .20] | .02 [-.10; .14] | .07 [-.06; .20] | -.07 [-.22; .09] | .09 [-.05; .21] | -.06 [-.20; .09] | .07 [-.07; .20] | -.12 [-.24; .01] | -.08 [-.21; .05] | .02 [-.15; .18] | -.01 [-.17; .14] | -.20 [-.34; -.06] |
| | work discontent | .11 [-.02; .23] | .13 [ .01; .26] | .01 [-.12; .13] | -.01 [-.16; .15] | .04 [-.11; .18] | -.02 [-.18; .15] | .03 [-.09; .15] | -.07 [-.20; .06] | -.08 [-.21; .06] | -.02 [-.16; .13] | -.05 [-.20; .10] | -.22 [-.36; -.06] |
| | excessive demands at work | .05 [-.07; .17] | .00 [-.13; .13] | .14 [ .01; .27] | .02 [-.13; .18] | .03 [-.10; .17] | -.03 [-.18; .12] | -.05 [-.16; .07] | .01 [-.13; .16] | .03 [-.11; .16] | .14 [ .00; .27] | .04 [-.10; .18] | -.09 [-.24; .06] |
| | lack of social recognition | .10 [-.03; .22] | .09 [-.05; .22] | .01 [-.12; .15] | -.02 [-.17; .12] | .05 [-.10; .20] | .07 [-.09; .23] | .01 [-.12; .13] | .03 [-.10; .17] | -.03 [-.17; .11] | -.05 [-.21; .10] | -.12 [-.26; .02] | -.11 [-.24; .02] |
| | social tension | .05 [-.09; .19] | .18 [ .06; .31] | -.03 [-.17; .11] | .03 [-.11; .18] | .00 [-.16; .17] | .05 [-.11; .21] | .06 [-.06; .19] | -.00 [-.15; .15] | -.06 [-.21; .09] | -.05 [-.20; .09] | -.07 [-.21; .08] | -.07 [-.22; .08] |
| | social isolation | .04 [-.08; .17] | .04 [-.10; .17] | -.02 [-.15; .11] | -.07 [-.22; .08] | .03 [-.12; .16] | -.02 [-.15; .11] | .06 [-.07; .18] | .01 [-.14; .15] | .02 [-.11; .15] | .12 [-.03; .27] | .11 [-.03; .25] | -.08 [-.24; .07] |
| | chronic worrying | -.00 [-.13; .13] | -.00 [-.15; .14] | .08 [-.05; .21] | .00 [-.14; .15] | .04 [-.10; .18] | .00 [-.15; .16] | .08 [-.04; .21] | .06 [-.08; .20] | .08 [-.05; .22] | .06 [-.08; .19] | -.09 [-.23; .04] | -.09 [-.23; .04] |

$\alpha_{CK}$, learning rate of Choice Kernel; $\beta_{CK}$, weight of Choice Kernel values; ΔChoice / ΔRT, difference in choice scores / RT scores between pretest (1st session) and test (last session); Comp, computational parameter; RT, response time; STAI, State/Trait Anxiety Inventory; TICS, Trier Inventory for Chronic Stress.

*Table S10. Correlations of anxiety and chronic stress with measures of the Outcome Devaluation, Contingency Degradation and Sequential Choice tasks.*

| | | Luque | | | | Tricomi | Vaghi | | Kool |
|---|---|---|---|---|---|---|---|---|---|
| | | Choice | ΔChoice | RT | ΔRT | Choice | Choice | ΔChoice | Comp. |
| **STAI** | | -.02 [-.18; .16] | .07 [-.12; .25] | .01 [-.17; .19] | .03 [-.15; .20] | -.00 [-.12; .13] | -.11 [-.28; .09] | .08 [-.11; .27] | .06 [-.07; .19] |
| **TICS** | work overload | .15 [-.02; .32] | .13 [-.06; .30] | -.06 [-.22; .11] | -.13 [-.30; .06] | .07 [-.05; .18] | -.18 [-.33; .00] | -.09 [-.28; .12] | .10 [-.04; .24] |
| | social overload | -.03 [-.19; .13] | .03 [-.15; .21] | -.03 [-.21; .17] | -.05 [-.24; .13] | .01 [-.09; .12] | -.12 [-.28; .05] | .05 [-.13; .23] | .11 [-.02; .25] |
| | pressure to perform | .10 [-.08; .28] | .11 [-.06; .29] | .05 [-.13; .23] | .04 [-.14; .22] | -.03 [-.15; .10] | -.03 [-.20; .15] | .05 [-.13; .24] | .08 [-.06; .22] |
| | work discontent | -.05 [-.21; .13] | .05 [-.15; .23] | .18 [-.00; .38] | .21 [ .00; .40] | .05 [-.08; .18] | -.06 [-.24; .17] | .18 [-.00; .37] | .06 [-.08; .21] |
| | excessive demands at work | .01 [-.16; .18] | .01 [-.16; .19] | -.04 [-.20; .13] | -.06 [-.22; .10] | -.03 [-.16; .11] | -.14 [-.31; .04] | .04 [-.14; .24] | .03 [-.10; .15] |
| | lack of social recognition | -.01 [-.19; .16] | .09 [-.11; .28] | .04 [-.14; .21] | .01 [-.18; .21] | .07 [-.06; .21] | -.02 [-.20; .17] | .07 [-.11; .26] | .02 [-.12; .16] |
| | social tension | -.00 [-.18; .18] | .06 [-.12; .25] | -.04 [-.23; .16] | .05 [-.17; .25] | .05 [-.07; .18] | -.07 [-.23; .10] | .11 [-.07; .29] | .11 [-.02; .23] |
| | social isolation | .06 [-.11; .22] | .02 [-.15; .20] | .13 [-.05; .30] | .04 [-.14; .21] | -.03 [-.17; .13] | .03 [-.16; .23] | -.00 [-.21; .19] | .03 [-.09; .16] |
| | chronic worrying | .03 [-.13; .22] | .10 [-.07; .28] | .02 [-.15; .19] | .07 [-.11; .25] | .03 [-.11; .17] | -.17 [-.33; .02] | -.07 [-.26; .13] | .07 [-.06; .21] |

ΔChoice / ΔRT, difference in choice scores / RT scores between pretest (1st session) and test (last session); RT, response time; STAI, State/Trait Anxiety Inventory; TICS, Trier Inventory for Chronic Stress.

*Table S11. Correlations of anxiety and chronic stress with habit questionnaires.*

| | | COHS | | HTQ | | | | SRHI |
|---|---|---|---|---|---|---|---|---|
| | | automaticity | routine | total score | compulsivity | regularity | aversion to novelty | |
| **STAI** | | .24 [ .11; .36] | .03 [-.13; .18] | .37 [ .24; .49] | .59 [ .50; .67] | .14 [ .01; .27] | .02 [-.12; .16] | -.13 [-.25; .01] |
| **TICS** | work overload | .28 [ .14; .41] | .06 [-.08; .19] | .27 [ .14; .38] | .35 [ .22; .47] | .24 [ .10; .37] | -.04 [-.16; .09] | .08 [-.05; .21] |
| | social overload | .30 [ .16; .43] | .14 [ .02; .26] | .18 [ .05; .30] | .22 [ .10; .34] | .19 [ .05; .33] | -.04 [-.18; .10] | .16 [ .03; .29] |
| | pressure to perform | .17 [ .03; .30] | .08 [-.07; .23] | .09 [-.04; .22] | .09 [-.04; .23] | .18 [ .04; .31] | -.07 [-.20; .06] | .13 [-.01; .27] |
| | work discontent | .25 [ .12; .38] | .14 [ .01; .26] | .35 [ .22; .46] | .38 [ .25; .50] | .27 [ .14; .39] | .06 [-.09; .20] | .08 [-.05; .20] |
| | excessive demands at work | .21 [ .08; .34] | -.00 [-.12; .12] | .28 [ .15; .40] | .42 [ .30; .53] | .21 [ .07; .33] | -.06 [-.19; .09] | -.00 [-.13; .13] |
| | lack of social recognition | .15 [ .01; .29] | .18 [ .03; .31] | .25 [ .12; .37] | .26 [ .14; .38] | .22 [ .07; .35] | .03 [-.11; .17] | .13 [ .00; .26] |
| | social tension | .12 [-.01; .25] | .10 [-.03; .23] | .10 [-.04; .23] | .20 [ .06; .32] | .19 [ .05; .33] | -.15 [-.28; -.02] | .09 [-.04; .22] |
| | social isolation | .05 [-.09; .19] | .02 [-.13; .15] | .30 [ .16; .43] | .35 [ .22; .47] | .19 [ .06; .32] | .06 [-.08; .20] | -.03 [-.16; .10] |
| | chronic worrying | .19 [ .06; .32] | .12 [-.03; .26] | .47 [ .35; .58] | .60 [ .50; .68] | .21 [ .08; .34] | .12 [-.02; .25] | -.05 [-.17; .06] |

COHS, Creature of Habit Scale; HTQ, Habitual tendencies Questionnaire; SRHI, Self-Report Habit Index; STAI, State/Trait Anxiety Inventory; TICS, Trier Inventory for Chronic Stress.

## 4. R Reproducibility

```
R version 4.2.0 (2022-04-22 ucrt)
Platform: x86_64-w64-mingw32/x64 (64-bit)
Running under: Windows 10 x64 (build 19044)

Matrix products: default

locale:
[1] LC_COLLATE=German_Switzerland.utf8  LC_CTYPE=German_Switzerland.utf8
[3] LC_MONETARY=German_Switzerland.utf8 LC_NUMERIC=C
[5] LC_TIME=German_Switzerland.utf8

attached base packages:
[1] parallel  stats     graphics  grDevices utils     datasets  methods
[8] base

other attached packages:
 [1] semTools_0.5-6     miceadds_3.16-18   mice_3.15.0
 [4] haven_2.5.1        openxlsx_4.2.5.1   sjlabelled_1.2.0
 [7] MBESS_4.9.2        lavaan_0.6-12      reghelper_1.1.1
[10] simr_1.0.6         boot_1.3-28        MCMCglmm_2.34
[13] ape_5.6-2          coda_0.19-4        scales_1.2.1
[16] optimx_2022-4.30   forcats_0.5.2      doParallel_1.0.17
[19] iterators_1.0.14   foreach_1.5.2      dfoptim_2020.10-1
[22] lmerTest_3.1-3     interactions_1.1.5 fitdistrplus_1.1-8
[25] survival_3.3-1     MASS_7.3-56        patchwork_1.1.2
[28] rmcorr_0.5.4       tibble_3.1.8       tidyr_1.2.1
[31] dplyr_1.0.10       psychTools_2.2.9   psych_2.2.9
[34] ggplot2_3.4.0      lme4_1.1-31        Matrix_1.5-3
[37] data.table_1.14.6  R.matlab_3.7.0     pacman_0.5.1

loaded via a namespace (and not attached):
 [1] nlme_3.1-157        pbkrtest_0.5.1      insight_0.18.8
 [4] numDeriv_2016.8-1.1 tensorA_0.36.2      tools_4.2.0
 [7] backports_1.4.1     utf8_1.2.2          R6_2.5.1
[10] DBI_1.1.3           mgcv_1.8-40         colorspace_2.0-3
[13] withr_2.5.0         tidyselect_1.2.0    mnormt_2.1.1
[16] emmeans_1.8.3       compiler_4.2.0      cli_3.5.0
[19] binom_1.1-1.1       mvtnorm_1.1-3       stringr_1.5.0
[22] digest_0.6.31       pbivnorm_0.6.0      foreign_0.8-82
[25] minqa_1.2.5         rmarkdown_2.19      R.utils_2.12.2
[28] pkgconfig_2.0.3     htmltools_0.5.4     plotrix_3.8-2
[31] fastmap_1.1.0       rlang_1.0.6         rstudioapi_0.14
[34] RLRsim_3.1-8        generics_0.1.3      zip_2.2.2
[37] car_3.1-1           R.oo_1.25.0         magrittr_2.0.3
[40] Rcpp_1.0.9          munsell_0.5.0       fansi_1.0.3
[43] abind_1.4-5         lifecycle_1.0.3     R.methodsS3_1.8.2
[46] stringi_1.7.8       yaml_2.3.6          carData_3.0-5
[49] plyr_1.8.8          grid_4.2.0          crayon_1.5.2
[52] lattice_0.20-45     splines_4.2.0       jtools_2.2.1
[55] pander_0.6.5        hms_1.1.2           knitr_1.41
[58] pillar_1.8.1        cubature_2.0.4.5    estimability_1.4.1
[61] corpcor_1.6.10      stats4_4.2.0        codetools_0.2-18
[64] glue_1.6.2          evaluate_0.19       mitools_2.4
[67] vctrs_0.5.1         nloptr_2.0.3        gtable_0.3.1
[70] purrr_1.0.0         xfun_0.36           xtable_1.8-4
[73] broom_1.0.2         ellipsis_0.3.2
```

## References

Bakdash, J. Z., & Marusich, L. R. (2017). Repeated Measures Correlation. *Frontiers in Psychology*, *8*. https://doi.org/10.3389/fpsyg.2017.00456

Cicchetti, D. V. (1994). Guidelines, Criteria, and Rules of Thumb for Evaluating Normed and Standardized Assessment Instruments in Psychology. *Psychological Assessment*, *6*(4), 284–290. https://doi.org/1040-3590/94/53.00

Daunizeau, J., Adam, V., & Rigoux, L. (2014). VBA: A Probabilistic Treatment of Nonlinear Models for Neurobiological and Behavioural Data. *PLOS Computational Biology*, *10*(1), e1003441. https://doi.org/10.1371/journal.pcbi.1003441

Daw, N. D., Gershman, S. J., Seymour, B., Dayan, P., & Dolan, R. J. (2011). Model-Based Influences on Humans' Choices and Striatal Prediction Errors. *Neuron*, *69*(6), 1204–1215. https://doi.org/10.1016/j.neuron.2011.02.027

Findling, C., Skvortsova, V., Dromnelle, R., Palminteri, S., & Wyart, V. (2019). Computational noise in reward-guided learning drives behavioral variability in volatile environments. *Nature Neuroscience*, *22*(12), 2066–2077. https://doi.org/10.1038/s41593-019-0518-9

Kool, W., Cushman, F. A., & Gershman, S. J. (2016). When Does Model-Based Control Pay Off? *PLOS Comput Biol*, *12*(8), e1005090. https://doi.org/10.1371/journal.pcbi.1005090

Kool, W., Gershman, S. J., & Cushman, F. A. (2017). Cost-Benefit Arbitration Between Multiple Reinforcement-Learning Systems. *Psychological Science*, *28*(9), 1321–1333. https://doi.org/10.1177/0956797617708288

Lance, C. E., Butts, M. M., & Michels, L. C. (2006). The Sources of Four Commonly Reported Cutoff Criteria: What Did They Really Say? *Organizational Research Methods*, *9*(2), 202–220. https://doi.org/10.1177/1094428105284919

Luque, D., Molinero, S., Watson, P., López, F. J., & Le Pelley, M. E. (2020). Measuring Habit Formation Through Goal-Directed Response Switching. *Journal of Experimental Psychology. General*, *149*(8), 1449–1459. https://doi.org/10.1037/xge0000722

Miller, K. J., Shenhav, A., & Ludvig, E. A. (2019). Habits without values. *Psychological Review*, *126*(2), 292–311. https://doi.org/10.1037/rev0000120

Otto, A. R., Raio, C. M., Chiang, A., Phelps, E. A., & Daw, N. D. (2013). Working-memory capacity protects model-based learning from stress. *Proceedings of the National Academy of Sciences*, *110*(52), 20941–20946. https://doi.org/10.1073/pnas.1312011110

Pool, E. R., Gera, R., Fransen, A., Perez, O. D., Cremer, A., Aleksic, M., Tanwisuth, S., Quail, S., Ceceli, A. O., Manfredi, D. A., Nave, G., Tricomi, E., Balleine, B., Schonberg, T., Schwabe, L., & O'Doherty, J. P. (2022). Determining the effects of training duration on the behavioral expression of habitual control in humans: A multilaboratory investigation. *Learning & Memory*, *29*(1), 16–28. https://doi.org/10.1101/lm.053413.121

Raftery, A. E. (1995). Bayesian Model Selection in Social Research. *Sociological Methodology*, *25*, 111–163. JSTOR. https://doi.org/10.2307/271063

Rescorla, R. A., & Wagner, A. R. (1972). A Theory of Pavlovian Conditioning: Variations in the Effectiveness of Reinforcement and Nonreinforcement. In A. H. Black & W. F. Prokasy (Eds.), *Classical conditioning II: current research and theory* (pp. 64–99). Appleton - Century - Crofts, Inc.

Rigoux, L., Stephan, K. E., Friston, K. J., & Daunizeau, J. (2014). Bayesian model selection for group studies—Revisited. *NeuroImage*, *84*, 971–985. https://doi.org/10.1016/j.neuroimage.2013.08.065

Rummery, G. A., & Niranjan, M. (1994). *On-line Q-learning using connectionist systems*.

Schwarz, G. E. (1978). Estimating the dimension of a model. *Annals of Statistics*, *6*(2), 461–464. https://doi.org/10.1214/aos/1176344136

Stephan, K. E., Penny, W. D., Daunizeau, J., Moran, R. J., & Friston, K. J. (2009). Bayesian model selection for group studies. *NeuroImage*, *46*(4), 1004–1017. https://doi.org/10.1016/j.neuroimage.2009.03.025

Sutton, R. S., & Barto, A. G. (1998). *Reinforcement learning—An introduction* (1st ed.). MIT Press.

Tricomi, E., Balleine, B. W., & O'Doherty, J. P. (2009). A specific role for posterior dorsolateral striatum in human habit learning. *European Journal of Neuroscience*, *29*(11), 2225–2232. https://doi.org/10.1111/j.1460-9568.2009.06796.x

Vaghi, M. M., Cardinal, R. N., Apergis-Schoute, A. M., Fineberg, N. A., Sule, A., & Robbins, T. W. (2019). Action-Outcome Knowledge Dissociates From Behavior in Obsessive-Compulsive Disorder Following Contingency Degradation. *Biological Psychiatry: Cognitive Neuroscience and Neuroimaging*, *4*(2), 200–209. https://doi.org/10.1016/j.bpsc.2018.09.014

Wichmann, F. A., & Hill, N. J. (2001). The psychometric function: I. Fitting, sampling, and goodness of fit. *Perception & Psychophysics*, *63*(8), 1293–1313. https://doi.org/10.3758/BF03194544

Wilson, R. C., & Collins, A. (2019). Ten simple rules for the computational modeling of behavioral data. *PsyArXiv*. https://doi.org/10.31234/osf.io/46mbn
